# Daytime radiative cooling semi-transparent roof tile coating from eco-friendly limestone to bio-based hydroxyapatite for tropical environment

**DOI:** 10.1016/j.isci.2026.115893

**Published:** 2026-04-27

**Authors:** Jittrakorn Udomsin, Patawee Sakata, Worawut Rueangsawang, Wachara Benchaphanthawee, Chayani S. Sarumaha, Kullachate Muangnapoh, Chattrarat Ponghiransmith, Taweesak Kaewmanee, Napan Phuphathanaphong, Jakrapong Kaewkhao, Supakij Suttiruengwong, Worawat Wattanathana, Pongsakorn Kanjanaboos

**Affiliations:** 1 Advanced Technologies for Energy and Sustainability Lab, School of Materials Science and Innovation, Faculty of Science, Mahidol University, Nakhon Pathom 73170, Thailand; 2 Center for Cooling and Energy-saving Materials, Faculty of Science, Mahidol University, Nakhon Pathom 73170, Thailand; 3 Center of Excellence in Glass Technology and Materials Science (CEGM), Nakhon Pathom Rajabhat University, Nakhon Pathom 73000, Thailand; 4 National Metal and Materials Technology Center (MTEC), National Science and Technology Development Agency (NSTDA), Pathum Thani 12120, Thailand; 5 Physics Program, Faculty of Science and Technology, Nakhon Pathom Rajabhat University, Nakhon Pathom 73000, Thailand; 6 Department of Materials Science and Engineering, Faculty of Engineering and Industrial Technology, Silpakorn University, Sanamchandra Palace Campus, Nakhon Pathom 73000, Thailand; 7 Department of Materials Engineering, Faculty of Engineering, Kasetsart University, 50 Ngamwongwan Rd., Ladyao, Chatuchak, Bangkok 10900, Thailand

**Keywords:** Radiative cooling, roof tile coating, calcium-carbonate, hydroxyapatite, building materials

## Abstract

Daytime passive radiative cooling (DPRC) has attracted considerable interest as an energy-efficient strategy to achieve temperature reduction by reflecting incoming solar radiation while emitting thermal radiation. In this work, we developed a semi-transparent (light transmittance = 71.4% ± 0.8%) waterborne polyurethane/nano-micro calcium carbonate composite (WPU/CaCO_3_) with 60 wt% loading via ball milling for heat reflection. Later, CaCO_3_ was replaced with a bio-based hydroxyapatite (HAp) with 50 wt% loading as a durable and sustainable pigment for roof tile coating. The optimized coatings with CaCO_3_ and HAp were applied on the fiber cement roof tiles, thereby achieving a temperature reduction compared with an uncoated roof tile of approximately 5.3°C and 9.6°C under direct sunlight, respectively, without yellowing appearances after sun exposure for both cases. Additionally, coating on other outdoor materials has been demonstrated. Both WPU/CaCO_3_ and WPU/HAp show strong potential as a sustainable solution for radiative cooling semi-transparent paints.

## Highlights

• Calcium carbonate and hydroxyapatite are used for semi-transparent radiative cooling
• Semi-transparency is done via controlled particle size and refractive index matching
• The low-cost coating achieves good stability and excellent adhesion
• Competitive cooling performance can be achieved on various functional materials

## Graphical abstract

**Figure:**
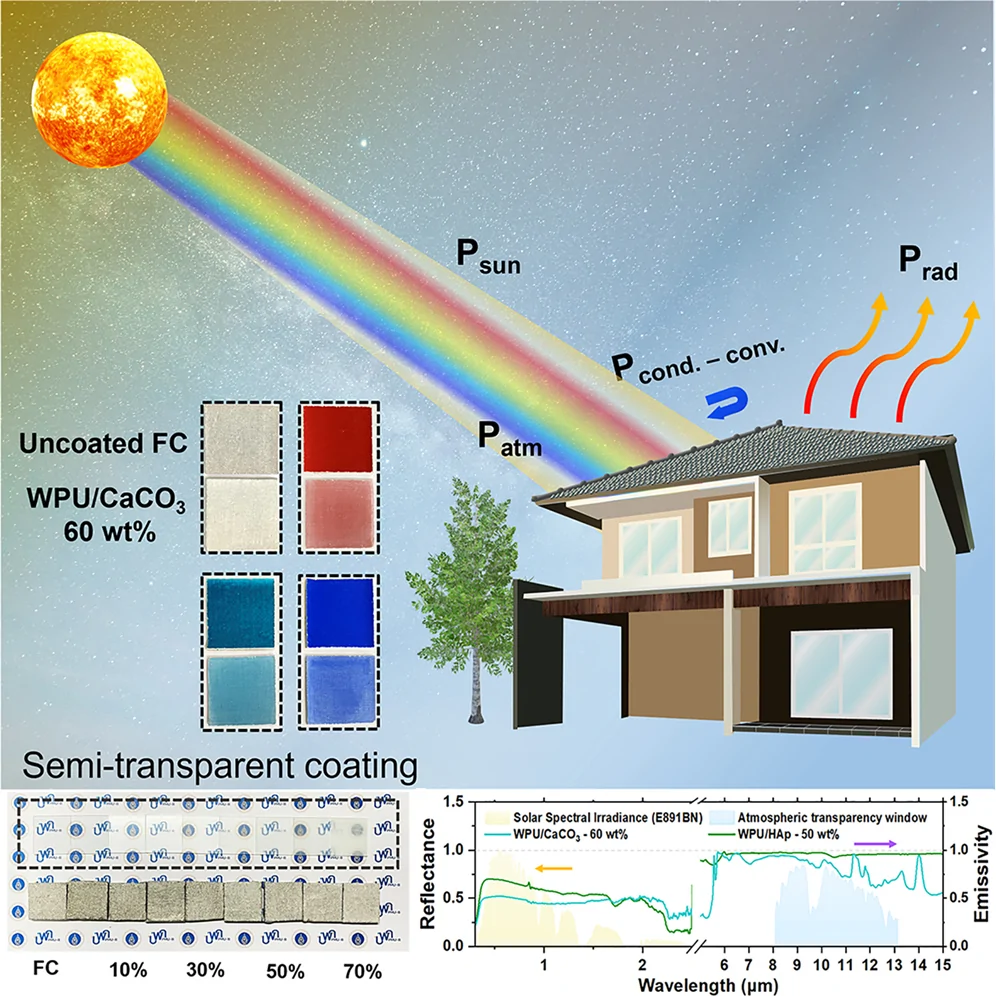

## Teaser

Urban planning; Materials science; Materials chemistry; Material finishing; Materials property

## Introduction

The continuous rise in global temperatures has led to increased reliance on active cooling appliances such as air conditioning systems in buildings and vehicles, leading to increased energy consumption and greenhouse gas emission that further intensifies the cycle of anthropogenic climate change.^1^^,^^2^ Another promising approach is daytime passive radiative cooling (DPRC), a thermal management technique, that dissipates heat to the cold outer space (∼3 K) via mid-infrared (MIR) radiation through the atmospheric transparency window (λ = 8–13 μm), without requiring external energy input, along with solar light reflection to reduce incoming heat flux. This strategy offers a cost-effective, environmentally friendly, and sustainable alternative to conventional cooling methods.^3^^,^^4^ Modes of solar reflection include metallic reflection, multilayer dielectric film reflection, total internal reflection, and Mie scattering, which can be engineered to minimize solar heat gain. Meanwhile, the fundamentals of thermal emissivity, including molecular vibration, phonon-polariton resonance, electromagnetic resonance, and graded-index interface, are critical for enhancing radiative heat dissipation in the infrared (IR) region.^2^^,^^5^^,^^6^

Based on the aforementioned photonic requirements, various materials—metallic, inorganic, and polymeric—have been employed for radiative cooling applications. Although metallic materials exhibit high solar reflectance, their high cost and susceptibility to corrosion significantly limit their long-term outdoor durability. As a result, inorganic-polymeric composite systems have been extensively studied due to their cost-effective and scalable production in industries.^4^^,^^5^ For inorganic particle selection, key parameters, including refractive index (n), extinction coefficient (k), and bandgap energy (E_g_), govern solar photon scattering and absorption, where larger bandgaps favor dielectric and insulating behavior.^4^^,^^7^ Consequently, the material should provide a bandgap beyond the range of solar photon energy (0.49–4.13 eV) to avoid solar radiation absorption.^8^ Common candidates such as titanium dioxide (TiO_2_; n = 2.5, E_g_ = 3.0 eV),^2^^,^^3^ aluminum oxide (Al_2_O_3_; n = 1.76, E_g_ = 7.0 eV),^3^ and barium sulfate (BaSO_4_; n = 1.66, E_g_ = 7.27 eV)^9^ exhibit tunable refractive indices and bandgaps, depending on particle size, shape, and crystalline structure.^10^ Furthermore, hexagonal boron nitride (hBN; n = 2.2, E_g_ = 5.9 eV) particles combined with silane-based binder polymers achieved strong anisotropic reflectivity from the platelet-like morphology.^11^ Moreover, advanced material systems, including photonic architectures and functional inorganic-polymer composites, have been developed to enhance radiative cooling capability, while addressing practical constraints such as durability and environmental exposure.^12^^,^^13^

Currently, there are several literatures, which focus on thermal management under realistic outdoors and tropical conditions, highlighting the critical role of humidity, solar intensity, and convective heat transfer in determining cooling effectiveness.^14^ Recently, the radiative cooling from bio-based materials such as cellulose/nanocellulose, silk cocoon fabric, and chitosan-derived composite aerogel film dopped with melamine-phytic acid have been explored owing to their sustainability and promising cooling performance attributed to inorganic fillers and/or well-designed porous structures.^10^^,^^11^^,^^12^^,^^13^^,^^15^ However, only a few publications about bio-based radiative cooling have reported on mechanical durability and weathering tests, as bio-based materials tend to turn yellow due to oxidation, hydrolyzation, and photodegradation,^16^ undergo degradation under heat and moisture,^17^ or promote fungal growth.^18^ These issues are major concerns that limit their ultimate real-world usage.

An additional noteworthy inorganic candidate is calcium carbonate (CaCO_3_; n = 1.65, E_g_ > 5.0 eV)^3^^,^^19^^,^^20^ due to low cost, non-UV absorption, weathering stability, and high mechanical strength. CaCO_3_ is widely used as a filler in paint, plastic, paper, and construction industries,^21^^,^^22^^,^^23^ which signal their durability and practical implementation. Hence, CaCO_3_ has been extensively incorporated into various polymeric-based materials such as polyvinyl chloride, acrylic paint, and poly(acrylated dipentaerythritol) (PDPHA) for effective radiative cooling.^17^^,^^18^^,^^22^

However, most CaCO_3_-polymer composites possess a dense white coloration, which obscures the underlying substrate and restricts esthetic flexibility. Hydroxyapatite (HAp; Ca_10_(PO_4_)_6_(OH)_2_) has emerged as an alternatively radiative cooling candidate. HAp, known as a bio-ceramic material and main component of skeleton tissue,^24^ is a calcium phosphate compound with enrichment of positive charge from calcium ion and negative charge from phosphate ion, which allow diverse physical and chemical interaction.^25^^,^^26^ Owing to these characteristics, HAp has been widely investigated in biomedical applications, including bone regeneration scaffolds,^27^^,^^28^ drug delivery,^29^^,^^30^ and dental care products.^31^^,^^32^ HAp may not be lower cost than CaCO_3_ due to the purification process that requires calcination and chemical treatment.^33^ However, the process of limestone mining impacts the environment, causing geographical change, air pollution, and depletion of non-renewable resources. In contrast, HAp, derived from animals such as bovine-bone, fish-bone, and seashell as daily food waste, represents as an alternative choice for radiative cooling applications.

Polydimethylsiloxane (PDMS), poly(vinylidene fluoride) (PVDF), and poly(methyl methacrylate) (PMMA) have been widely reported for radiative cooling applications (Figure S1).^34^ However, these polymers are rarely commercialized in paints. In contrast, non-toxic waterborne polyurethane (WPU; n = 1.65), containing the –NH– and C=O functional groups, allows more bonding and better adhesivity on various substrates, which has widely been used in coating both in academics and industries.^35^^,^^36^ Additionally, WPU has emissivity in range of atmospheric transparency windows due to its abundant functional groups.^34^^,^^37^ The polymer structure can be readily modified or composited to achieve high solar reflectance,^38^ enhanced emissivity,^39^ optical transparency,^36^ environmental stability,^40^ and mechanical robustness.^41^

Most of the radiative cooling paints focus on super white paint,^42^^,^^43^ which obscures the substrate and lacks artistic diversity. In previous studies, transparent radiative coating via spin coating^44^ or plasma-enhanced chemical vapor deposition (PECVD)^45^ exhibits advantages in presenting the original surface or background. However, they are limited by high production costs and challenges in large-scale expansion. From our investigation, we did not identify any semi-transparent paint for radiative roof tile coatings that is both cost-effective and meet the requirements of practical commercial use. In this study, we developed a semi-transparent WPU/CaCO_3_ coating for rooftop cooling applications. In particular, CaCO_3_ can be replaced by bio-based HAp. The WPU/CaCO_3_ and WPU/HAp coating were fabricated via ball milling method, while the coating was performed via industrial-standard brush painting. The combination of index matching between the embedded particles and the polymer matrix, nano-micro-particle size distribution, homogenous mixing via ball milling, and layer-by-layer coating technique allows this work to achieve both high transparency and high particle loading for effective radiative cooling.

## Results and discussion

### Design and optimization of CaCO_3_ concentration in WPU

Heat reflective coating materials have been developed for thermal management on buildings and vehicles as a sustainable and energy saving solution. Coating roof tile with heat-reflective paint significantly reduces the amount of heat transfer into the building. Consequently, the cooling load on air conditioning systems is alleviated, thereby promoting energy conservation. Figure 1A represents fabrication method of WPU/CaCO_3_. Briefly, different amounts of CaCO_3_ were dispersed in commercial WPU via ball milling to achieve particle size reduction and uniformity along with homogeneous mixture. The particle sizes of CaCO_3_ before and after ball milling centers around 4.0 ± 0.1 and 2.9 ± 0.1 μm, respectively (Figure S2). Then, the WPU/CaCO_3_ semi-transparent paint was coated on the roof tile by utilizing a paint brush by a layer-by-layer method for facile application which is available in the market. The specific particle size and the layer-by-layer deposition, which ensures homogeneous particle distribution in the vertical direction, provide broad reflection and enhanced optical transmittance,^34^ thereby boosting a semi-transparent appearance. Furthermore, WPU/CaCO_3_ coating undergoes ambient drying without requiring post-processing, which is advantageous for real-world usage. Notably, CaCO_3_ particles function as ultraviolet-visible-near-infrared (UV-vis-NIR)-reflective and IR-emissive components, whereas WPU acts as both the polymer binder and a mid-IR radiation emitter. Additionally, our WPU/CaCO_3_ is developed for high environmental durability, particularly for rooftop and automotive coating. Figure 1B illustrates the glass slide and fiber cement roof tile surfaces coated with WPU/CaCO_3_ at varying CaCO_3_ contents ranging from 0 to 70 wt%.

**Figure 1:**
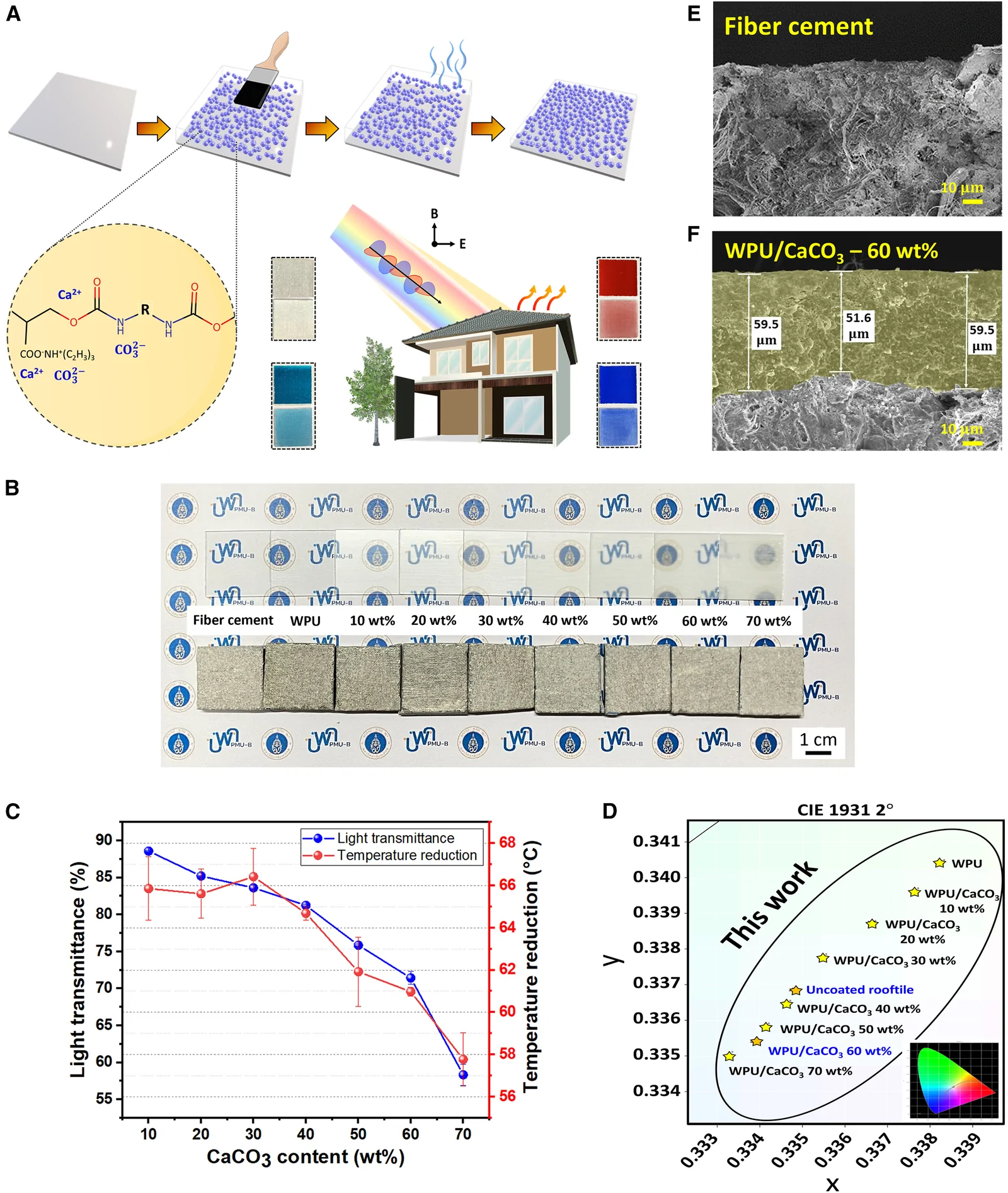
Fabrication process, application, and physical characterization (A) Schematic illustration of the fabrication process of the semi-transparent WPU/CaCO_3_ coating and its application on a roof tile via brush painting for radiative cooling purposes. (B) A photograph demonstrating the transparency of the WPU/CaCO_3_-60 wt% coating on both a glass slide and a roof tile (scale bars represent 1 cm). (C) The plot of light transmittance and temperature performance under various CaCO_3_ contents of the coating under 1 sun illumination. (D) A CIE 1931 chromaticity diagram of WPU/CaCO_3_ with concentrations ranging from 0 to 70 wt%. (E) FESEM images of uncoated roof tile. (F) FESEM images of WPU/CaCO_3_-60 wt%-coated roof tiles. Scale bars represent 10 μm.

The optical transmittance of WPU/CaCO_3_ coated glass, evaluated under a sun simulator for 1 sun (1,000 W m^−2^) within the 400–1,100 nm range, is shown in Figure 1C. It can be observed that uncoated and WPU-coated glass slides exhibit comparable transmittance at 90.0% ± 0.1%, indicating that the WPU is optically transparent and does not significantly contribute to light scattering. Meanwhile, the incorporation of CaCO_3_ into the WPU/CaCO_3_ (10–70 wt%) composite results in a decrease in light transmittance from 88.6% ± 0.1% to 58.3% ± 1.4%, which can be attributed to increased light scattering caused by the CaCO_3_ particles. The addition of CaCO_3_ particles with sizes comparable to the wavelength of incident light induces Mie scattering, which accounts for the observed optical diffusion. Furthermore, since CaCO_3_ possesses a refractive index comparable to that of WPU, the deflection of light upon interacting with the particle boundaries within the matrix is less pronounced. As a result, the coating strategy becomes clearer compared to previous studies for the same CaCO_3_ loading.^19^ In addition, the color characteristics of the fiber cement roof tile coated with the WPU/CaCO_3_ were evaluated by using a UV-vis spectrophotometer, as illustrated by the CIE 1931 chromaticity diagram in Figure 1D. All samples were located within the neutral white zone, exhibiting only minimal variations in shade. The uncoated fiber cement roof tile is positioned on green-blue region (0.335, 0.337). Once coating WPU on the fiber cement, the chromaticity coordinates are right shifted to yellow region (0.338, 0.340). As the CaCO_3_ content increases to 70 wt%, the chromaticity coordinates are gradually left shifted from the yellow to blue regions (0.333, 0.335), exhibiting increased brightness and enhanced long wavelength reflection. Figures S3 and S4 presents the field emission scanning electron microscope (FESEM) images of the uncoated and coated fiber cement roof tile under different CaCO_3_ loadings. As shown in Figure 1E, The uncoated roof tile shows rough morphology, while applying the pristine WPU coat on roof tile yields the smooth surface. Increasing the CaCO_3_ coating ratio from 10 to 70 wt% leads to more particle appearances. Too much loading at 70 wt% causes the appearance of microcracks, as clearly revealed in the cross-sectional image (Figure S4I). Therefore, the 60 wt% CaCO_3_ was selected as an optimal condition as presented in Figure 1F. Furthermore, the thicknesses of the WPU/CaCO_3_ at 60 wt% from 1 to 5 coating layers were analyzed as shown in Figures S5A–S5F. The thickness of the first coating layer is 12.3 ± 1.0 μm, gradually increasing to 56.4 ± 2.2 μm by the fifth layer, with an average thickness increment of 11 μm per layer.

### Optical properties of WPU/CaCO_3_

Figure 2A displays the reflectance spectra of the WPU/CaCO_3_-coated fiber cement roof tile over a wavelength range from 200 to 2,500 nm using barium sulfate as a reference material, being calculated as percentile according to ASTM E903-96. The uncoated fiber cement roof tile reflects an incident light in the UV region (300–400 nm), visible light (vis) (400–700 nm), and IR region (NIR) (700–2,500 nm) about 43.6%, 47.5%, and 42.9%, respectively. With the presence of WPU coating, the reflectance in UV region is decreased to 13.5% due to UV absorber in WPU,^46^^,^^47^ which protects the coated surface as well as its original color as found in our experiment on wood coating. Meanwhile, the presence of CaCO_3_ at 60 wt% results in the increase of the optical reflectance across the UV-vis-NIR spectral range, reaching 45.3%, 51.4%, and 46.2%, respectively, with a weighted average value of 48.2%, boosting the daytime radiative cooling requirement by reflecting the high-intensity photon regime to avoid excessive incoming heat. This improvement is stemmed from suitable particle size and distribution, which enhance Mie scattering. The reflectance spectra of all samples and the corresponding summary of reflectance values are presented in Figure S6A and Table S1.

**Figure 2:**
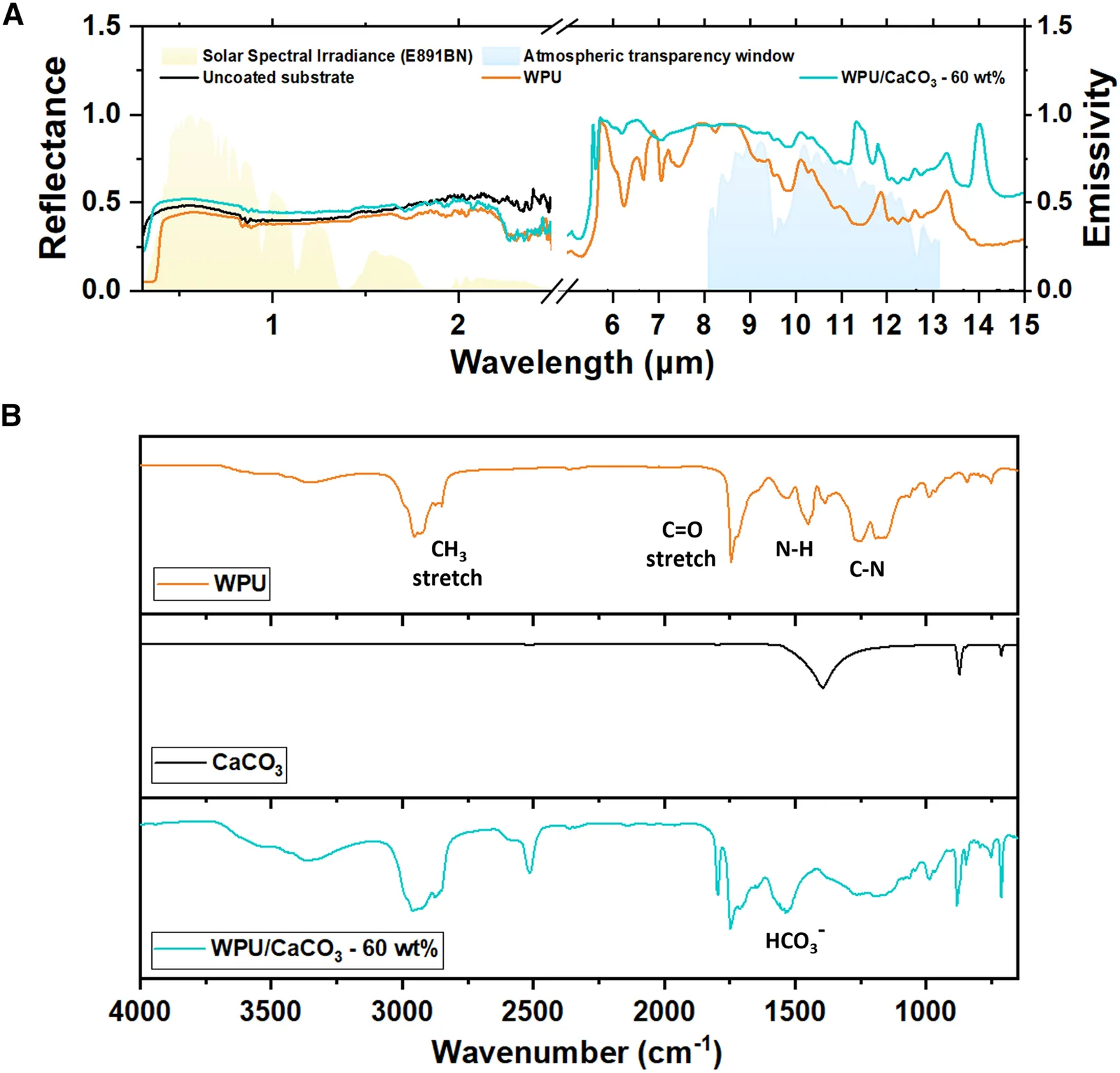
Optical properties and chemical characterization (A) Solar reflectance and emissivity spectra of the uncoated fiber cement, WPU, and WPU/CaCO_3_-60 wt%. (B) ATR-FTIR spectra of the WPU, CaCO_3_, and the WPU/CaCO_3_-60 wt%. The highlighted zone indicates the atmospheric transparency window range (8–13 μm).

The mid-IR emissivity spectra extending wavelength across 8 to 13 μm can be measured by Fourier transform infrared (FTIR) (wave number: 770 to 1,250 cm^−1^), attributing to the vibrational resonance of the polymer molecules and aligning with the atmospheric transparency window. The FTIR spectrometer with the integrating sphere mode was utilized to analyze scattered IR materials like reflective paint or powder. However, the measurement of bare roof tile could not be performed due to its curvy and thick nature. For such measurement, pristine WPU and WPU/CaCO3 at 60 wt% had to be coated on aluminum plate, yielding emissivity values in Figures 2A and S6B. The FTIR spectra in Figure 2B display the characteristic absorptions of polyurethane (R–HNCOO–R′)_n_ at 2,956, 1,740, 1,540, 1,250, and 1,170 cm^−1^, which are corresponding to the vibrational modes of CH_2_ stretching, C=O stretching, N–H bending, C–N stretching, and C–O–C stretching, respectively (orange line).^36^^,^^48^^,^^49^ Meanwhile, pristine CaCO_3_ particles exhibit three distinct absorption bands at 1,400, 871, and 712 cm^−1^, relating to the asymmetric stretching, out-of-plane bending, and in-plane bending modes of the CO_3_^2−^ anion, respectively (black line). Upon addition of 60 wt% CaCO_3_ into the WPU matrix (green line), an additional absorption peak appears at 2,525 and 1,535 cm^−1^, which can be attributed to the stretching of the bicarbonate (HCO_3_^−^) anion^50^^,^^51^ from the transformation of CO_3_^2−^ to HCO_3_^−^ species in the aqueous medium previously reported in the literature.^52^^,^^53^^,^^54^ Although the formation of HCO_3_^−^ is unavoidable due to the pH conditions of the waterborne system, either CO_3_^2−^ or HCO_3_^−^ form via the incorporation of CaCO_3_ remains secondary for enhancing emissivity in the IR regime according to the small footprint in the atmospheric zone highlighted in Figure 2B.

### Outdoor cooling performance

An outdoor cooling performance test demonstrates the full day temperature reduction at Mahidol University, Nakhon Pathom, Thailand (N 13.79, E 100.32), on April 23–25, 2025; the experimental setup and testing boxes are illustrated in Figures S7A and S7B, and full-day temperature profiles are shown in Figure 3A for 3 consecutive days. The specimens with CaCO_3_ concentrations varying from 0 to 70 wt% were mounted inside closed testing chambers with three repeating boxes for each condition. A K-type probe was introduced at a depth of 0.5 cm beneath the sample surface to record temperature variations (system temperature). The increasing volume fractions of CaCO_3_ particle leads to an enhancement of both solar reflection and emissivity, especially in UV region from 13.5% to 48.2% and emissivity from 66.2% to 83.9%, respectively, as summarized in Table S1. Additionally, the temperature profiles in Figure 3B illustrate the internal system temperatures of all samples on day 1 (April 23, 2025). At the time between 11:30 a.m. and 12:30 p.m., the solar intensity reached its peak of 1,000–1,100 W m^−2^ under cloud-free conditions, with an average ambient temperature of 37.7°C ± 0.9°C and an average wind speed of 0.9 ± 0.5 m s^−1^. The temperature inside the empty boxes was recorded to be at 55.0°C ± 1.5°C as a reference (average from 11:30 a.m. and 12:30 p.m.). The deployment of an uncoated fiber cement leads to a marked rise in system temperature, reaching 64.3°C ± 1.8°C, which is ascribed to the progressive thermal accumulation within the fiber cement roof owing to its high solar absorptance and limited thermal emissivity.^55^ When WPU was applied on the roof tile surface, the system temperature rises to 64.8°C ± 1.8°C in agreement with the reflectance and FTIR results. As the CaCO_3_ volume fraction increases from 10 to 70 wt%, the system temperatures decrease from 64.7°C ± 1.7°C to 57.0°C ± 1.2°C, respectively. A critical threshold at 40 wt% produces an average system temperature below that of the uncoated fiber-cement control. WPU/CaCO_3_-70 wt% shows the most system temperature reduction, yet the filler becomes overloaded and the coating shows signs of cracking, not suitable for real usage. In brief, 60 wt% WPU/CaCO_3_ represents the daytime system temperature lower than those of the uncoated fiber cement and the WPU coating at 4.2°C and 4.7°C, respectively. This is supported by higher solar reflectance of 48.2% and higher mid-IR emissivity of 83.9%. Broadband particle size distribution from 1 to 10 μm (Figure S2) could scatter incident protons from wide wavelengths within the solar spectrum due to Mie scattering effect, which promotes the outward reflection of thermal radiation along with more emissivity in the atmospheric window; both of which reduces heat accumulation in the composite.^1^^,^^56^ Moreover, the conductive heat transfer coefficient rises with increasing volume fractions as a result of the greater inter-particle contact area; in contrast, the radiative heat transfer coefficient associated with particle size exerts little influence at large volume fractions. This is associated with the shading effect by neighboring particles in specific bulk volume.^56^ As depicted in Figures S8A–S8C, the radiative power (P_rad_) and the net cooling power (P_net_) of WPU and WPU/CaCO_3_-60 wt% show that the addition of CaCO_3_ contributes to an increase in radiative power, thereby enhancing the net cooling power. For example, WPU shows a P_rad_ of 207.5 W m^−2^ and P_net_ of −450 W m^−2^ at the atmospheric temperature (∼35°C), which is related to heat absorption from solar irradiance and heat re-absorbed from the atmosphere together with heat conduction and convection. Besides, the WPU/CaCO_3_-60 wt% shows both enhancement in P_rad_ and P_net_ at the atmospheric temperature with values of 274.8 W m^−2^ and −370 W m^−2^, which is attributed to the enhanced reflectance and emissivity that facilitates greater radiative heat dissipation, resulting in a lower roof temperature. However, the real temperature of ∼50°C in a closed test box has to be considered as ambient due to the internal heat accumulation inside the closed system; the WPU/CaCO_3_-60 wt% shows an even greater cooling power of −231.2 W m^−2^ due to the stronger radiative power of 338.7 W m^−2^. Moreover, IR thermography images capture the thermal emission characteristics of each sample, highlighting both the upper surfaces under solar exposure and the surfaces beneath the roofing structure. Progressive reduction in both upper and lower surface temperatures with increasing filler content is clearly evident, aligning closely with internal test-box temperature measurements and trends observed in reflectance and emissivity spectra, thereby confirming the coatings’ enhanced radiative cooling capability as presented in Figure S9.

**Figure 3:**
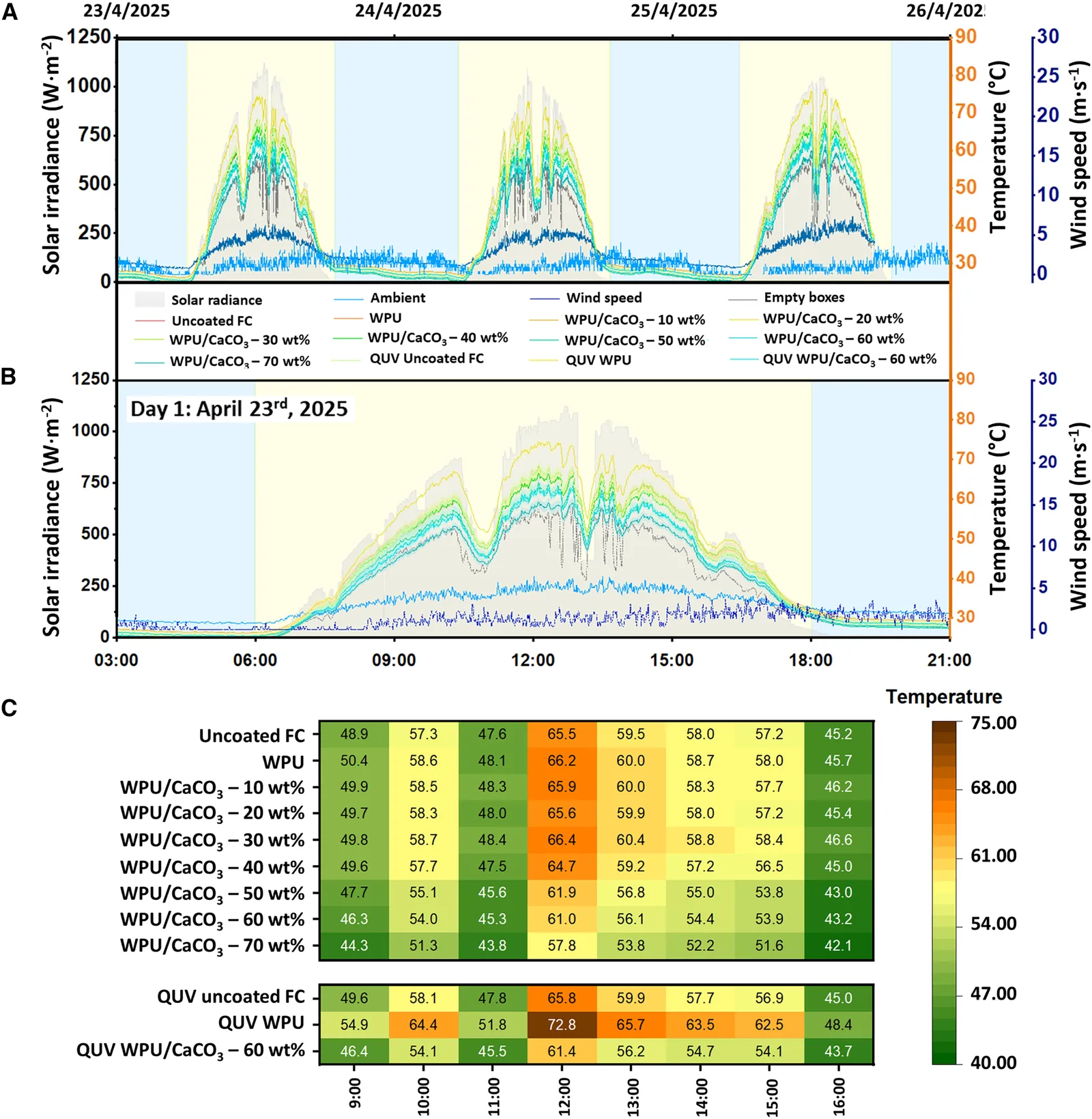
Evaluation of the thermal reduction performance (A) Real-time system temperature measurements for 3 days. (B) Detailed temperature profile on the first day of samples with varying CaCO_3_ concentrations (0–70 wt%) in WPU and QUV-tested samples (uncoated roof tile, WPU, and WPU/CaCO_3_-60 wt%), recorded on April 23–25, 2025, in Nakhon Pathom, Thailand. Three replicates of the 8 × 8 cm^2^-sized samples were tested to obtain the statistical data. The results are presented as the mean (central line) with standard deviation (SD) (shaded contour region). (C) A heatmap of system temperatures on the first day.

Weathering stability of the coating layer was conducted via QUV accelerated weathering tester in accordance with ASTM G 154. Each cycle involves UV irradiation (UVA: 340 nm) at 0.89 W m^−2^ and temperature of 60°C for 8 h, followed by condensation at 50°C under dark mode for 4 h, for a total test duration of 168 h (7 days). Photographs of coated roof tiles before and after QUV are depicted in Figure S10. For comparison, the outdoor system temperatures of the uncoated roof tile, the WPU coating, and the WPU/CaCO_3_ at 60 wt% after QUV treatment were evaluated on April 23^rd^ to 25^th^, 2025 (Figure 3A); temperature profile and heatmap analysis of day 1 are demonstrated in Figures 3B and 3C. After QUV, temperature reduction performance of all samples at the noon time is slightly decreased, even for the uncoated fiber cement (65.5°C → 65.8°C). The optical microscopic images of the uncoated roof tile after QUV exposure reveal physical degradation and more pronounced roughness (Figure S11A). Unexpectedly, the WPU coating after QUV exposure exhibits a significant system temperature increase of 6.6°C (66.2°C → 72.8°C). The optical microscopic images show negligible weathering on the WPU coating. However, this finding suggests that UV absorption within the WPU itself may actually accelerate the polymer’s degradation (Figure S11B). On the contrary, the QUV WPU/CaCO_3_-60 wt% can retain most of the system temperature reduction efficiency, yielding 4.1°C lower than uncoated roof tile compared to 4.5°C of the freshly prepared sample and 11.4°C lower than that of the WPU roof tile after QUV. From the optimal microscopic image, WPU/CaCO_3_-60 wt% exhibits sufficient weathering resistance under UV irradiation and simulated rain exposure (Figure S11C), representing a significant step toward to practical application.

### Mechanical durability, chemical resistance, and anti-fouling properties

An additional concern involves the scratch resistance of the coatings, especially important for commercial transportation and installation. Owing to the excessive rigidity of our roof tile coatings, conventional polymer hardness measurements (e.g., Shore D hardness and sandpaper abrasion tests) could not be applied, since they pose a risk of damaging the measurement instruments. Therefore, the Mohs hardness test was employed to determine the coating’s hardness and to benchmark its performance against those of commercial roof tiles as presented in Figure S12. The original surface of the commercial roof tile shows Mohs hardness of no. 3, in agreement with a standard of cement hardness, its main component. Whereas, WPU/CaCO_3_-60 wt% coated roof tile shows Mohs hardness between no. 3–4 as the Mohs pen no. 4 could only make negligible scratching on the coating due to polymer stiffness. This indicates that the coating can protect the tile surface against scratching more effectively than conventional tiles. Furthermore, the WPU/CaCO_3_-60 wt% coated roof tile was immersed in aqueous solutions with different pH values of 2, 4, 6, 8, 10, 12, and 14; the samples underwent cross-cut tests after 1–7 days to investigate the adhesion performance over times and stimulate the resistance against cleaning agents under harsh conditions as shown in Figure 4A. All the one hundred fifty cut specimens exhibit excellent adhesion with negligible peeling even after being submerged in the extreme pH 2 and pH 14 solutions for 7 days, therefore the developed coating easily passes the harsh environmental test and shows potential for commercial usage. After cross-cutting and peeling with 3M Scotch filament tape, the cut areas remain fully adhered to the roof tile surface as evidenced in Figure S13. As the coating in real usage may experience dust accumulation from high pollutant emission sources like transportation activities, agricultural burning, and rainwater. The anti-fouling performance of uncoated roof tile, WPU, and WPU/CaCO_3_-60wt % was evaluated via a typical protocol as employed in previous study.^57^ The tiles were placed at a 30° inclination, simulating the natural slope of residential rooftops. Ten grams of volcanic soil (red powder) with a particle size smaller than 1 mm was sprinkled onto the specimen until the surface was completely covered. Subsequently, 1 L of purple-dyed water was poured over the sample to mimic the washing action of rainfall. Optical microscopy images were recorded before and after the test, as shown in Figure 4B. For the uncoated roof tile, the red dust remained on the surface after the protocol and some amount of the colored water was absorbed by the porous roof tile. Meanwhile, the WPU-coated roof tile exhibited a clean surface after the dust-contamination and rain-cleaning simulation. However, residual water droplets remained on the surface after rinsing, which may lead to the formation of dust rings or streaks upon drying, leaving visible marks that compromise surface esthetics. Interestingly, the WPU/CaCO_3_-60 wt% coated roof tile exhibits good self-cleaning behavior, as densely adhered dust was thoroughly removed by natural rainfall. Owing to the homogeneous surface wetting, no residual water marks were left, thus preserving both the uniform color and the esthetic integrity of the surface after drying. In our case, the self-cleaning mechanism is achieved by hydrophilic (water-spreading) surface characteristics, as shown by the water contact angle (WCA) in Figure S14. The uncoated fiber cement shows superhydrophilic property with WCA of about 5.2° due to the hydrogen bonding between the water droplet and open porous structure of fiber cement. After coating with WPU, WPU/CaCO_3_, or the WPU/CaCO_3_-60 wt%, the fiber cements still process hydrophilic property. WPU/CaCO_3_ provides good coverage, having fewer changes in WCA due to water being absorbed into the cement matrix. Upon exposure to rainfall, the dust comprising of mostly hydrophilic particulates such as SiO_2_,^58^ CaSO_4_,^58^ Fe_2_O_3_,^58^ and various metal oxides^57^ forms hydrogen bonds with the incident water droplets and is efficiently transported along the flow path.

**Figure 4:**
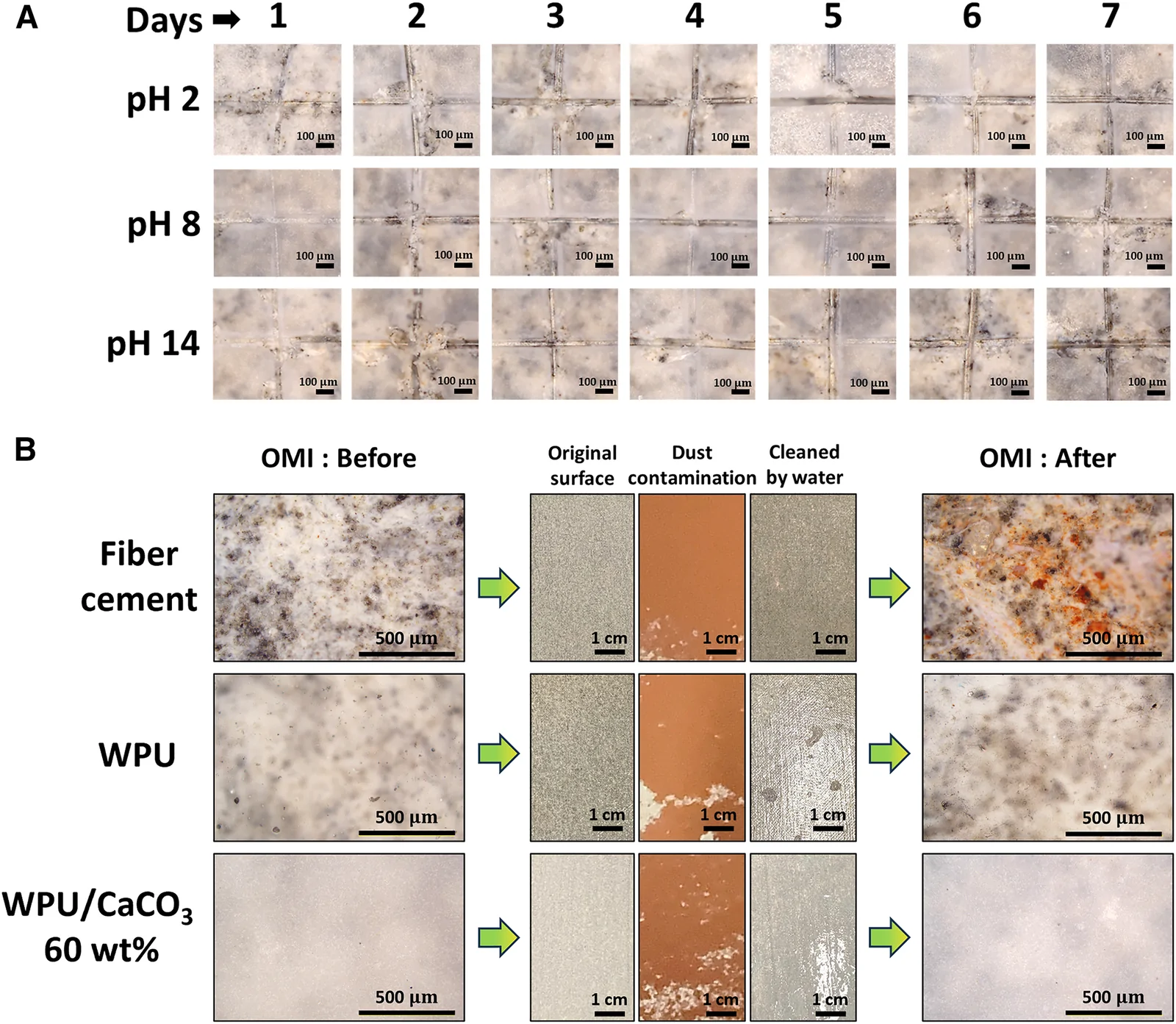
pH Stability and dust-washability (A) Optical microscopic images of samples immersed in solutions with pH 2, 8, and 14, followed by cross-cut adhesion testing every 24 h for 7 days. (B) Optical microscope images and corresponding photographs of the samples before and after the anti-fouling test, where volcanic ash was deposited on a surface inclined at 30° and subsequently rinsed with water to simulate dust accumulation and rainfall.

### Applications

To assess the real-world thermal management performance, the semi-transparent WPU/CaCO_3_-60 wt% was coated onto three representative groups of substrates: (1) roof tile of different colors, (2) automotive stickers, and (3) multiple types of construction materials to investigate the coating’s effectiveness in lowering heat buildup across common outdoor materials encountered in daily life as presented in Figure 5A. For color roof tiles, the semi-transparent coating preserves the original color tone with a slight whitish filter, while enhancing heat reduction and durability under outdoor conditions as shown in Figure 5B; the CIE values are listed in Table S2. The comparative system temperature profiles beneath blue, green, and red roof tiles with and without the coating are shown in Figures 5C–5F and S15. Outdoor measurements conducted on June 8, 2025 (11:30 a.m.–1:30 p.m.; average ambient temperature of 35.3°C ± 0.4°C and empty box system temperature of ∼50°C ± 0.4°C) reveal that uncoated gray, red, green, and blue roof tiles reach 59.9°C ± 1.4°C, 63.8°C ± 1.2°C, 67. 4°C ± 0.3°C, and 68.7°C ± 2.6°C, respectively. After coating, the system temperature reductions of 4.5°C (gray), 3.0°C (red), 4.6°C (green), and 3.0°C (blue) are observed illustrating WPU/CaCO_3_-60 wt% potential across differently colored roofs.

**Figure 5:**
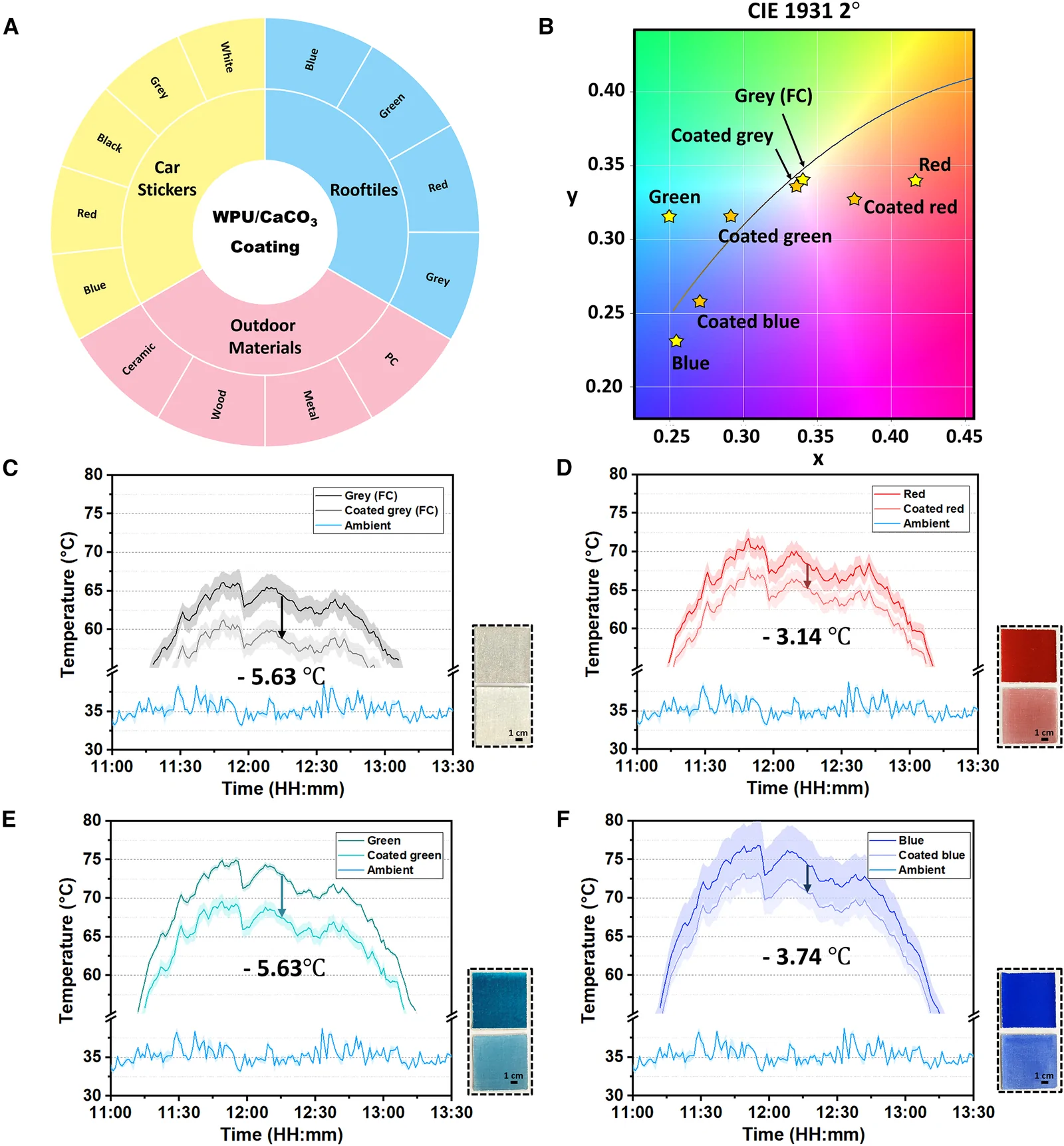
Application and influence of substrate coloration on the thermal reduction performance of WPU/CaCO_3_-60 wt%-coated outdoor materials (A) An overview of the semi-transparent WPU/CaCO_3_-60 wt% coating applied on diverse substrates, including fiber cement, automotive stickers, and outdoor housing materials. (B) A CIE 1931 chromaticity diagram showing the color coordinates of red, green, and blue roof tiles before and after coating. (C–F) Maximum system temperature profiles and corresponding cooling performance of coated roof tiles in gray (C), red (D), green (E), and blue (F), demonstrating the heat-reduction capability of the WPU/CaCO_3_-60 wt% coating (scale bars represent 1 cm).

According to the solar energy ratio distribution: UV (<380 nm, energy ratio 3%–5%), vis (380–780 nm, energy ratio 42%–43%), and NIR (780–2,500 nm, energy ratio 52%–55%)^1^ reflectance in vis and NIR regions is critical in thermal regulation. The heatmap and reflectance results, presented in Figures S16 and S17, show that, at noon, outdoor system temperatures rank from lowest to highest as gray, red, green, and blue, with uncoated system temperatures of 63.4°C, 67.4°C, 71.6°C, and 73.5°C, respectively. The application of the coating results in system temperature reductions ranging from 3.2°C to 5.3°C, which correlate well with the corresponding visible and near-IR average reflectance: gray (55.5% for vis and 50.2% for NIR), red (21.9%, 49.1%), green (21.6%, 31.9%), and blue (19.2, 41.8%) (Figure S17; Table S3).

Besides, the automotive stickers of different colors with and without coating were applied onto a 2-mm-thick aluminum sheet, which represents a car metal frame. On August 8, 2025, the full day system temperature profile and heatmap are shown in Figures S18 and 6A. Without coating, the system temperature ranking from coolest to warmest is white > pristine aluminum > gray > red > blue > black. Since over 50% of automotive fuel is consumed by air conditioning for cooling,^1^ the application of passive radiative cooling coatings to reduce heat is of critical importance. During the peak heating period (10:30 a.m.–12:30 p.m.; an average ambient temperature of 35.0°C ± 0.3°C and empty box temperature of ∼48.7°C ± 0.2°C), the system temperatures of pristine aluminum, white, gray, black, red, and blue are 49.5°C ± 0.4°C, 45.0°C ± 0.6°C, 51.5°C ± 1.0°C, 61.1°C ± 1.0°C, 55.1°C ± 1.0°C, and 58.3°C ± 0.3°C, respectively. With WPU/CaCO_3_-60 wt% coating, three conditions show no system temperature reduction (white: 0.0°C, aluminum: +1.8°C, and gray: +0.7°C). In contrast, dark-toned colors show significant system temperature reduction (black: −5.4°C, red: −4.3°C, and blue: −5.7°C) as shown in Figure S19. IR thermal images (Figure S20) show consistent trends with the temperature measurements. The full reflectance profiles (Figure S21) further confirm that the WPU/CaCO_3_-60 wt% coating is effective for reducing heat for colors with poor reflectivity or strong solar absorption.

**Figure 6:**
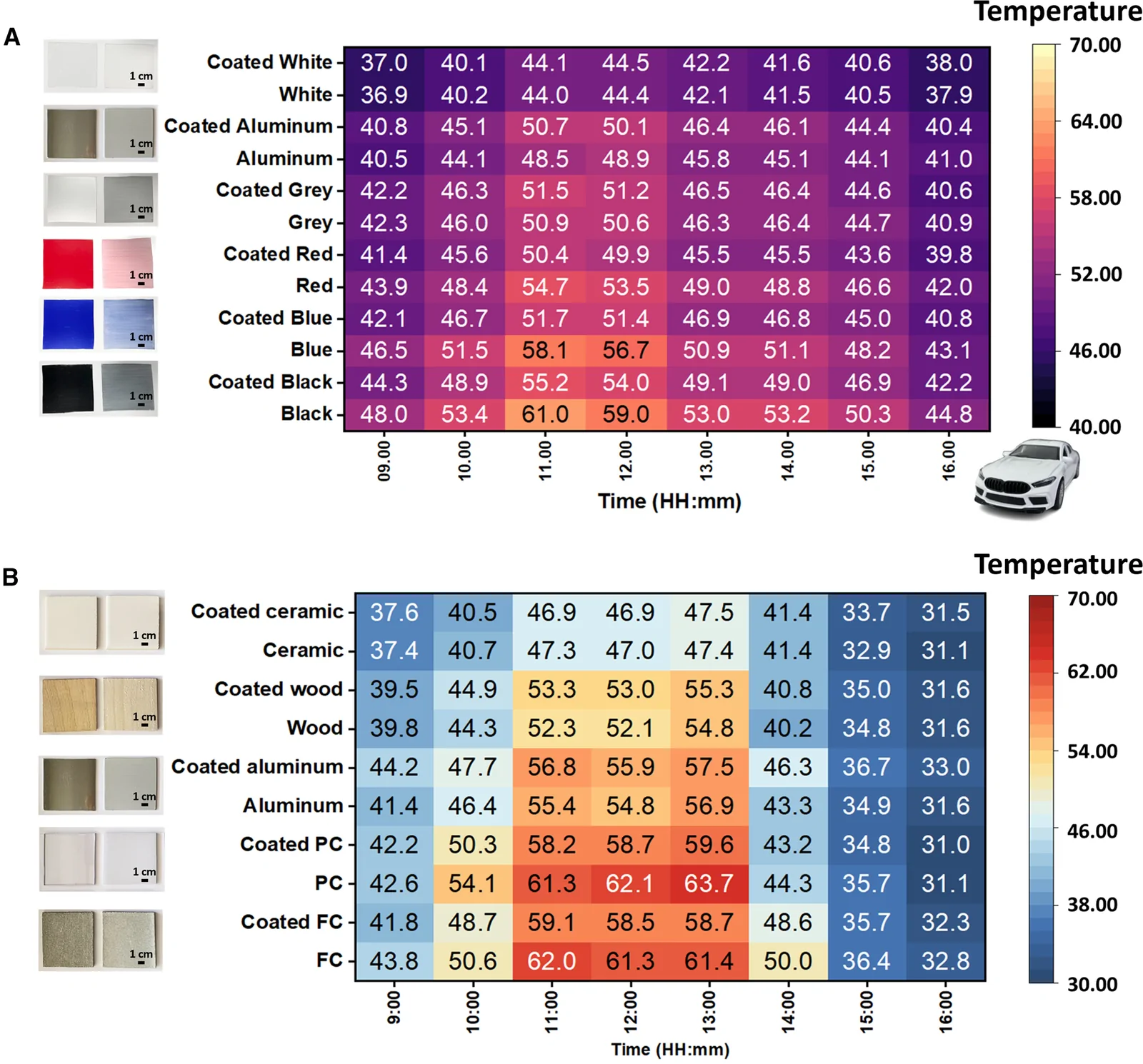
Heatmap analysis of the semi-transparent WPU/CaCO_3_-60 wt% coating applied on different substrates (A) Simulation of heat reduction on aluminum car body panels and commonly used car stickers in white, gray, black, red, and blue (scale bars represent 1 cm). (B) Application of WPU/CaCO_3_-60 wt% on outdoor housing materials, including ceramics, wood, metal, plastic, and fiber cement, highlighting its adaptability across multiple substrate types (scale bars represent 1 cm).

Moreover, several outdoor building materials commonly used in housing construction and subjected to solar irradiation were coated with WPU/CaCO_3_-60 wt% and tested for the system temperature reduction on June 21, 2025. The full-day system temperature profiles and corresponding heatmap are presented in Figures S22 and 6B, respectively; without coating, the coolest (ceramic) and warmest (fiber cement) materials exhibit a system temperature difference of ∼15°C within the same period. During the highest solar intensity duration (10:30 a.m.–12:30 p.m.; average ambient temperature of 35.2°C ± 0.2°C and empty box temperature of ∼52.3°C ± 0.3°C), the materials can be ranked in ascending order of system temperature reduction after coating as follow: ceramic (46.4°C ± 0.5°C → 46.7°C ± 0.1°C), wood (52.5°C ± 0.7°C → 51.6°C ± 0.1°C), metal (55.7°C ± 1.6°C → 54.6°C ± 0.5°C), polycarbonate (PC) (60.5°C ± 1.0°C → 57.3°C ± 2.5°C), and fiber cement (61.0°C ± 0.9°C → 58.3°C ± 0.4°C) as shown in Figure S23. The heat-reduction properties are linked with their corresponding reflectance values as shown in Figure S24 and Table S3. Moreover, the cellulose fibers of wood exhibit pronounced yellowing after 1 week of solar exposure; in contrast, the WPU/CaCO_3_-60 wt% coated wood can preserve its original surface characteristics, as illustrated in the Figure S25. The surface morphologies of various substrates before and after coating are presented in Figure S26, demonstrating that WPU/CaCO_3_ (60 wt%) provides full coverage across different surface types. Moreover, those samples were immersed in water for 7 days, and cross-cut test was performed on days 1, 3, and 7 (Figure S27). Porous substrates such as fiber cement and wood exhibit excellent adhesion with nearly 0% area removal, while non-porous aluminum and PC show moderate removal (5%–15%). In contrast, the dense and smooth ceramic surface results in complete coating removal after 1 day of immersion.

### The utilization of bio-based HAp as a superior alternative to limestone-derived CaCO_3_

HAp represents hexa-functional active sides of phosphate group and nine active sides of calcium ion,^25^^,^^26^ which suggest an enhanced bonding affinity with WPU, thereby increasing the viscosity and reducing the required dosage. A short comparison between CaCO_3_ and HAp particles were performed using FESEM, dynamic light scattering (DLS), and FTIR. The CaCO_3_ particle exhibits crystalline layers larger than 5 μm (Figure S28A), which are fractured into smaller fragments after the ball-milling process (Figure S28B). In contrast, HAp particle shows aggregated clusters of irregularly shaped particles with submicron to micron-scale dimensions. The heterogeneous texture, characterized by sharp edges and interconnected structures, indicates high surface area, which could promote interfacial interactions (Figure S28C). After ball milling, HAp particle is further transformed into irregularly shaped particles smaller than 1 μm, thereby providing an even larger specific surface area (Figure S28D). In addition, HAp before and after ball milling has sizes of 6.0 ± 0.1 and 3.0 ± 0.1 μm, respectively (Figure S29). Furthermore, the HAp particle shows the sharp absorption peaks at the wave numbers of 560 cm^−1^ and 1,020 cm^−1^, which refer to the asymmetric bending and stretching of the ${\text{PO}}_{4}^{3-}$ group,^59^^,^^60^ which possesses higher absorption in the long-wave infrared (LWIR) region than that of CaCO_3_ powder and provides enhanced emissivity in the atmospheric window range, as shown in Figure 7A. Moreover, the nano size of HAp and the more refractive index differences for WPU/HAp compared to WPU/CaCO_3_ can enhance the scattering efficiency in the broadband wavelength.^34^

**Figure 7:**
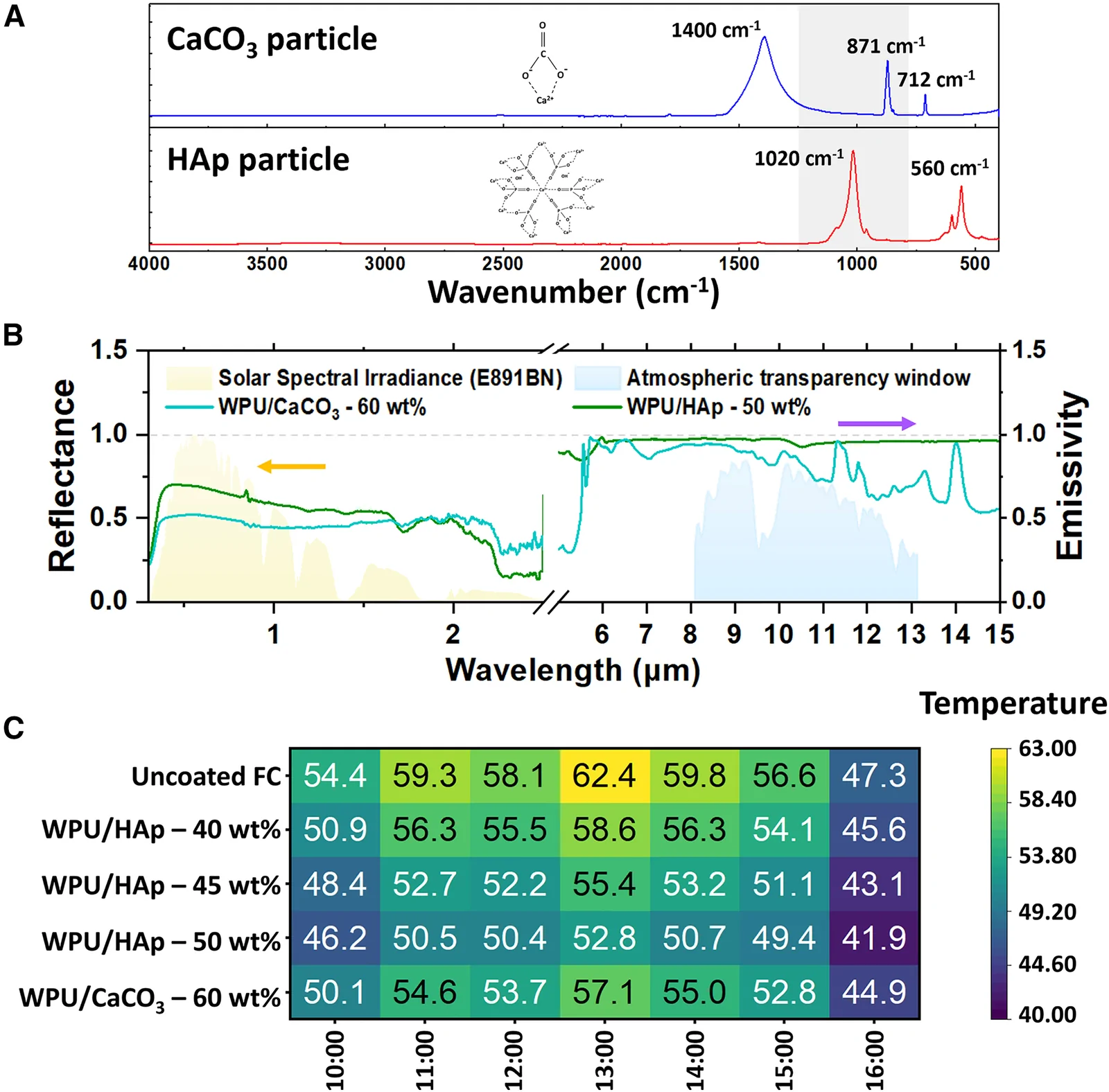
Chemical characterization, optical properties, and thermal reduction performance (A) ATR-FTIR of the CaCO_3_ particle and HAp particle. (B) A comparison of solar reflectance and emissivity between the WPU/CaCO_3_-60 wt% and WPU/HAp-50 wt%. (C) A heatmap of uncoated fiber cement, WPU/HAp (at 40, 45, and 50 wt%), and WPU/CaCO_3_-60 wt%, highlighting the cooling performance relative to the uncoated substrate.

In the initial trial, CaCO_3_ was replaced with HAp at a loading of 60 wt%; however, the mixture became lumpy and could not be blended into a homogeneous dispersion. This behavior is attributed to the excessively high solid fraction and the higher specific surface area of HAp, which likely promotes stronger interactions with the WPU matrix. Therefore, a reduced filler content was selected. Consequently, the comparative study was conducted at 40 wt%, 45 wt%, and 50 wt%, a range that yields viscosities comparable to conventional paint. The five coating layers at each loading were applied onto glass slides with three repeating pieces to ensure statistical reliability, yielding light transmittance values of 78.6% ± 0.7%, 65.6% ± 0.7%, and 61.7% ± 0.3% as shown in Figure S30. When coated on fiber cement, the corresponding thicknesses were 155 ± 4, 122.00 ± 2, and 163 ± 4 μm, respectively, as presented in Figure S31; WPU/HAp-50 wt% was selected as the optimized concentration due to maximum loading prior to cracking upon formation. The comparative solar reflectance and emissivity of the WPU/CaCO_3_ and WPU/HAp are shown in Figures 7B and S32. With HAp, the reflectance values are significantly increased in accordance with the solar intensity profile within the UV-vis-NIR region up to 48.4%–61.4% (see details in Table S4). Since the refractive index of HAp is 1.63–1.64^61^^,^^62^ similar to those of CaCO_3_ and WPU, the more crystalline structure and the stronger interactions of WPU/HAp lead to a more efficient particle uptake and a better colloidal stability, causing more whiteness appearance and a higher impact on Mie scattering even with less % loading compared to that of CaCO_3_. Moreover, HAp is enrichment of calcium ion and phosphate ion, which are oxygen-rich molecules and show resonance in range of atmospheric transparency window (8–13 μm).^34^ Once adding HAp into WPU matrix, the emissivity graphs show broad band emission along the range of NIR region. It is possible that the multi-active side calcium positive ion of HAp could present van der Waals bonding with negative carboxylate groups of WPU; in addition, negative charges of phosphate in HAp are prone to interact with urethane groups of WPU via hydrogen bonding.^63^^,^^64^^,^^65^ The enhanced bindings from both active sites in WPU/HAp bring about broader and higher average emissivity values up to 96.0% over the atmospheric transparency window range.

The cooling performance of the outdoor system was tested on August 9, 2025, as shown with the full system temperature profiles in Figure S33 and heatmap in Figure 7C. At the peak time of temperature at 13:00 p.m., the WPU/HAp-50 wt% illustrates the system temperature reduction of 9.6°C lower than that of the uncoated fiber cement and 4.3°C lower than that of WPU/CaCO_3_ on the similar substrate. At the same time, IR camera images were captured to compare the surface temperature reductions of the WPU/HAp series on fiber cement, the WPU/CaCO_3_ series on fiber cement, and the WPU/CaCO_3_ series on colored roof tiles along with the uncoated control in Figure S34, consistent with the overall results discussed earlier; WPU/HAp series exhibit the lowest temperatures on both the upper and lower surfaces due to more reflectance and emissivity. Moreover, the radiative power of the WPU/HAp-50 wt% was 321.4 W m^−2^ higher than that of WPU/CaCO_3_-60 wt% (274.7 W m^−2^) at the atmospheric temperature as presented in Figure S35A. Furthermore, the WPU/HAp-50 wt% composite demonstrates a remarkably enhanced P_net_ value, achieving −231.6 W m^−2^ at the atmospheric temperature and −127.8 W m^−2^ at the closed testing-box temperature (Figure S35B). This performance represents an improvement of 218.4 W m^−2^ to 242.7 W m^−2^ over the base WPU and is directly attributed to its significantly enhanced reflectance (61.4%) and high emissivity (96.0%). In comparison, the WPU/CaCO_3_-60 wt% composite also shows beneficial results, exhibiting relative P_net_ from 64.8 W m^−2^ to 79.3 W m^−2^ compared to those of WPU, at the atmosphere and testing box temperatures (Figure S35C).

For the Mohs hardness test, WPU/HAp 50 wt% coated fiber cement exhibits superior surface protection, as only minor scratches were produced even with Mohs pen no. 4. This result suggests that WPU/HAp 50 wt% provides stronger scratch resistance than WPU/CaCO_3_ 60 wt%, corresponding to a hardness range of 3–4 as presented in Figure S36A. For the anti-fouling test, WPU/HAp-50 wt% was evaluated alongside WPU/CaCO_3_-60 wt%. While WPU/HAp-50 wt% demonstrates a comparably clean surface after dust contamination and rain simulation with food coloring solution spreading uniformly across the surface, it exhibits a higher propensity for dye absorption due to higher polarity^66^ caused by HAp content, resulting in a light purple stain remaining on the dried surface (Figure S36B). The surface cleaning results indicate that the coating is expected to remain effective after rainfall, since natural rainwater lacks the artificial dye particles used in the laboratory test. After accelerated weathering test, all conditions including uncoated, WPU, WPU/CaCO_3_, and WPU/HAp exhibit weaker temperature reduction performance (Figure S37).

In conclusion, the semi-transparent passive radiative cooling coating (WPU/CaCO_3_-60 wt%) was achieved by using materials with similar indices of reflection (∼1.65) like WPU/CaCO_3_ and WPU/HAp along with the homogenous particulate sizes and mixing processes, which were achieved via the ball milling. Additionally, the deposition was done through a layer-by-layer paintbrush coating process to allow vertical homogeneity. The WPU/CaCO_3_-60 wt% film on roof tile with a thickness in the range of 60 μm shows 48.2% reflection and exhibits 83.9% emissivity in the atmospheric transparency window through the synergistic effect of WPU/CaCO_3_’s vibrational radiative capacity. In addition, the WPU/CaCO_3_-60 wt% coating can obtain system temperature reduction of ∼5.3°C compared with the uncoated roof tile under sunlight with a relative P_net_ of 64.8 W m^−2^ compared to WPU. Moreover, this semi-transparent coating can be robustly deposited on diverse substrates, such as colorful roof tiles, car stickers, and outdoor housing materials. Meanwhile, WPU/HAp-50 wt% was introduced as a bio-based replacement with much higher average emissivity compared to that of CaCO_3_ in the atmospheric window range. The WPU/HAp-50 wt% at a thickness of ∼150 μm exhibits a superior system temperature reduction of ∼9.6°C compared to that of the uncoated roof tile with 61.4% solar reflectance and 96.0% emissivity in the window region. In summary, this work provides a semi-transparent coating that simultaneously offers heat reduction while still maintaining the visual appearance of the substrate materials.

### Limitations of the study

The temperature reduction performance is dependent on weather conditions.

## Resource availability

### Lead contact

Requests for further information and resources should be directed to and will be fulfilled by the lead contact, Pongsakorn Kanjanaboos (pongsakorn.kan@mahidol.edu).

### Materials availability

This study did not generate new unique reagents.

### Data and code availability

• All datasets generated in this study are available at Mendeley Data under https://data.mendeley.com/datasets/wd434tyckx/1.
• No original code was used.
• Any additional information required to reanalyze the data reported in this paper is available from the lead contact upon request.

## Acknowledgments

This research has received funding support from the National Science, Research and Innovation Fund (NSRF) via the Program Management Unit for Human Resources & Institutional Development, Research and Innovation (PMU-B), and SCG Cement-Building Materials Company Limited (grant no. B42G680036). J.U. has received funding support from the NSRF via the Program Management Unit for Human Resources & Innovational Development, Research and Innovation (grant no. B13F670061). We acknowledge Mahidol University-Frontier Research Facility (MU-FRF) in collaboration with Mr. Chawalit Takoon for FESEM and DLS characterization. We acknowledge Dr. Tanant Waritanant for instrument support. We would like to thank Ko Ko Shin Thant, Chaowaphat Seriwattanachai, Pharit gridtayawong, Varakorn Phiriyasas, and Janjira Jumpimai for insightful discussions and assistance along with data collection.

## Author contributions

J.U., experiments, characterizations, formal analysis, virtualization, and writing – original draft; P.S., experimental assistance, suggestions, and writing – review & editing; W.R., experimental assistance, suggestions, and writing – review & editing; W.B., writing – review & editing; C.S.S. and N.P., reflectance measurement; K.M., reflectance analysis; C.P., calculation of cooling power and heat flux and writing – review & editing; T.K., testing box design; J.K., instruments and characterization support; S.S., instruments and characterization support; W.W., material and characterization support and review & editing; P.K., conceptualization, formal analysis, virtualization, supervision, funding acquisition, and review & editing.

## Declaration of interests

The work is patent pending in Thailand with the application numbers 2601002773 and 2603001601.

## STAR★Methods

### Key resources table

**Table:** 

REAGENT or RESOURCE	SOURCE	IDENTIFIER
**Chemicals, peptides, and recombinant proteins**		

waterborne polyurethan (WPU) premium grade for exterior (gloss finish)	Eco Coat Co. Ltd., Thailand.	N/A
Calcium carbonate 1250 mesh (particle size below 6 μm).	Herosign Marketing Co. Ltd., China.	N/A
Hydroxyapatite	Calcined seashells were used as the source material and further purified through chemical modification and precipitation methods.^33^	The hydroxyapatite was supplied by Assoc. Prof. Worawat Wattanathana from Kasetsart University, Thailand.
Sulfuric acid 98% AR grade	RCI Labscan Co. Ltd., Thailand.	CAS No: 7664-93-9
Sodium hydroxide AR grade	RCI Labscan Co. Ltd., Thailand.	CAS No: 1310-73-2

**Deposited data**		

All datasets for Daytime Radiative Cooling Semi-transparent Roof Tile Coating from Eco-friendly Limestone to Bio-based Hydroxyapatite for Tropical Environment.	This paper	https://data.mendeley.com/datasets/wd434tyckx/1

**Software and algorithms**		

AnalyzIR Venus	FOTRIC USA Inc.	https://www.fotric.com/fotric-analyzir

**Other**		

Fiber cement roof tiles Type: Double-Ribbed Roof Tiles Colors: Natural color, wealthy red, shady blue color, and prosperous blue color. Tile thickness: 0.5 cm. Tile size (W × L): 50 × 150 cm.	Diamond Building Products PCL, Thailand.	N/A
Paulownia wood Thickness: 0.5 cm Size (W × L): 10 × 80 cm.	A local supplier	N/A
Polycarbonate sheet Thickness: 0.5 cm Size (W × L): 122 × 244 cm.	A local supplier	N/A
Aluminum sheet Aluminum grade 1100 Thickness: 2 mm. Size (W × L): 40 × 40 cm.	A local supplier	N/A
Ceramic Color: Glossy natural white Thickness: 0.5 cm Size (W × L): 11 × 11 cm.	The Three Touch Asia Pacific Co., Ltd., Thailand.	N/A

### Method details

#### Preparation of WPU/CaCO_3_ composite and WPU/HAp composite

For the WPU/CaCO_3_ composite, limestone concentrations ranging from 0 to 70 wt% CaCO_3_ were prepared via ball milling method (Planetary Ball Mill model: QM1SP2, Nanjing T-Bota Scietech Instruments & Equipment Co., Ltd, China). A mixed-size zirconia ball was utilized, consisting of 10 mm (*n* = 5), 8 mm (*n* = 11), and 5 mm (*n* = 30) diameters. The commercial pristine WPU solution has 35 wt% of polymer content in aqueous solution. In this work, the CaCO_3_ weight ratio was based on the dried WPU content. Briefly, the CaCO_3_ powder with specific amount and WPU solution were added into a 100 mL ball milling jar and sealed with paraffin film. Subsequently, the ball milling parameters were set to rotate clockwise at 600 rpm for 90 minutes in order to obtain a homogenous WPU/CaCO_3_ composite. Then, the mixtures were stored in plastic container and sealed with paraffin tape to avoid evaporation. Similarly, for the WPU/HAp composite (40-50 wt% Hap) was prepared and stored using the same protocol established for the WPU/CaCO_3_ system.

#### Preparation of WPU/CaCO_3_ specimen and WPU/HAp specimen

Fiber cement roof tile sheet, wood sheet, polycarbonate (PC) sheet, aluminum sheet, and ceramic wall tile were prepared with 8×8 cm^2^ area size and smoothed the surface by sand paper. After that, 0.5 mL WPU/CaCO_3_ composite or WPU/HAp composite were applied on the substrate and manually brushed using a 2.5-inch-wide paintbrush to ensure uniform surface coverage. Each coating layer was dried at room temperature (25 ± 2 °C and humidity 50 ± 5%) without any further treatment. Ultimately, a total of five coating layers were applied with the same process to each substrate. Prior used, all samples were dried at 90°C for 12 hours to remove moisty.

#### Optical and numerical model for cooling performance

Radiative cooling power is a critical parameter for evaluating the cooling performance of their real-world applicability. The effectiveness of radiative cooling materials is fundamentally determined by their ability to manage thermal equilibrium, mitigate solar absorption, and maximize infrared emissivity. The principle of thermal equilibrium dictates that a material absorbs thermal emissions from the atmosphere if its temperature is lower than the surrounding air, which can negate the cooling effect.^4^ Additionally, solar absorption poses a significant challenge. Under typical solar irradiance intensity of the AM1.5 solar spectrum (∼1,000 W·m^-2^), even a minor degree of energy absorption can critically impair the material's cooling capacity.^4^ Therefore, it is crucial to maximize solar reflectance across the ultraviolet, visible, and near-infrared (UV-Vis-NIR) spectrum (0.2–2.5 μm) to minimize solar heat gain. Moreover, the effective cooling requires the material to efficiently dissipate its own thermal energy. This is achieved by maximize thermal emissivity in the atmospheric windows transparency (8–13 μm). This specific atmospheric window allows heat to be radiated directly into the infinite cold reservoir of outer space (∼3K), bypassing atmospheric absorption. This mechanism corresponds to high radiative cooling efficient.^1^^,^^3^ In short summary, the net cooling power (P_net_) is the equilibrium of the radiated power outward (P_rad_) and inward power or the heat absorption including incident solar irradiation absorption (P_sol_), atmospheric thermal radiation absorption (P_atm_), and heat conduction-convection from ambient (P_cond-conv_) as can be described as^4^^,^^6^:

(Equation 1) $$P_{\text{net}}\left(T\right)=P_{\text{rad}}\left(T\right)\;–\;P_{\text{atm}}\left(T_{\text{atm}}\right)\;–\;P_{\text{sol}}\;–\;P_{\left(\text{cond}-\text{conv}\right)}$$

Where the radiated power outward of the emitted material is

(Equation 2) $$P_{\text{rad}}\left(T\right)=A\int d\Omega \;\cos\;\theta \;\int_{0}^{\infty }d\lambda I_{\text{BB}}\left(T,\lambda \right)\varepsilon \left(\lambda ,\theta \right)$$

In this equation, A (m^2^) is the emissivity area, ∫dΩ = 2 $\pi \int_{0}^{\pi /2}d\theta \;\sin\;\theta$ is the integral of the hemisphere (zenith angle),^67^ θ is the zenith angle, I_BB_ (T, λ) is the intensity of a blackbody's radiation wave, dependent on the real-time temperature (T) and wavelength (λ) and ε (λ, θ) is the emissivity of the material, which is a function of both wavelength (λ) and zenith angle (θ).^6^ In addition, the I_BB_ (T, λ) can be expressed into:

(Equation 3) $$I_{\text{BB}}\left(T,\lambda \right)=\frac{2hc^{2}\;}{\lambda^{5}}\;\frac{1}{e^{\frac{hc}{\lambda k_{B}T\;}}-1}$$

Whereby h is the Planck’s constant, k_B_ is Boltzmann constant, and c is the speed of light. Next, the power absorbed by the incident solar irradiation (P_sol_):

(Equation 4) $$P_{\text{sol}}\left(T\right)=A\int_{0}^{\infty }d\lambda \varepsilon \left(\lambda ,\theta_{\text{solar}}\right)\;I_{\text{AM}1.5}\left(\lambda \right)$$

Where I_AM1.5_ (λ) is the solar intensity of the AM 1.5 solar spectrum. θ_solar_ is the angle of incidence solar radiation on the radiative cooling device. The power absorbed by the atmospheric thermal radiation:

(Equation 5) $$P_{\text{atm}}\left(T_{\text{amb}}\right)=A\int d\Omega \;\cos\;\theta A\int_{0}^{\infty }d\lambda I_{\text{BB}}\left(T_{\text{atm}},\lambda \right)\varepsilon \left(\lambda ,\theta \right)\varepsilon_{\text{atm}}\left(\lambda ,\theta \right)$$

(Equation 6) $$\varepsilon_{\text{atm}}\left(\lambda ,\theta \right)=1-{t\left(\lambda \right)}^{1/\cos\;\theta }$$

Where ε_atm_ (λ, θ) is the atmospheric emissivity at a specific wavelength (λ) and zenith angle (θ).

The power lost from heat conduction and convection:

(Equation 7) $$P_{\text{cond}+\text{conv}}\;\left(T,T_{\text{amb}}\right)=Ah_{\text{cc}}\;\left(T_{\text{am}}-T\right)$$

(Equation 8) $$h_{\text{cc}}=h_{\text{conduction}}+h_{\text{convection}}$$

Where h_cc_ is the non-radiative heat transfer coefficient. In low-humidity environments, a typical value for h_cc_ ranges from 0-12 W·m^-2^·k^-1^.^6^ It is important to note that the efficiency of heat transfer and the material's actual operating temperature are highly dependent on multiple factors, such as radiation location, humidity, and other environmental conditions.

#### Measurement of cooling performance

The rooftop testing box was designed to stabilize internal temperatures by reducing the effects of convective heat transfer driven by wind. A prefabricated polystyrene foam box with external dimensions of 16.0 × 16.8 × 13.3 cm^3^ and a wall thickness of 1 cm was modified by removing the bottom section and incorporating a sample-holding compartment. To enhance solar reflectance, the outer surfaces and any areas potentially exposed to sunlight were wrapped with aluminum tape, serving as a reflective layer. After placing the sample into the 8 × 8 cm sample cavity, the upper part of the box was sealed with a transparent LDPE film to create a closed system. A K-type thermocouple probe was calibrated in an ice bath (0 °C) with the standard deviation not more than 0.5 °C along the 59 measurement probes; the probes were inserted into the temperature measurement port located approximately 0.5 cm beneath the sample, and the temperature was recorded every 1 minute using a data logger (Keysight 34972A LXI data acquisition, USA). The solar irradiance intensity (accuracy of ± 5%), humidity (accuracy of ± 2% RH), wind speed and so on were recorded using the weather station (Davis, Vantage Pro2).

#### Characterizations

Particle size analysis of was determined using a laser scattering particle distribution analyzer (Horiba model LA-350). The suspension was prepared as an aqueous dispersion, and the refractive index database was set to 1.6, which closely corresponds to that of calcium carbonate. Simulated solar transmittance testing was conducted using an LED solar simulator (VeraSol LSH-7520, Oriel Instruments, USA) with an LED controller (LSS-7120 Solar Simulator). The solar intensity was calibrated to 1.000 kW m^-2^ using a silicon diode (Hamamatsu S1133) before each measurement. The surface morphology and cross-sectional features of the roof tiles were characterized by a field emission scanning electron microscope (FESEM; JEOL, JSM 7610 FPlus, Japan). To ensure high-quality imaging and prevent electron beam charging, samples were first sputter-coated with a fine, electrically conductive layer of platinum for 120 seconds prior to analysis. The surface texture of the coating layer was analyzed using an optical microscope (Olympus, BX53MRF–S, 0E41082) and an optical topography 3D profiler (Keyence, VK-X3000). Solar reflection was investigated using UV-vis-NIR spectroscopy (Shimadzu model UV-3600) in the range of 0.3 to 2.5 μm with a spectral resolution of 1 nm. Infrared emissivity between 2.5 to 25 μm was measured by Fourier transform infrared (FTIR) microscopy (Nicolet Apex, Thermo Scientific) with an integrating sphere (downward integrat IR™) accessory. Infrared thermal images were captured using a thermal camera (Fortric model 348A, China). The color gradients were adjusted using AnalyzIR software to analyze the surface temperature distributions within the selective emissivity regions. The temperature data was recorded in real-time using a datalogger (Keysight 34972A LXI data acquisition, USA). The samples were tested with QUV accelerated weathering tester model QUV/spray. The CIE values were measured by spectroradiometer (Instrument systems, CAS 140D) with accessory (Instrument system model EOP-146) and xenon lamp. The hardness of coating films was determined by Mohs’ hardness testing kit (China).

### Quantification and statistical analysis

The quantification samples were performed in triplicate (*n* = 3). Data are presented as mean or mean ± standard deviation (SD). Statistical analysis of data was performed using Excel (Microsoft) and Origin (OriginLab) as present in Figures 1C, 3A, 3B, 5C–5F, 6A, 6B, 7C, S15, S16, S18, S19, S22, S23, S30, S33, and S37.
