# Supplementary material for: Daytime radiative cooling semi-transparent roof tile coating from eco-friendly limestone to bio-based hydroxyapatite for tropical environment
Source: iScience. 2026 Apr 27;29(10):115893. doi: 10.1016/j.isci.2026.115893 (PMC13631666; doi:10.1016/j.isci.2026.115893)
Supplement: Supplementary file 1 [file mmc1.pdf]

iScience, Volume *29*

Supplemental information

Daytime radiative cooling semi-transparent roof tile coating from eco-friendly limestone to bio-based hydroxyapatite for tropical environment

Jittrakorn Udomsin, Patawee Sakata, Worawut Rueangsawang, Wachara Benchaphanthawee, Chayani S. Sarumaha, Kullachate Muangnapoh, Chattrarat Ponghiransmith, Taweesak Kaewmanee, Napan Phuphathanaphong, Jakrapong Kaewkhao, Supakij Suttiruengwong, Worawat Wattanathana, and Pongsakorn Kanjanaboos

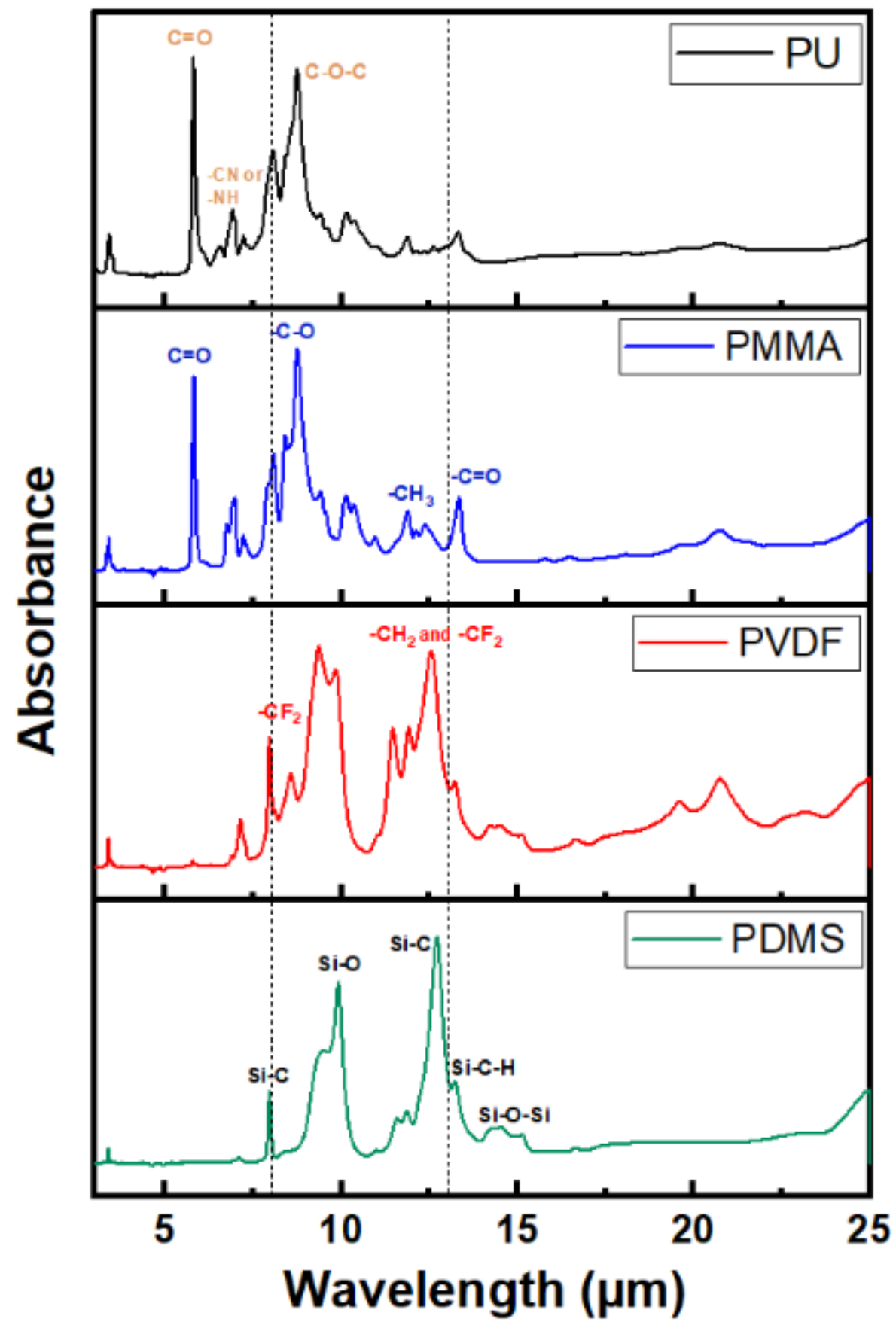

Figure S1: The FT-IR absorption of various kinds of polymer. Polydimethylsiloxane (PDMS), poly (vinylidene fluoride) (PVDF), and poly (methyl methacrylate) (PMMA) have been widely reported in radiative cooling application.[1] PDMS shows outstanding absorption in range 8-13 micron, however, dust absorption and peeling are key problems. Moreover, PDMS fabrication requires organic solvent such as hexane which may affect the nervous system. The transparent PVA has issues the durability and usage of organic solvent. PVDF is another alternative that has been reported in the literature.[2] However, PVDF tends to have lower emissivity than those of PDMS and PMMA. In addition, PVDF is relatively expensive, less readily available, and requires organic solvents for dissolution. Consequently, it may be unsuitable for residential roof-coating applications, which demand high durability, large-scale applicability, and low cost. PMMA was an interesting candidate, which is transparent coating, available on market, and can be mass produced with low price and good durability. However, numerous publications have already explored the material. Alternatively, waterborne polyurethane (WPU) has similar advantages. In addition, WPU present -NH- functional group along with carboxylate group,[3–5] which allows more bonding and better adhesivity on various substrates.

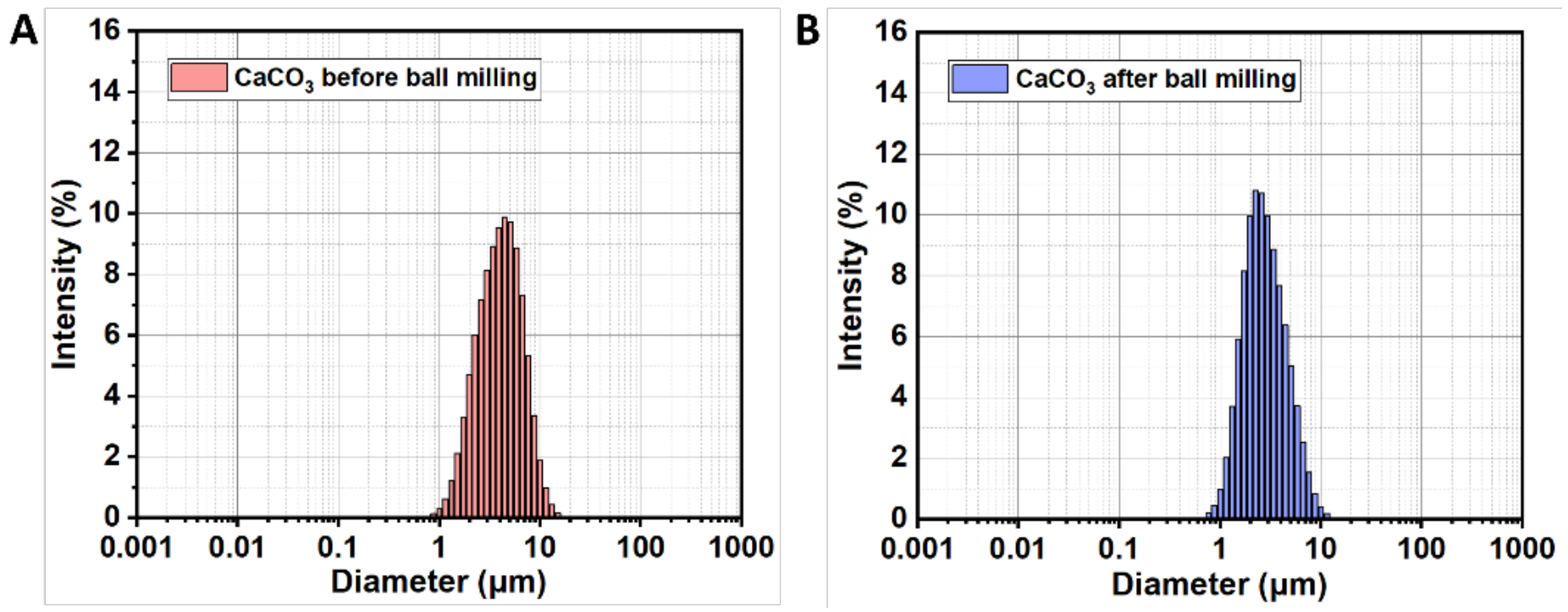

Figure S2: Particle size distribution. A.) $CaCO_3$ before ball milling. B.) $CaCO_3$ after ball milling. The specific parameters in the ball milling process are mainly rotation speed (rpm), grinding time (h), ball size (mm), and ball number.[6] As the higher value of parameters above, the particle size in wet grinding process tends to be smaller from micro to nano size. The efficient scattering is a function of particle size by the following equation: $\lambda = D/k$ where λ is wavelength (m), D is particle diameter.[7] When particle size is smaller than visible wavelength and particle/matrix refractive indices are similar, more transparency can be expected due to less scattering. In addition, low volume fraction and more homogeneous mixing by ball milling reduces scattering while increases transparency.

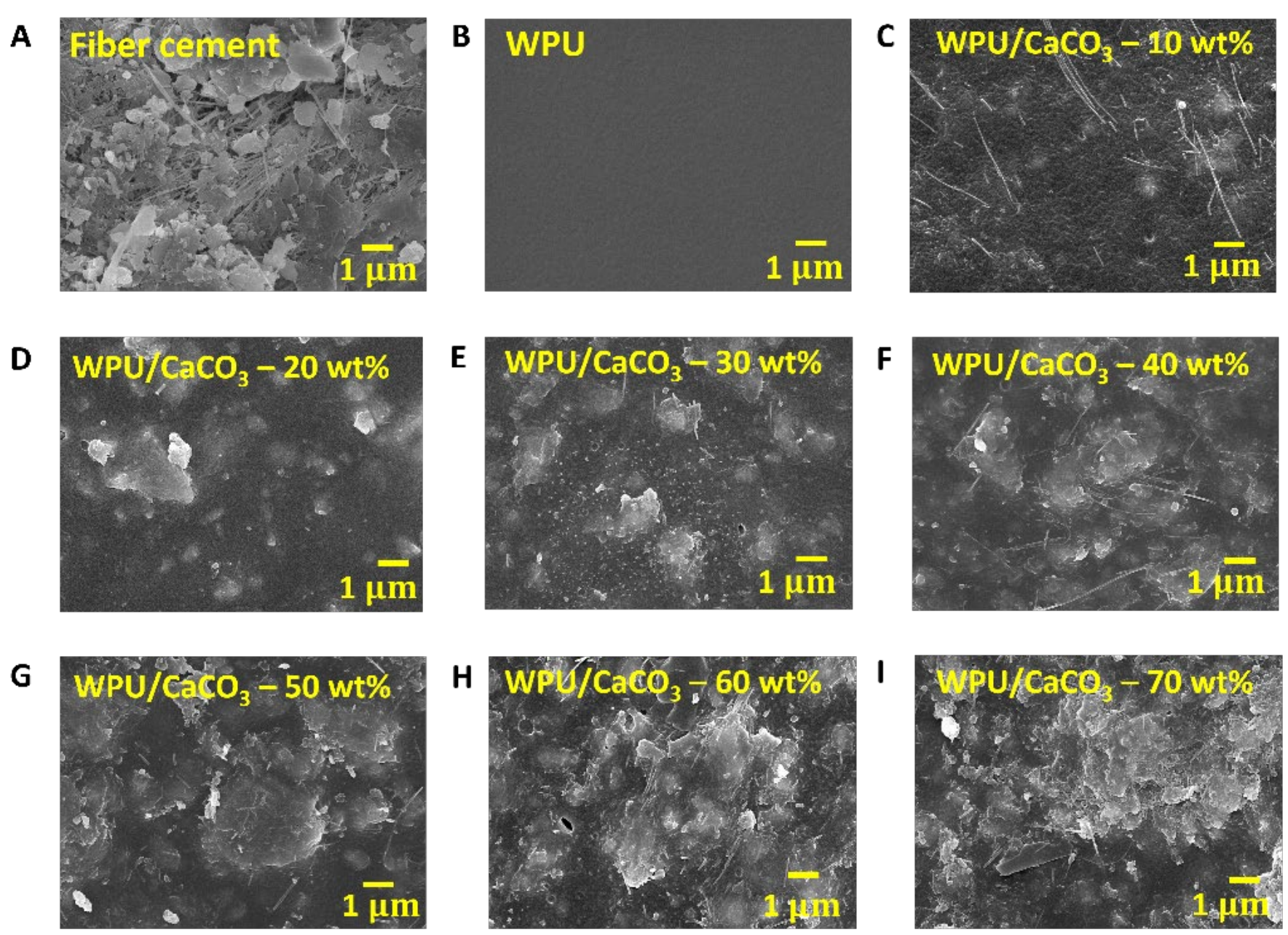

Figure S3: FESEM micrographs of fiber cement and WPU/$CaCO_3$ coatings.

A.) Fiber cement. B.) WPU. C-I.) WPU/$CaCO_3$ – 10, 20, 30, 40, 50, 60, and 70 wt%, respectively (scale bars represent 1 µm).

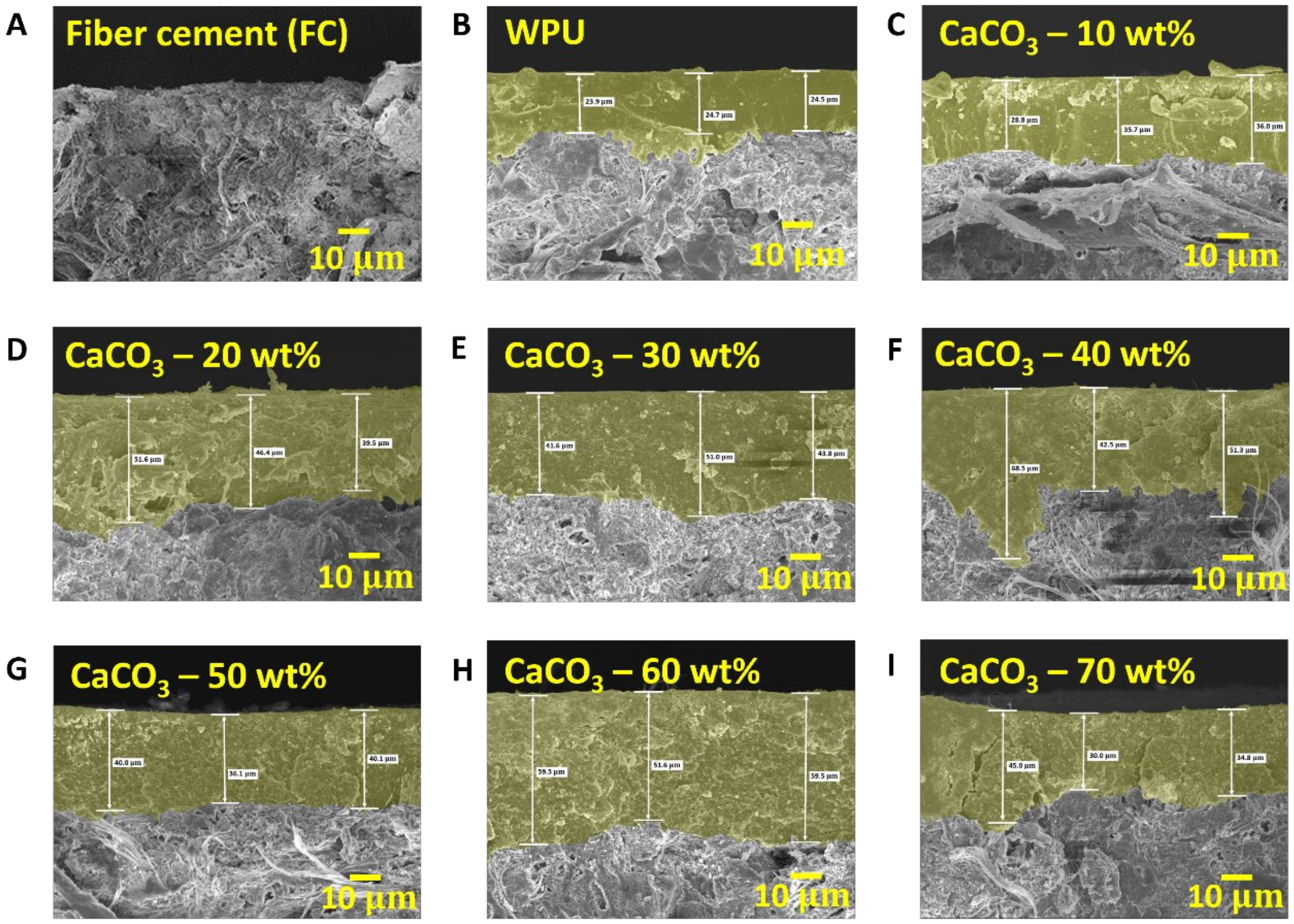

Figure S4: **Cross-section FESEM micrographs of fiber cement and WPU/$CaCO_3$ coatings.**

A.) Fiber cement. B.) WPU. C–I.) WPU/$CaCO_3$ – 10, 20, 30, 40, 50, 60, and 70 wt%, respectively (scale bars represent 10 µm).

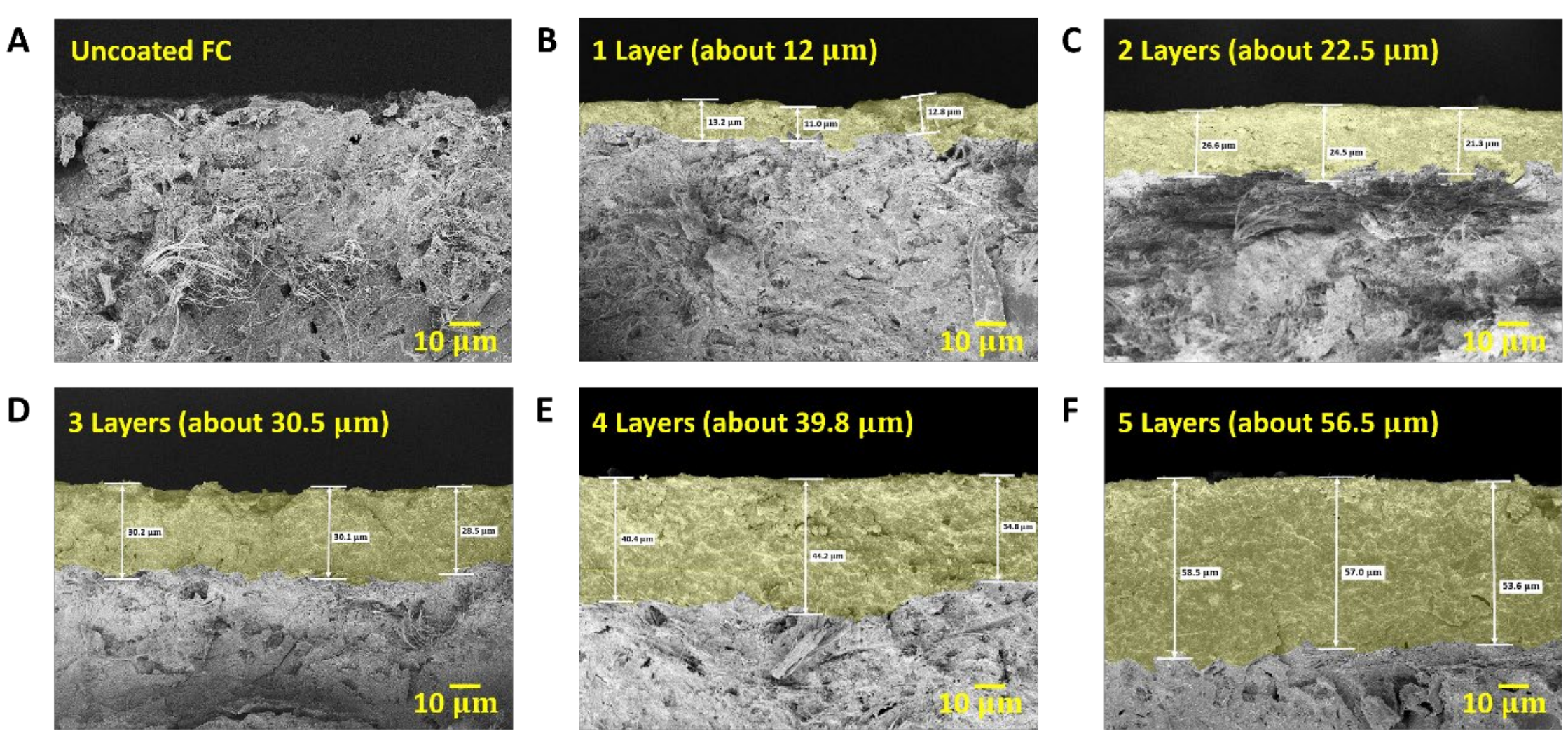

Figure S5: **Cross-section FESEM micrographs of fiber cement and WPU/CaCO3 coating with different number of layers.** A.) Bare roof tile. B–F.) WPU/$CaCO_3$–60 wt% coated roof tiles with 1, 2, 3, 4, and 5 layers, respectively (scale bars represent 10 µm).

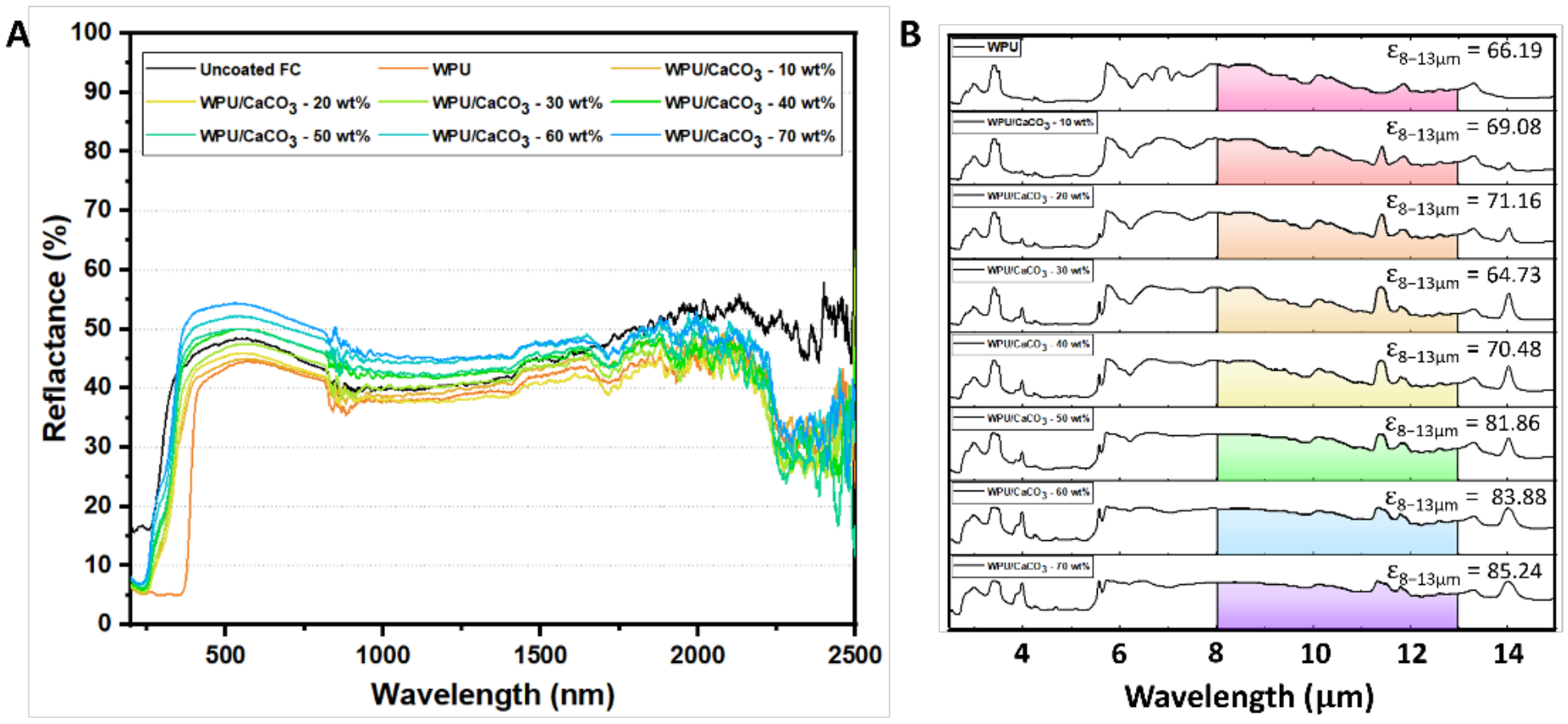

Figure S6: Optical properties. A.) The reflectance spectra of uncoated FC, WPU, and WPU/$CaCO_3$–10 - 70 wt%, respectively. B.) The FTIR spectra and emissivity values of WPU, and WPU/$CaCO_3$–10 - 70 wt%, respectively.

Table S1: Summary of light transmittance, reflectance and emissivity values.

$CaCO_3$ Concentration	Light transmittance	Reflectance				Emissivity (8-13 μm)
		Solar (300-2500 nm)	UV (300-400 nm)	Visible (400-700 nm)	IR (700-2500 nm)	
Uncoated substrate	90.0 ± 0.1 %	44.7	43.6	47.5	42.9	-
0 wt% (WPU)	90.0 ± 0.1 %	40.3	13.5	43.3	39.6	66.2
10 wt%	88.6 ± 0.1 %	41.9	34.7	44.1	40.8	69.1
20 wt%	85.2 ± 0.1 %	41.7	35.5	45.1	39.7	71.2
30 wt%	83.6 ± 0.3 %	43.5	38.1	46.6	41.7	64.7
40 wt%	81.2 ± 0.2 %	45.8	41.8	49.2	43.7	70.5
50 wt%	75.8 ± 0.1 %	46.0	42.9	49.3	44.0	81.9
60 wt%	71.4 ± 0.4 %	48.2	45.3	51.4	46.2	83.9
70 wt%	58.3 ± 1.4 %	49.6	47.8	53.6	47.1	85.2
QUV uncoated FC	-	43.1	38.4	44.2	42.6	-
QUV WPU	-	33.9	25.2	34.7	43.7	-
QUV WPU/$CaCO_3$–60 wt%	-	41.6	35.8	43.7	40.5	-

It is note that the light transmittance was measured on the glass substrate to ensure the semi-transparent of the WPU/$CaCO_3$ coating. While, the reflectance was measured on the fiber cement substrate for tile cooling application. However, the fiber cement absorbs infrared spectrum and unable to analyze the emissivity of the WPU/$CaCO_3$ coated fiber cement. Then, the WPU/$CaCO_3$ were coated on aluminum substrate.

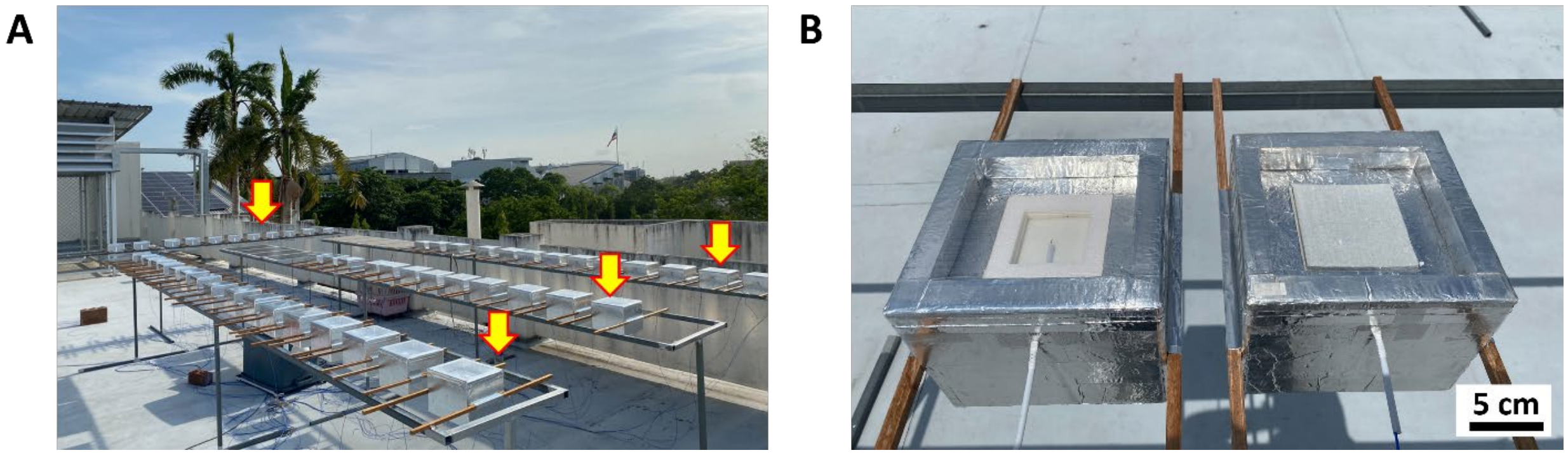

Figure S7: Photographs of the experimental setup for real-time thermal monitoring on the rooftop.

A.) The rooftop experimental setup. B.) Testing boxes with and without sample (scale bar represents 5 cm).

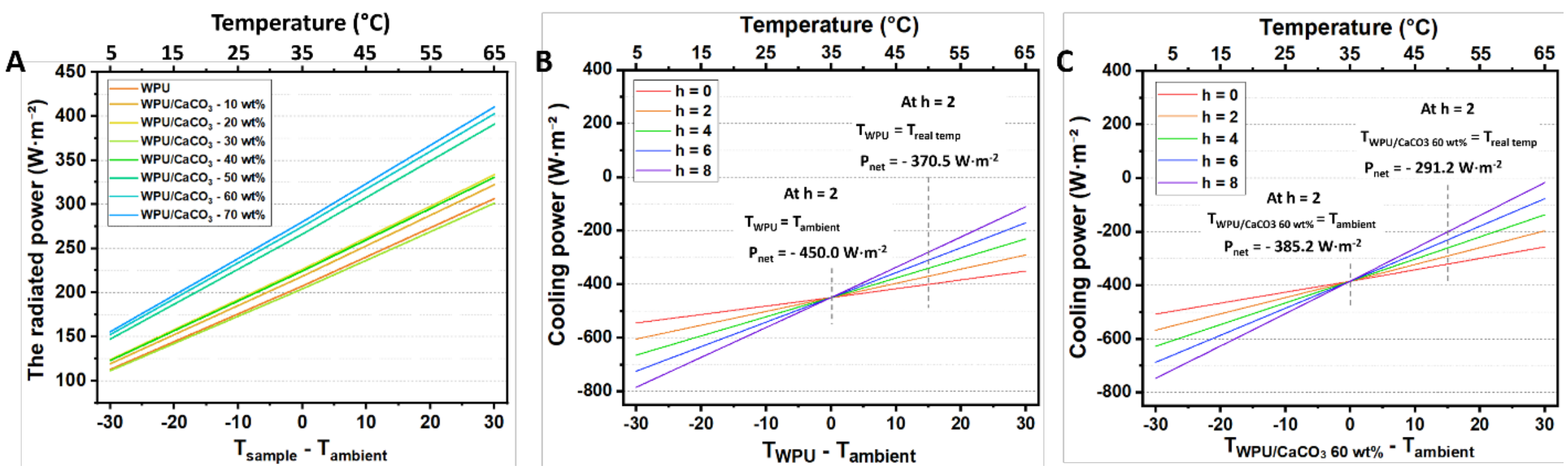

Figure S8: Calculation of heat flux and cooling power of WPU and WPU/$CaCO_3$ composites.

A.) Calculated heat flux of WPU and WPU/$CaCO_3$–10-70 wt%. B.) Cooling power as a function of temperature difference of T_{WPU} - $T_{ambient}$. C.) Cooling power as a function of temperature difference of $T_{WPU/CaCO3,\ 60\ wt\%}$ - $T_{ambient}$.

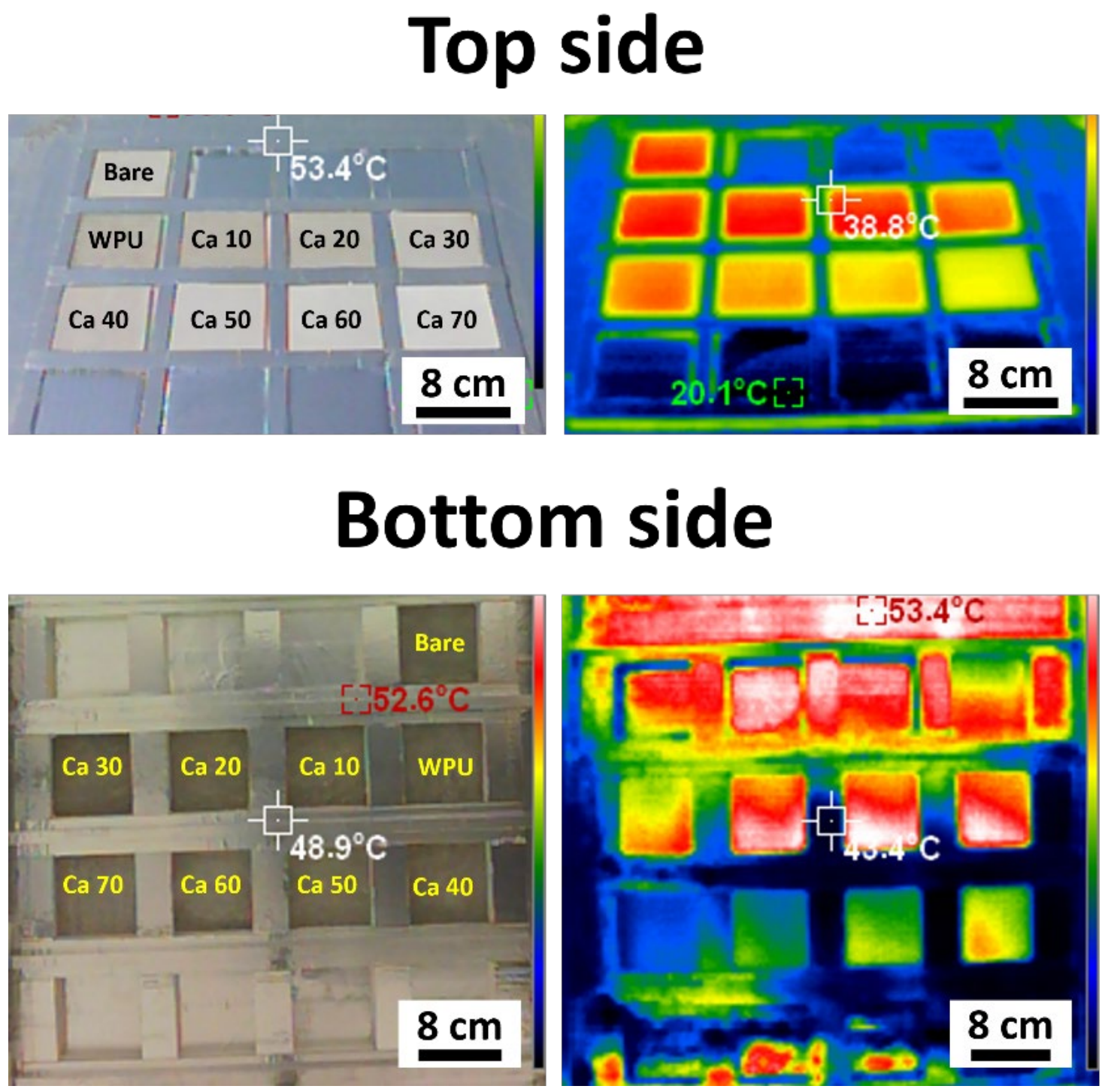

Figure S9: **Infrared thermography images of each sample from both top and bottom sides (scale bars represent 8 cm).**

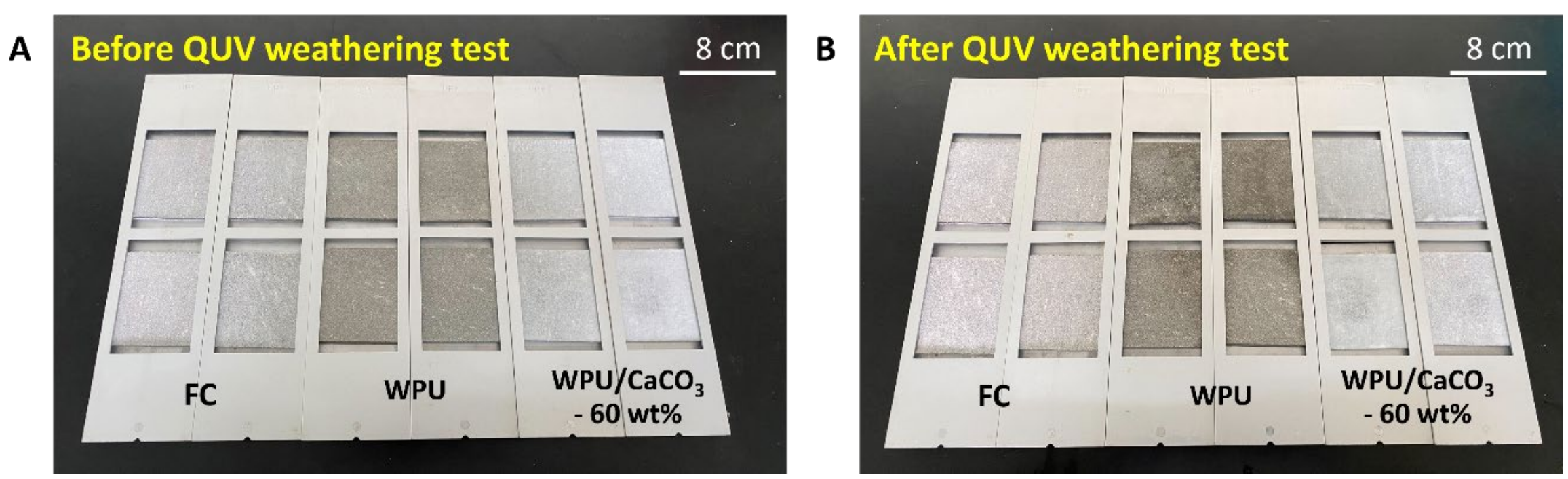

Figure S10: **The photograph of coated roof tiles before and after QUV weathering test.**

A.) Before QUV weathering test. B.) After QUV weathering test. Scale bars represent 8 cm.

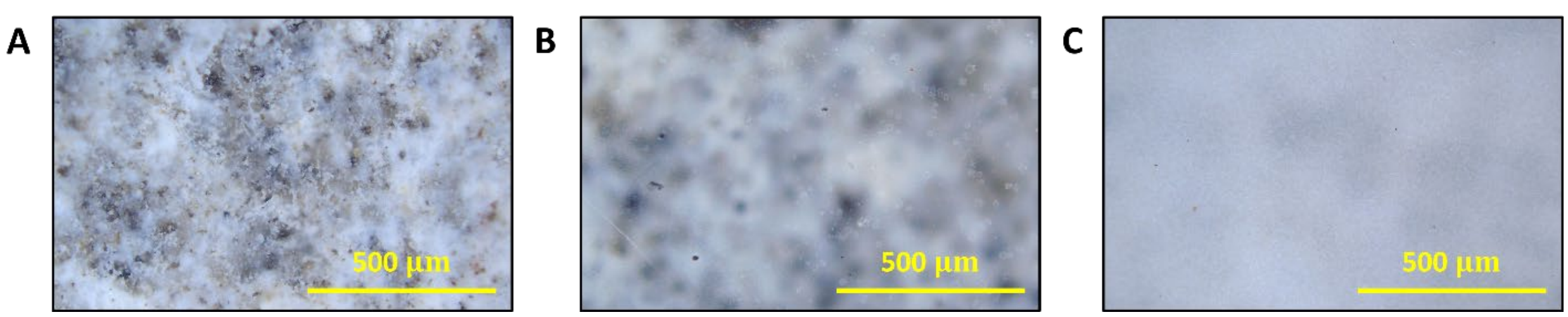

Figure S11: Optical microscopic images after QUV weathering test. A.) QUV uncoated fiber cement. B.) QUV WPU. C.) QUV WPU/$CaCO_3$–60 wt%. Scale bars represent 500 µm.

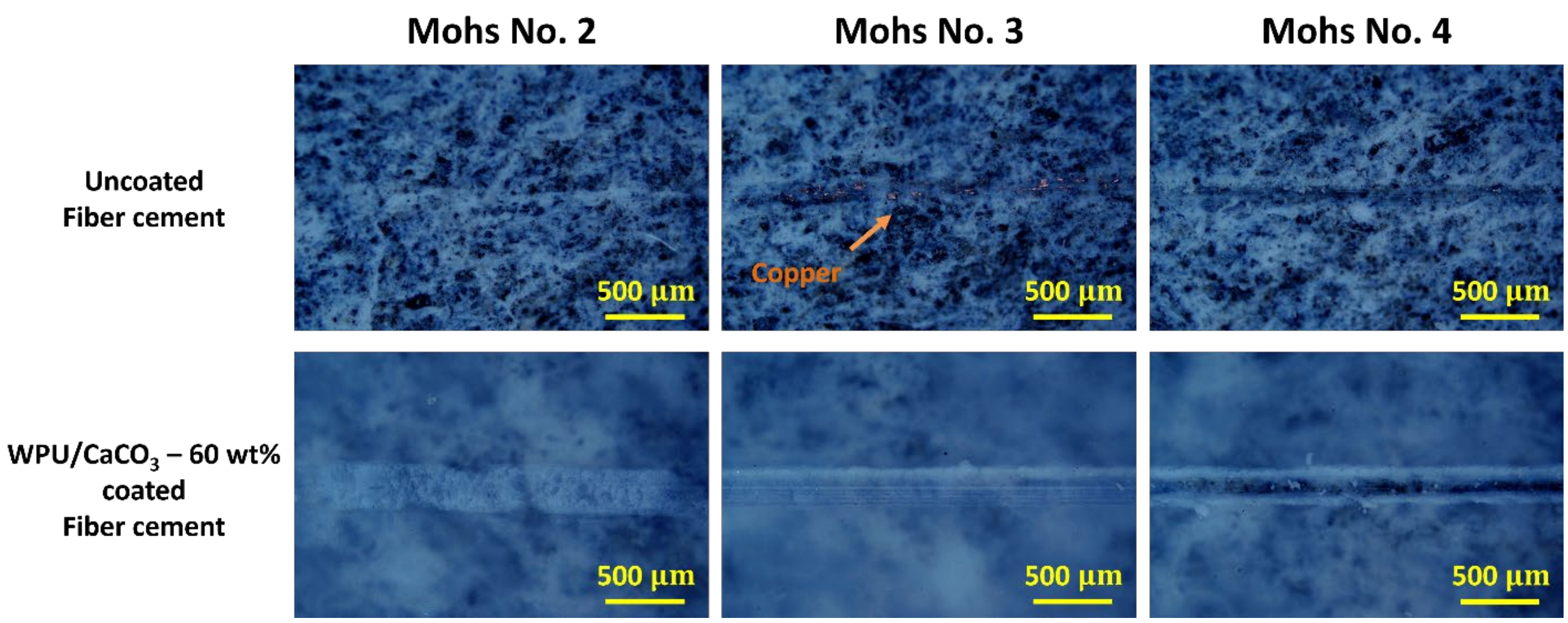

Figure S12: Comparison of Mohs hardness test results between uncoated fiber cement and WPU/$CaCO_3$–60 wt% coated fiber cement (scale bars represent 500 μm).

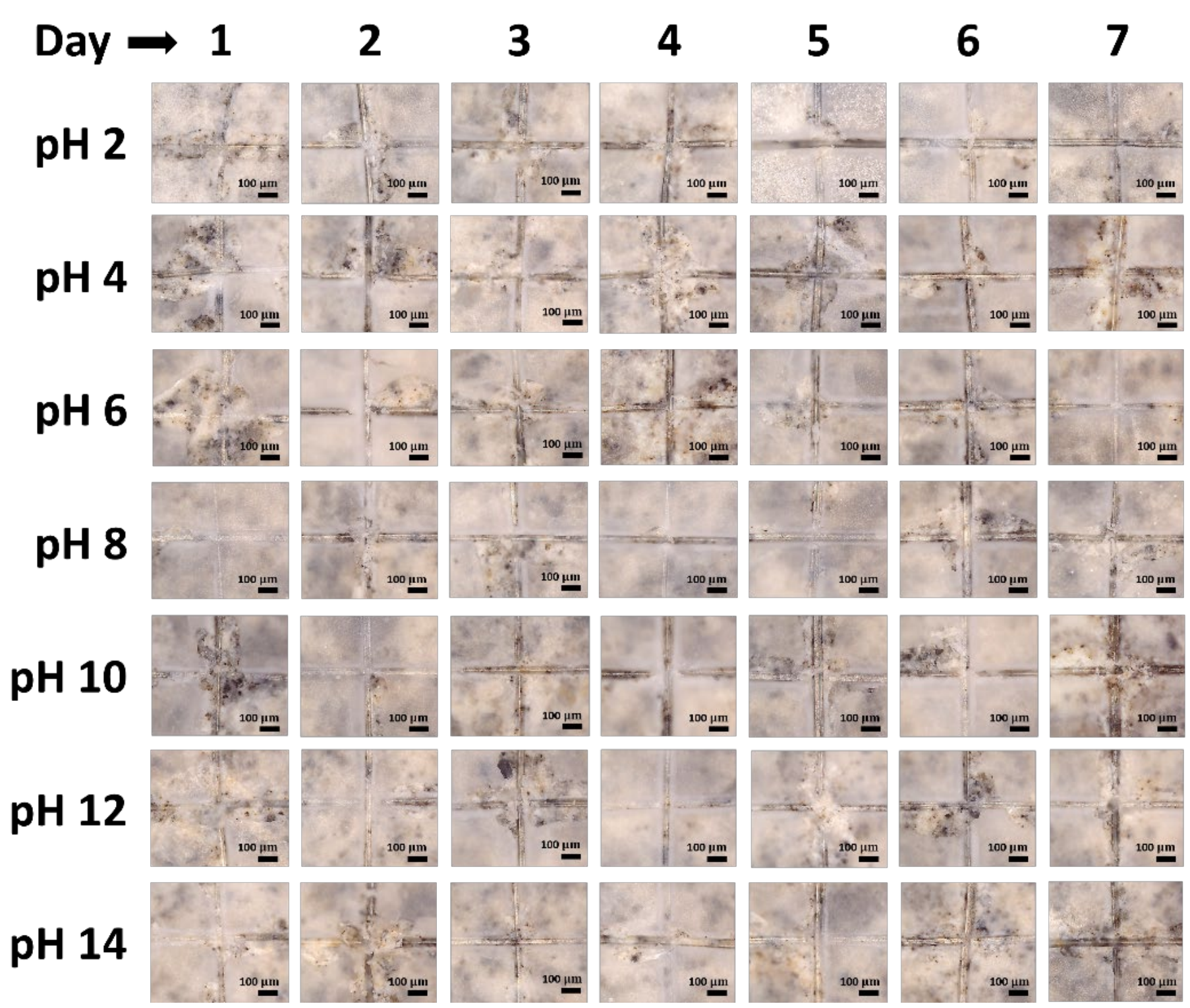

Figure S13: Optical microscopic images of cross-cut tests and subsequent peeling using 3M pineapple tape of WPU/$CaCO_3$–60 wt% coated roof tiles immersed in solutions with pH 2-14 for 7 days (scale bars represent 100 μm).

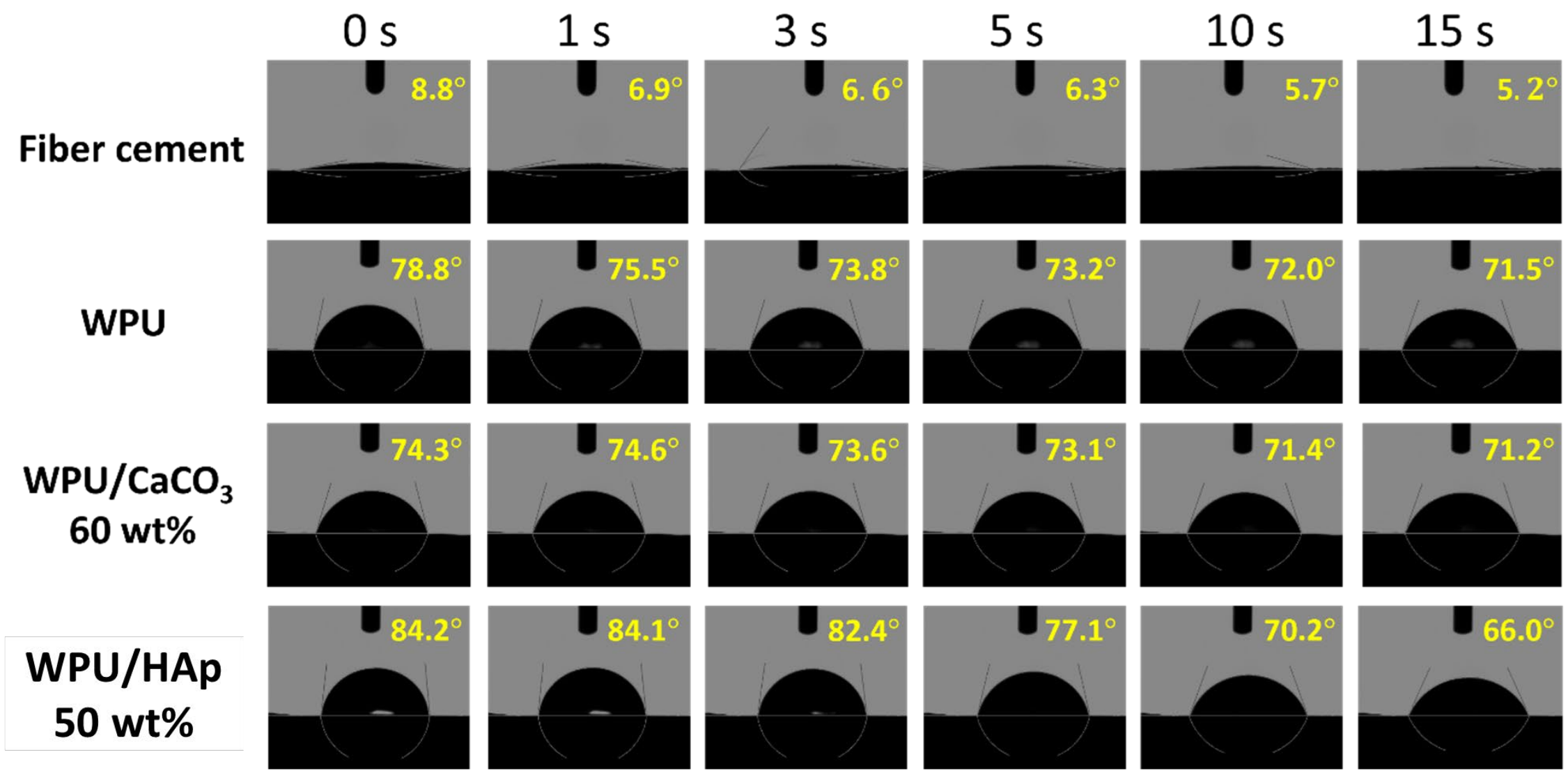

Figure S14: The photograph of water contact angle (WCA) on different coating by fiber cement, WPU, WPU/$CaCO_3$-60 wt%, and WPU/$CaCO_3$-50 wt% at different time.

Table S2: CIE value of uncoated and coated color roof tile.

No.	Samples	x	y
1	Uncoated grey (FC)	0.335	0.337
2	Coated grey	0.334	0.335
3	Red	0.417	0.339
4	Coated red	0.374	0.327
5	Green	0.248	0.316
6	Coated green	0.290	0.315
7	Blue	0.254	0.231
8	Coated blue	0.269	0.258

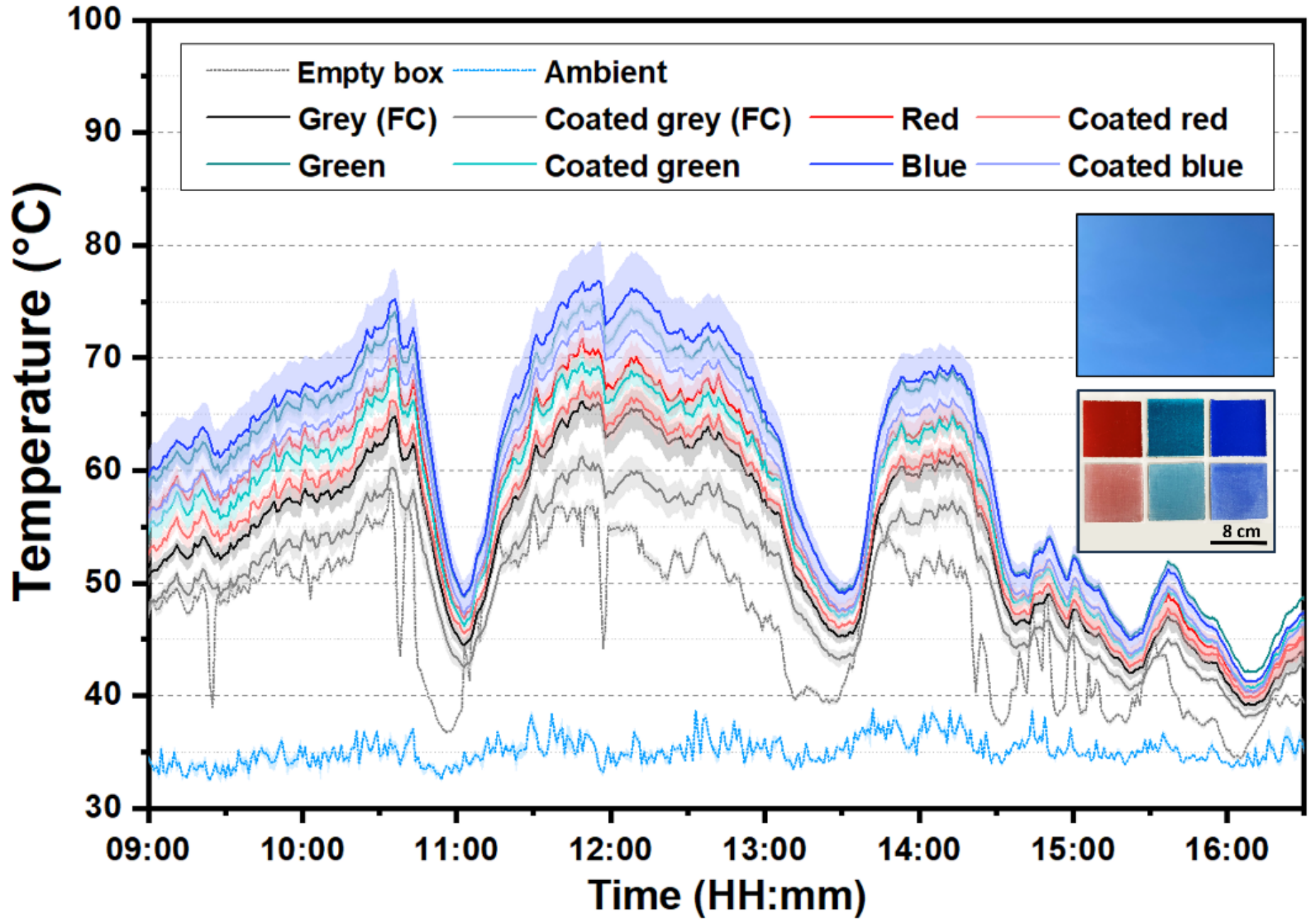

Figure S15: Temperature profiles of uncoated and WPU/$CaCO_3$–60 wt% coated roof tiles in grey, red, green, and blue. Three replicates of the 8×8 cm^2-sized samples were tested to obtain the statistical data (scale bar represents 8 cm). The results are presented as the mean (central line) with standard deviation (S.D.) (shaded contour region).

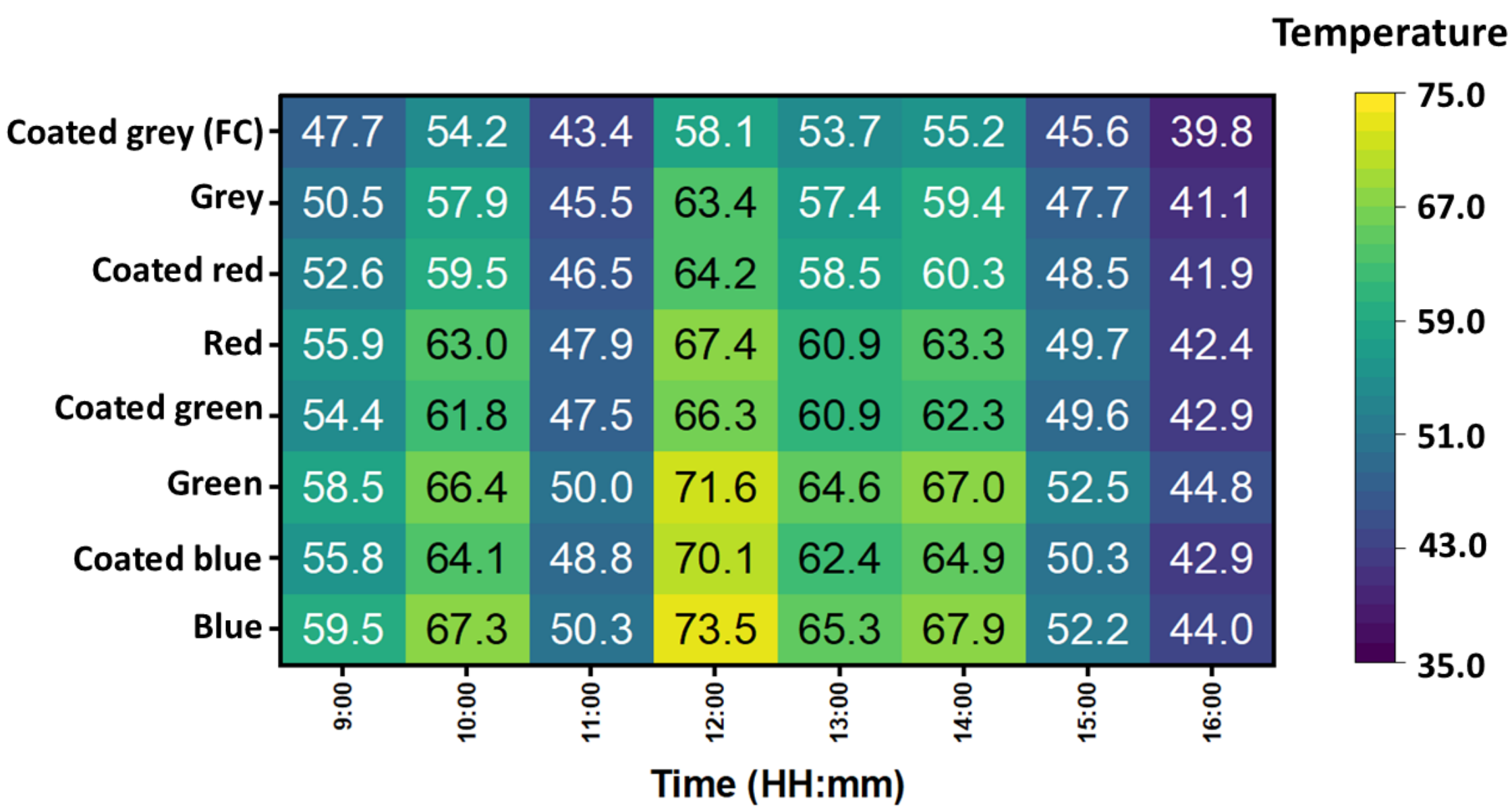

Figure S16: Heat map analysis comparing the temperature of WPU/$CaCO_3$–60 wt% coated and uncoated roof tiles recorded from 9:00 to 16:00.

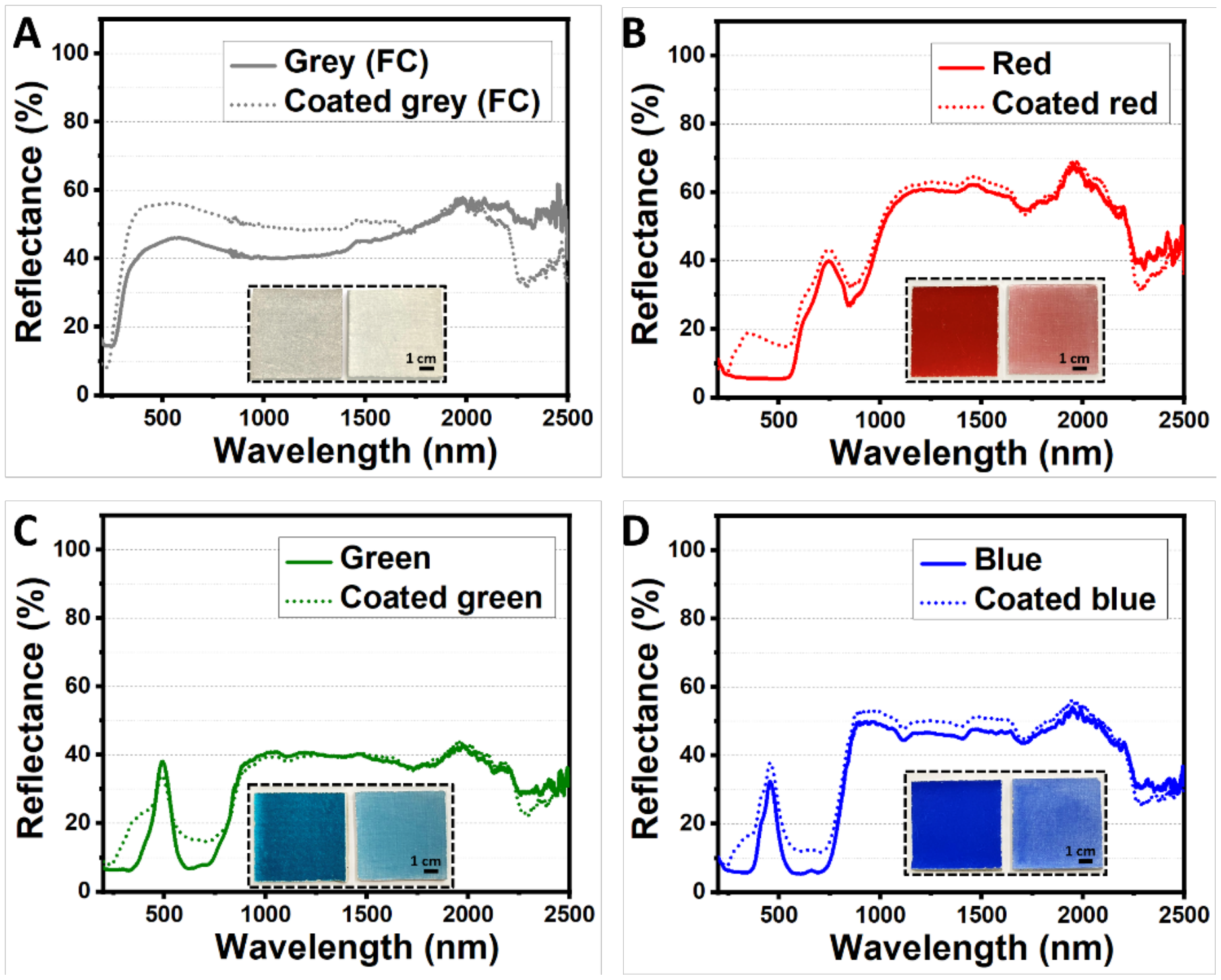

Figure S17: Comparison of reflectance spectra of uncoated and WPU/$CaCO_3$–60 wt% coated roof tiles. A.) Grey. B.) Red. C.) Green. D.) Blue. Scale bars represent 1 cm.

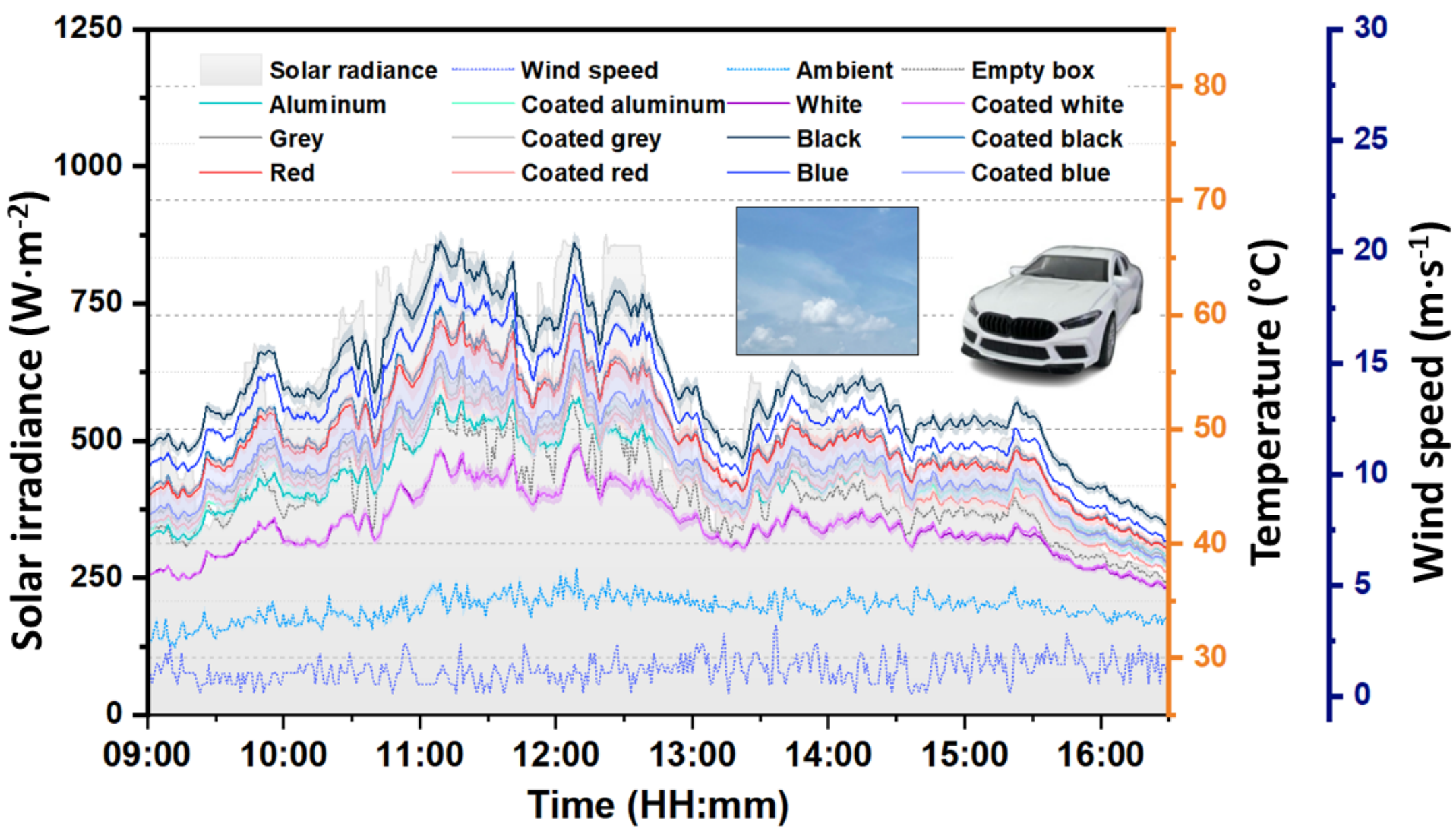

Figure S18: The full day temperature profiles uncoated and coated WPU/$CaCO_3$–60 wt% automotive stickers that applied on 2-mm-thick aluminum sheet. Three replicates of the 8×8 cm^2-sized samples were tested to obtain the statistical data. The results are presented as the mean (central line) with standard deviation (S.D.) (shaded contour region).

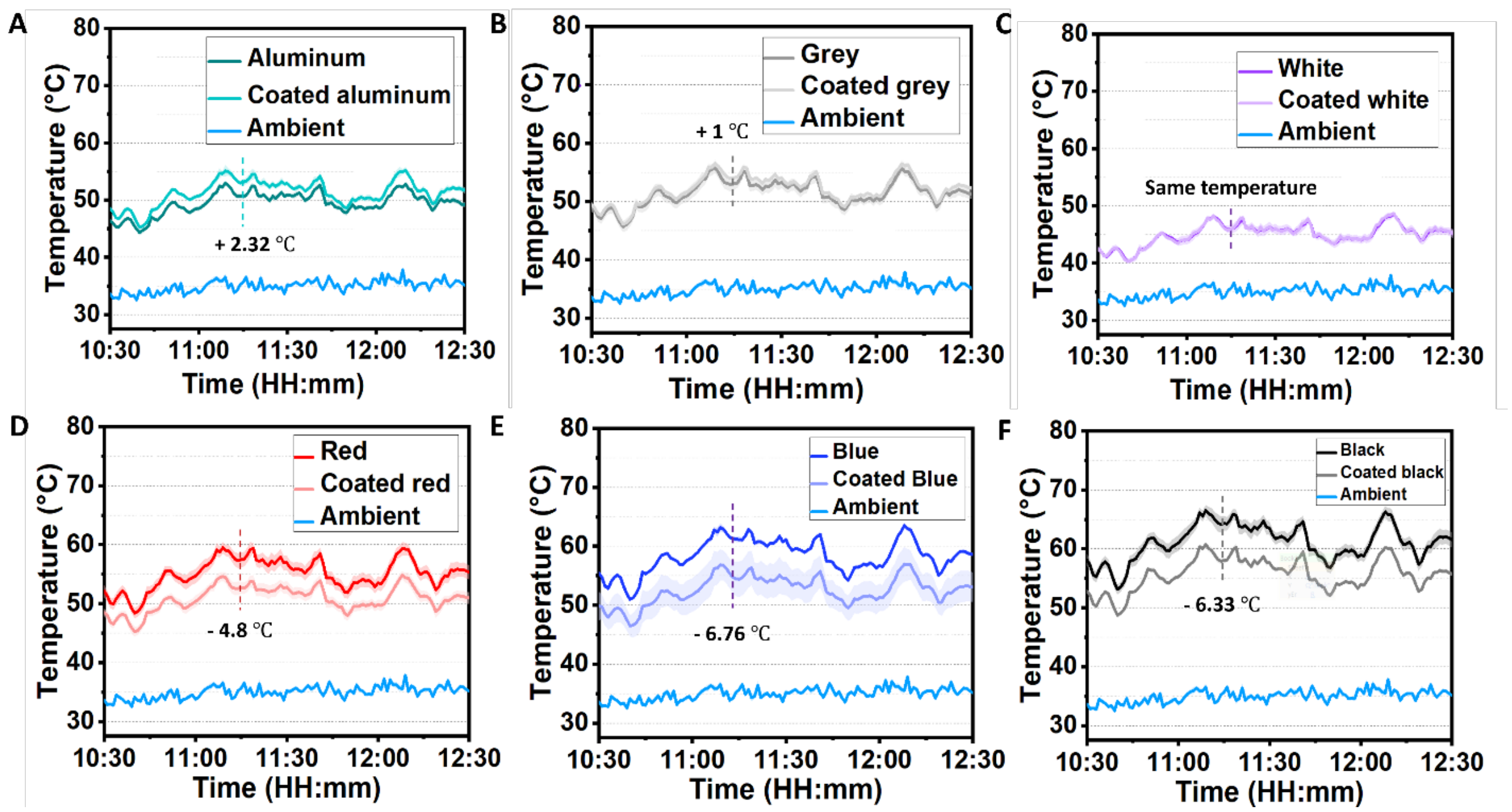

Figure S19: **Maximum temperature profiles and corresponding temperature differences between WPU/$CaCO_3$-60 wt% coated and uncoated samples.** A.) Aluminum (without stickers). B–F.) Automotive stickers in grey, white, red, blue, and black, respectively.

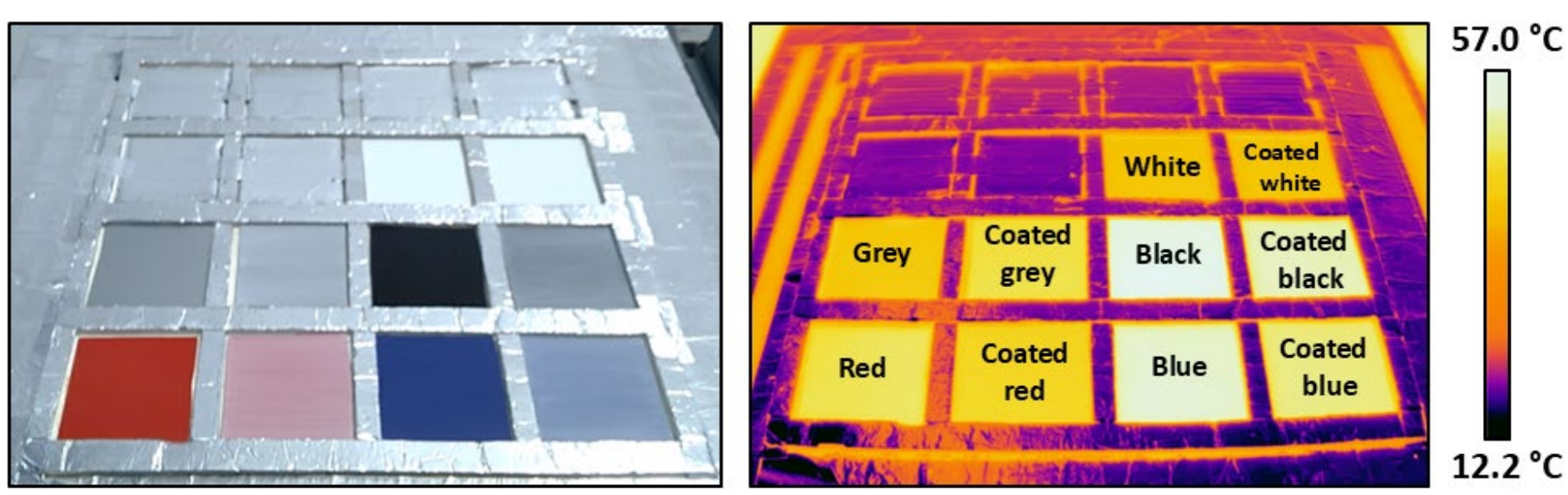

Figure S20: Photograph and infrared thermography image of WPU/$CaCO_3$-60 wt% coated and uncoated automotive stickers of 8×8 cm^2-sized samples.

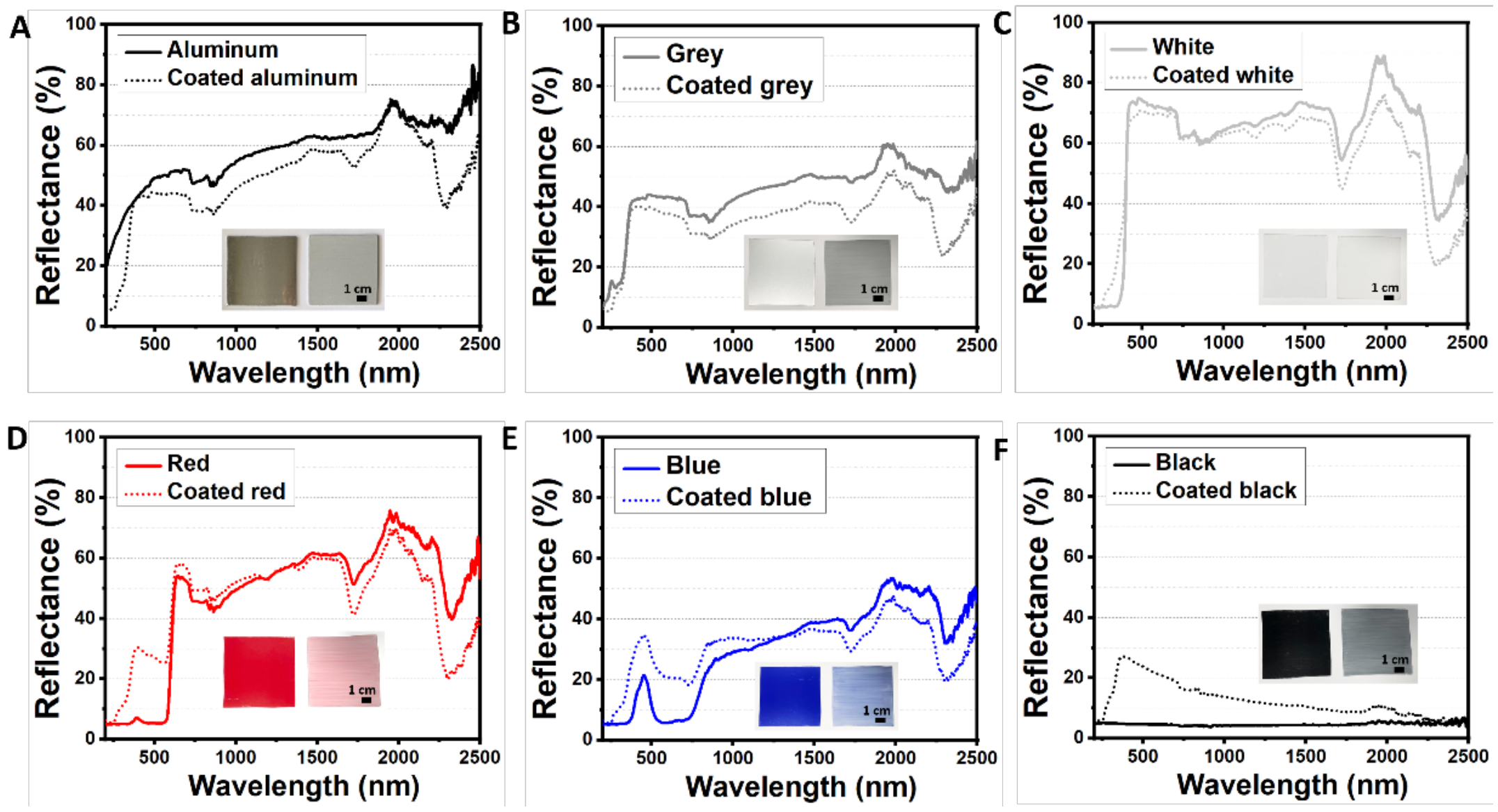

Figure S21: **Comparison of reflectance spectra of WPU/$CaCO_3$-60 wt% coated and uncoated automotive stickers.** A.) aluminum (without stickers). B–F.) Automotive stickers in grey, white, red, blue, and black, respectively (scale bars represent 1 cm).

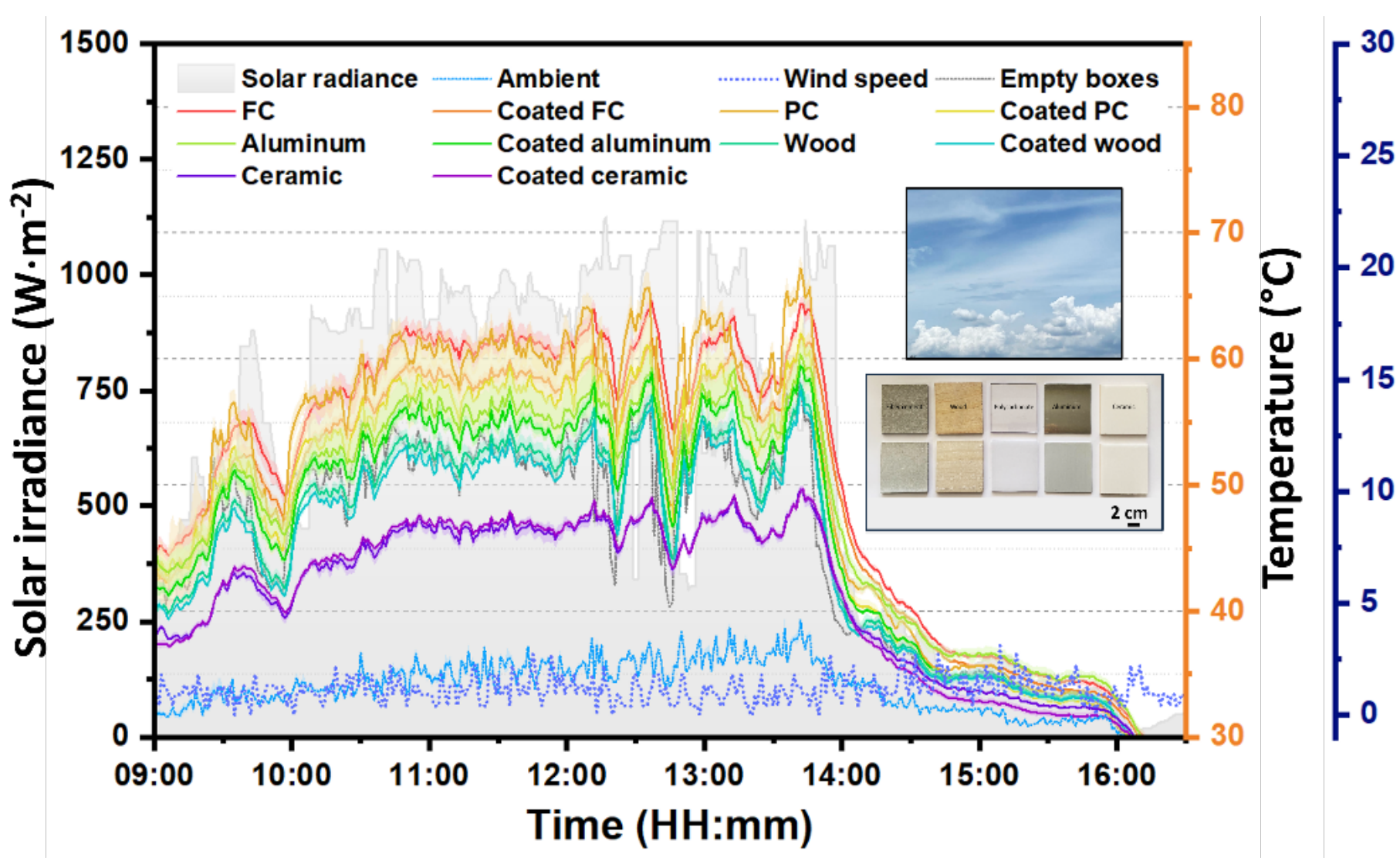

Figure S22: The full day temperature profiles of WPU/$CaCO_3$–60 wt% coated and uncoated building material substrates. Three replicates of the 8×8 cm^2-sized samples were tested to obtain the statistical data. The results are presented as the mean (central line) with standard deviation (S.D.) (shaded contour region). Scale bar represents 2 cm.

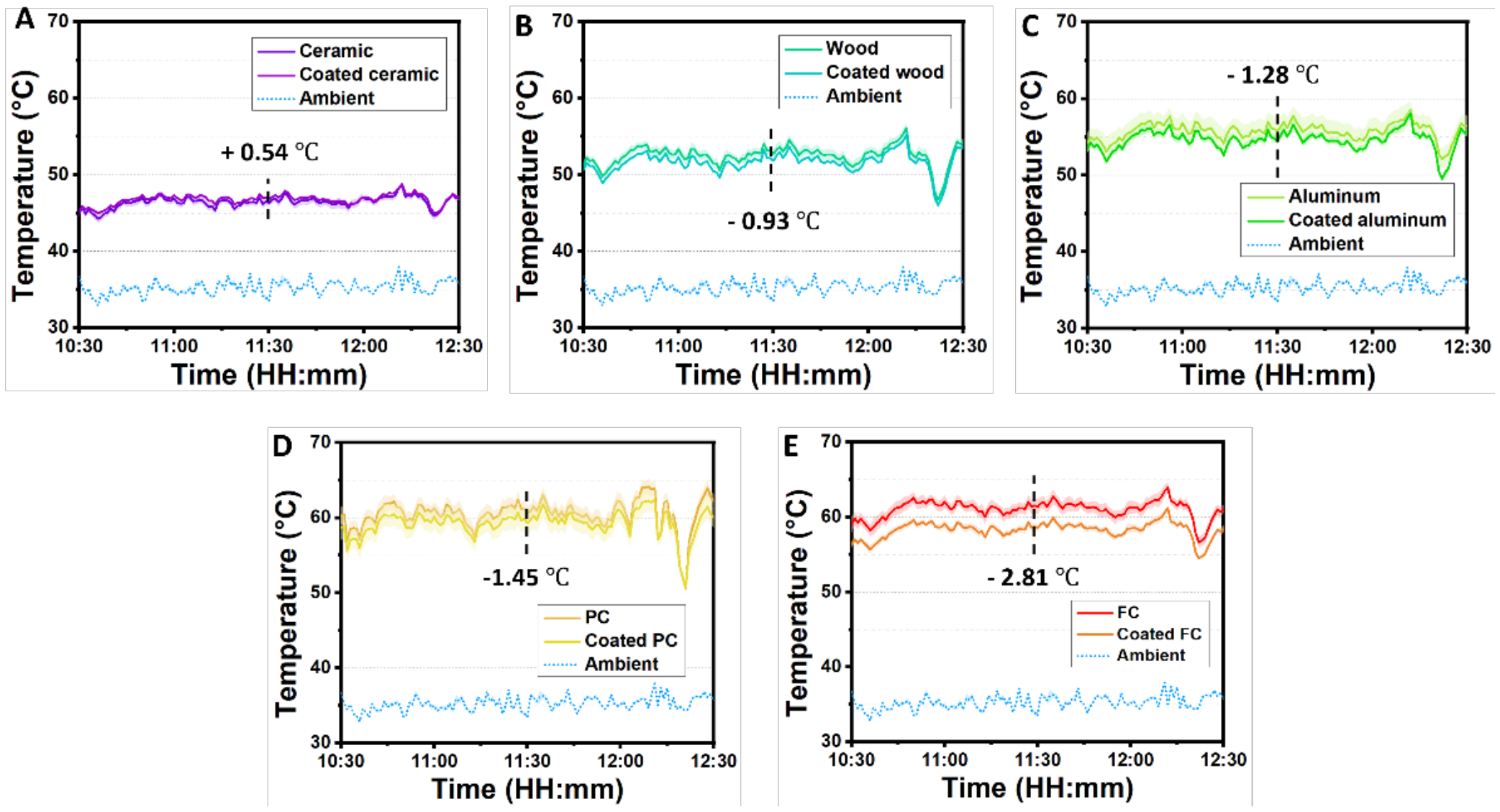

Figure S23: Maximum temperature profiles and corresponding temperature differences of WPU/$CaCO_3$–60 wt% coated and uncoated outdoor housing materials. A.) Ceramic. B.) Wood. C.) Aluminum. D.) PC. E.) FC.

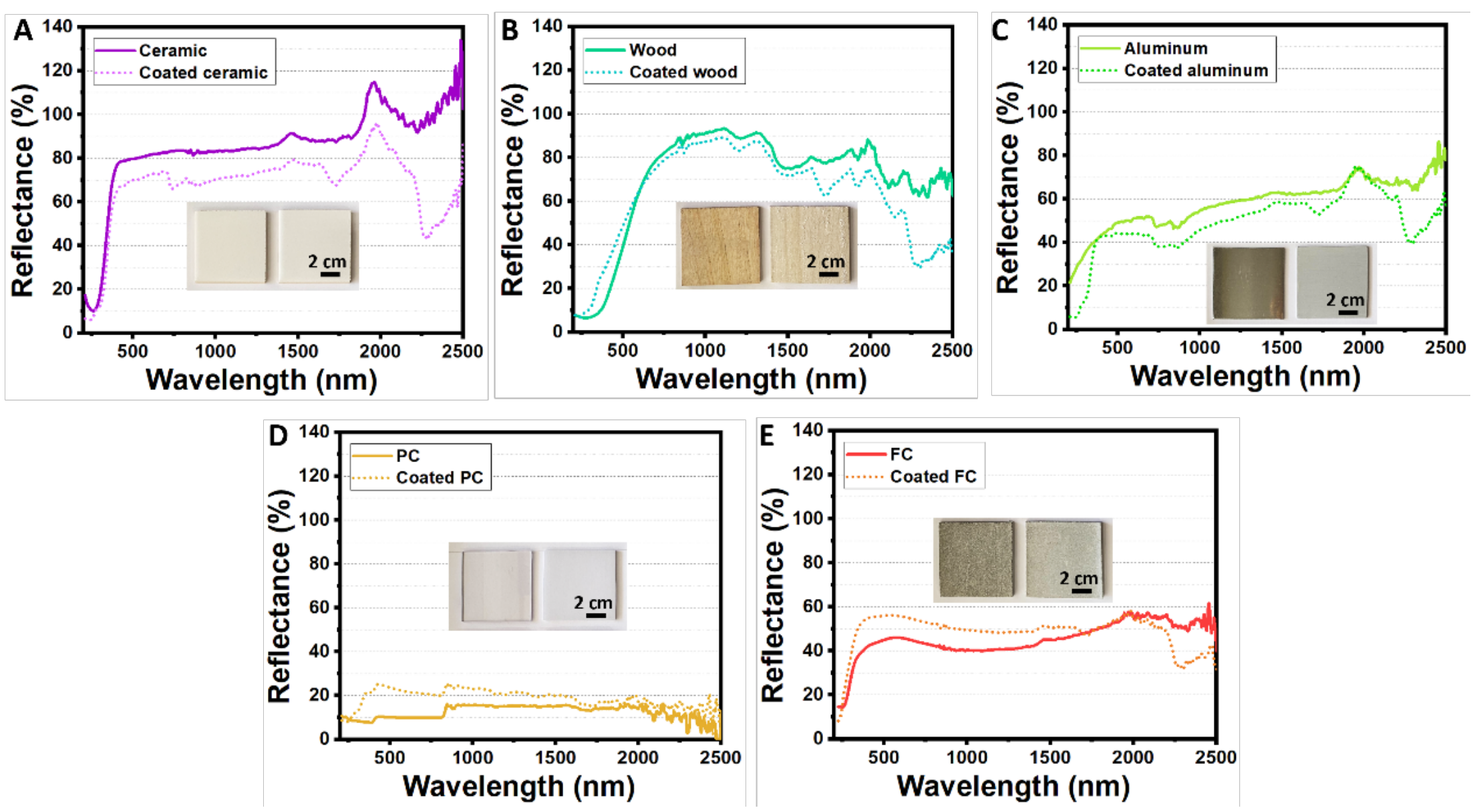

Figure S24: Comparison of reflectance spectra of WPU/$CaCO_3$-60 wt% coated and uncoated outdoor housing materials. A.) Ceramic. B.) Wood. C.) Aluminum. D.) PC. E.) FC. Scale bars represent 2 cm.

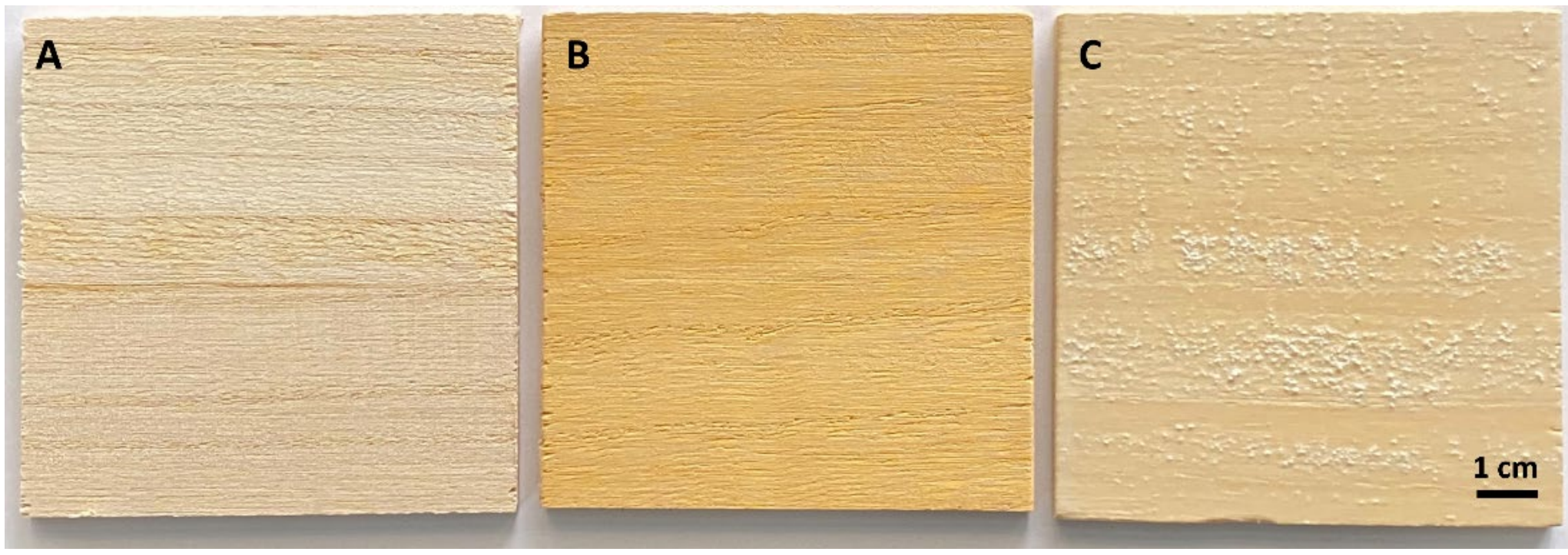

Figure S25: Photographs of wood after sun exposure. A.) Original wood color. B.) Yellowish wood surface after sun exposure for a week. C.) WPU/$CaCO_3$–60 wt% coated wood after sun exposure for a week. Scale bar represents 1 cm.

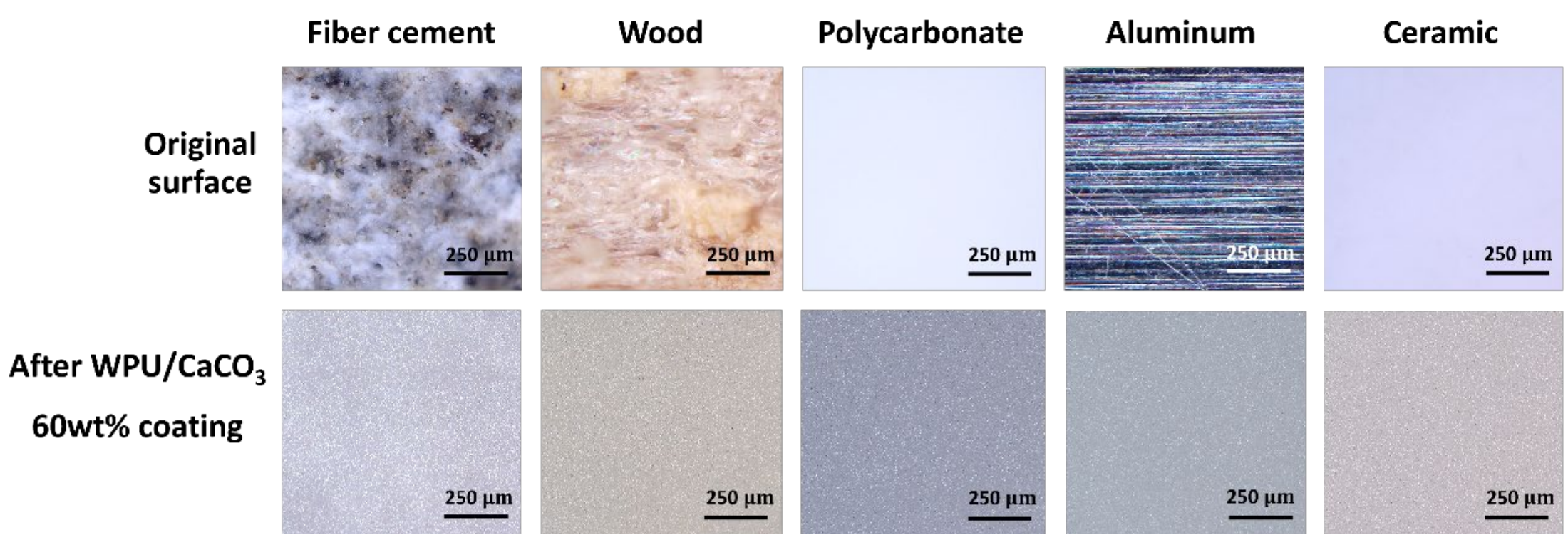

Figure S26: Optical microscopic images comparing original and WPU/$CaCO_3$ - 60 wt% coated on different surfaces (scale bars represent 250 µm).

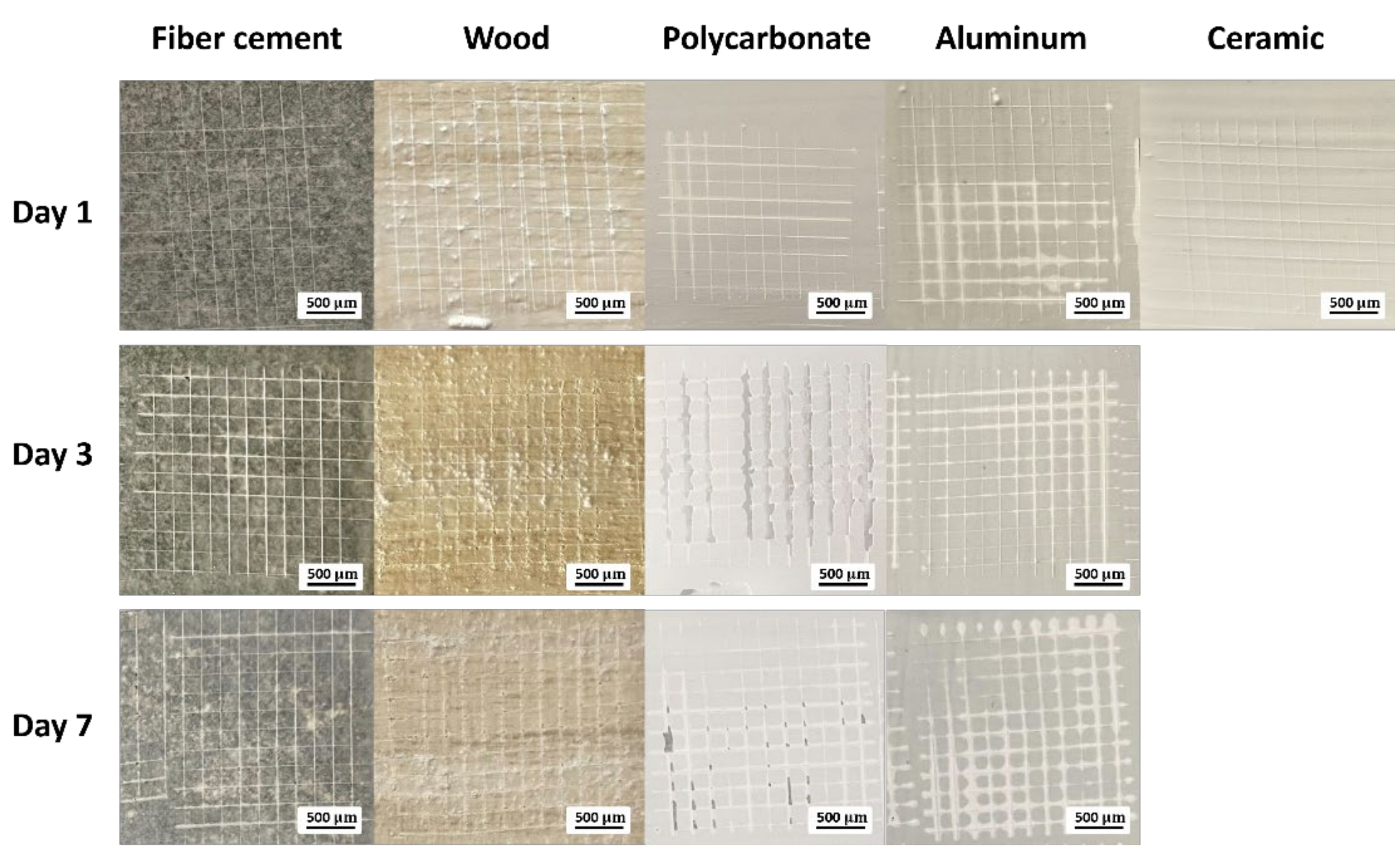

Figure S27: Photographs of cross-cut tests of substrates after immersion in water for 1, 3, and 7 days (scale bars represent 500 µm).

Table S3: Summary of reflectance value for coated and uncoated substrates.

No.	Samples	Solar spectrum 300-2500 nm (%)	UV 300-700 nm (%)	Vis 400-700 nm (%)	NIR 700-2500 nm (%)
1	FC: Grey	43.7	39.5	45.0	43.1
2	FC: Grey/ WPU/$CaCO_3$ - 60 wt%	52.3	52.4	55.5	50.2
3	FC: Red	32.4	5.7	13.4	46.4
4	FC: Red/ WPU/$CaCO_3$ - 60 wt%	37.6	18.3	21.9	49.1
5	FC: Green	25.4	9.3	17.3	31.6
6	FC: Green/ WPU/$CaCO_3$ - 60 wt%	27.6	21.6	21.6	31.9
7	FC: Blue	27.3	8.7	12.3	38.2
8	FC: Blue/ WPU/$CaCO_3$ - 60 wt%	32.3	18.4	19.2	41.8
9	AT: White	66.2	15.2	72.2	64.8
10	AT: White/ WPU/$CaCO_3$ - 60 wt%	63.8	31.3	69.2	61.7
11	AT: Grey	42.7	36.2	43.1	42.8
12	AT: Grey/ WPU/$CaCO_3$ - 60 wt%	36.1	33.8	38.3	34.7
13	AT: Red	38.2	6.1	21.5	51.1
14	AT: Red/ WPU/$CaCO_3$ - 60 wt%	45.1	24.9	37.1	51.5
15	AT: Blue	19.8	7.8	9.7	27.2
16	AT: Blue/ WPU/$CaCO_3$ - 60 wt%	28.1	22.9	24.8	30.6
17	AT: Black	4.4	4.8	4.6	4.3
18	AT: Black/ WPU/$CaCO_3$ - 60 wt%	17.1	25.4	22.2	13.2
19	Ceramic	82.9	62.5	80.6	85.5
20	Ceramic/ WPU/$CaCO_3$ - 60 wt%	70.2	50.8	70.9	70.7
21	Aluminum	52.5	41.1	49.4	55.1
22	Aluminum/ WPU/$CaCO_3$ - 60 wt%	45.1	37.6	43.7	46.5
23	Wood	70.2	10.9	52.3	85.1
24	Wood/ WPU/$CaCO_3$ - 60 wt%	69.2	25.3	56.9	79.7
25	PC	12.0	7.9	10.0	13.5
26	PC/ WPU/$CaCO_3$ - 60 wt%	21.6	20.7	22.7	21.0

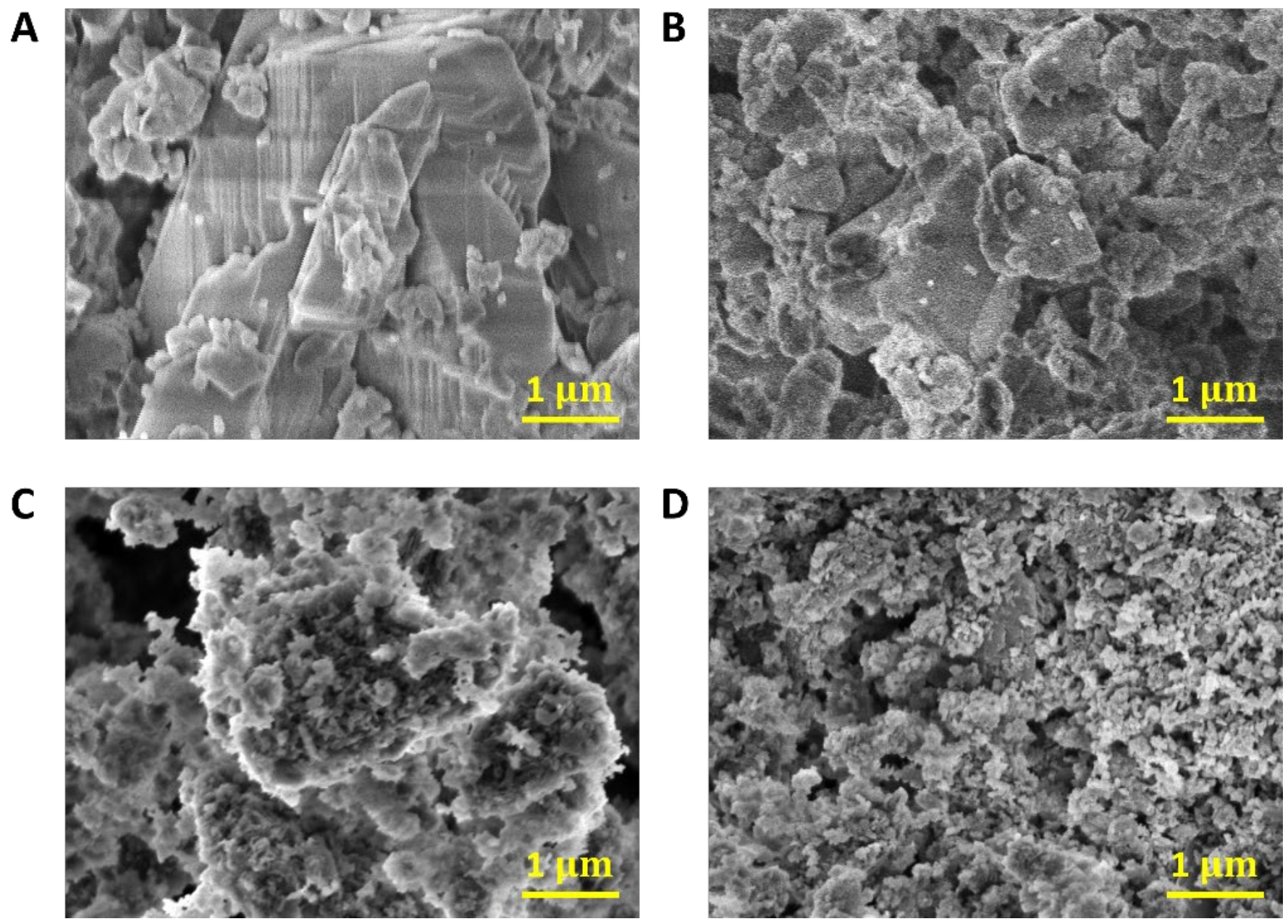

Figure S28: FESEM images of particles. A.) $CaCO_3$ particles before ball milling. B.) $CaCO_3$ particles after ball milling. C.) HAp particles before ball milling. D.) HAp particles after ball milling (scale bars represent 1 μm).

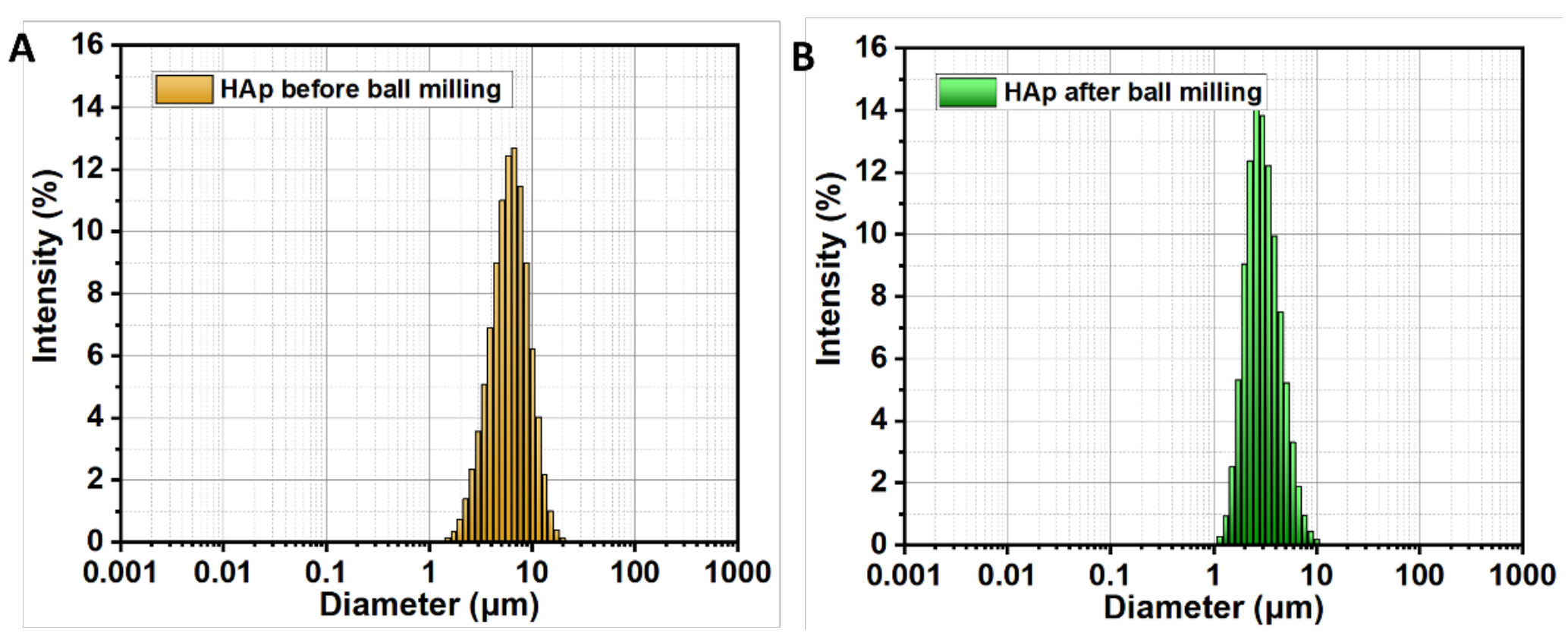

Figure S29: Particle size distribution. A.) HAp before ball milling. B.) HAp after ball milling.

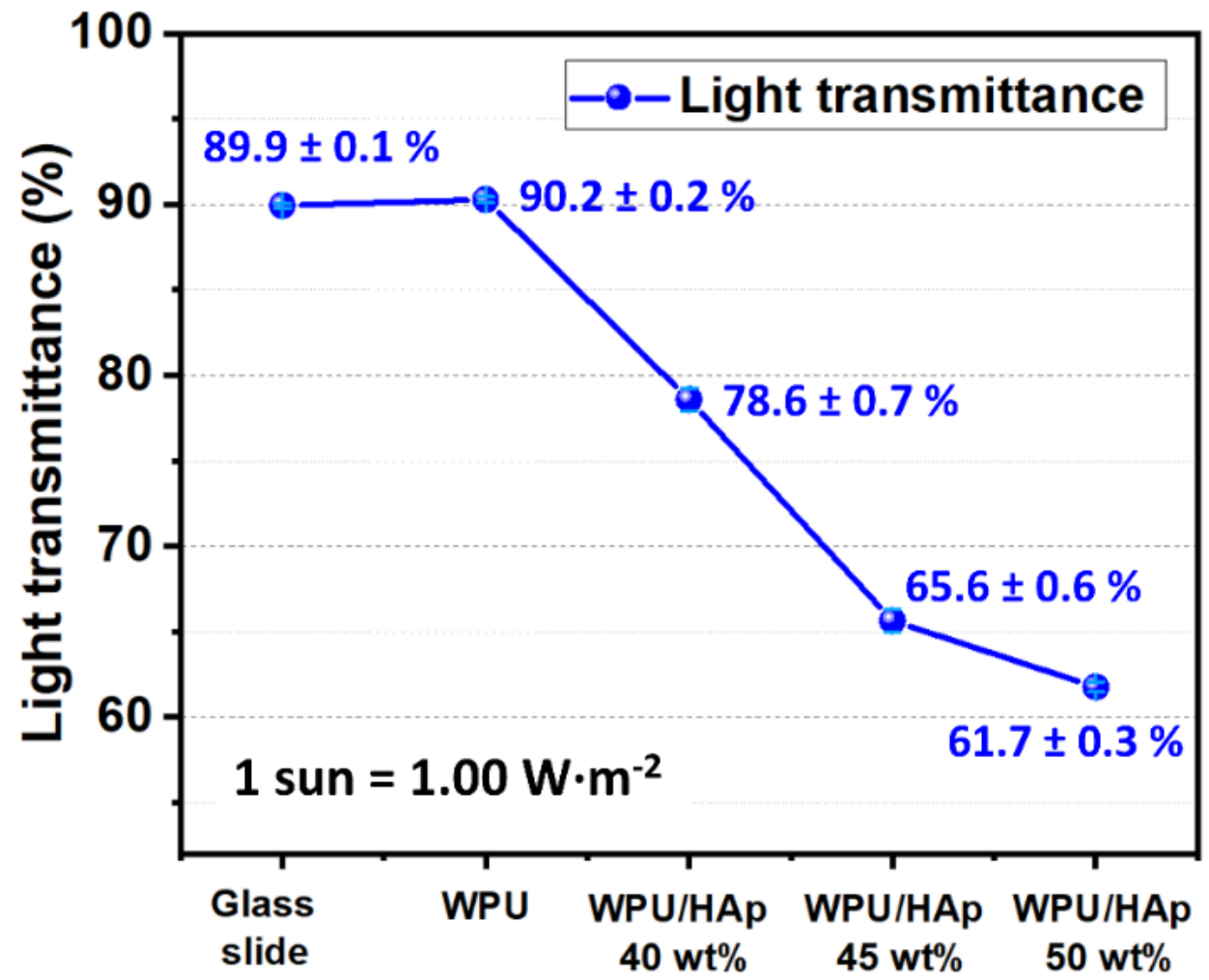

Figure S30: Light transmittance of WPU/HAp 40, 45, and 50 wt%. Three replicates of the 2.5×2.5 cm^2-sized samples were tested to obtain the statistical data.

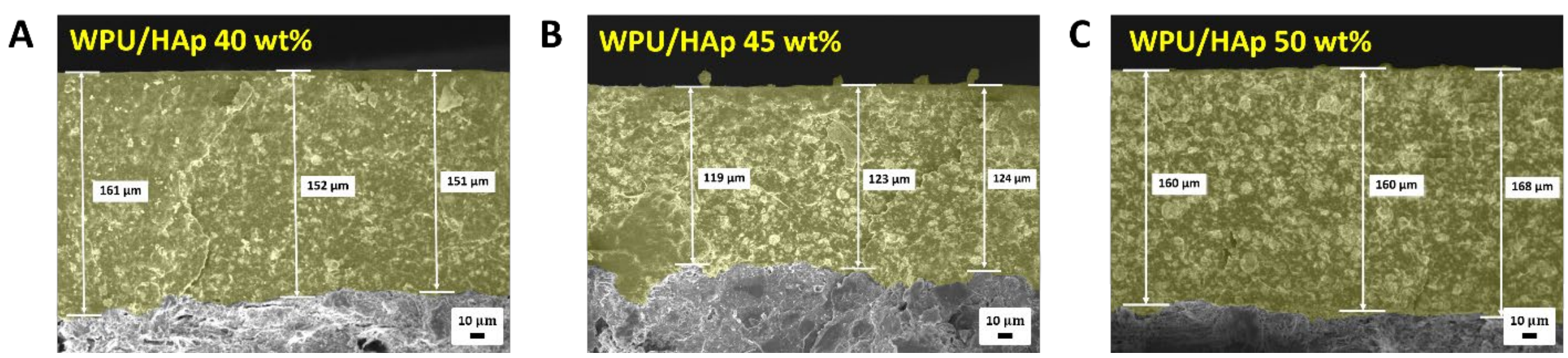

Figure S31: Cross-section FESEM images of WPU/HAp. A.) WPU/HAp - 40 wt%. B.) WPU/HAp - 45 wt%. C.) WPU/HAp - 50 wt%. Scale bars represent 10 µm.

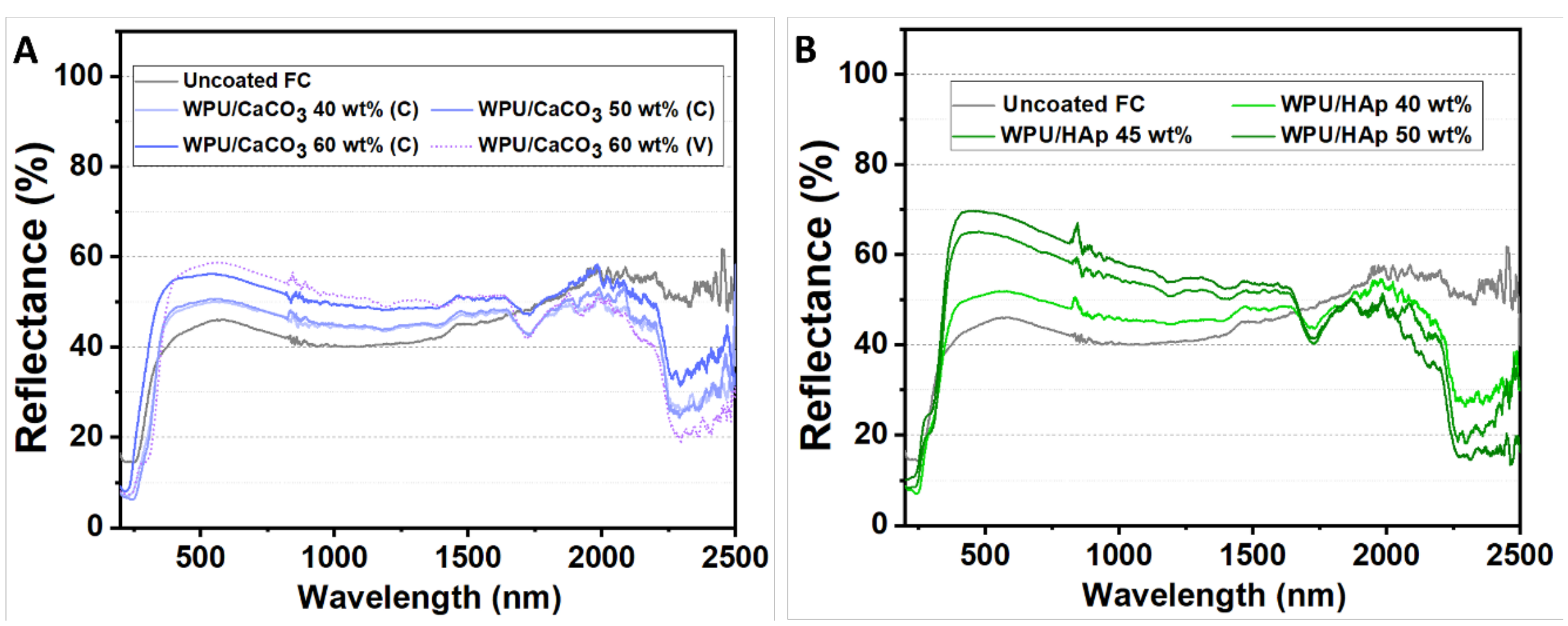

Figure S32: Comparison of reflectance spectra. A.) uncoated FC and WPU/$CaCO_3$ in 40 – 60 wt%. B.) uncoated FC and WPU/HAp in 40 – 50 wt%. (C) – cross-direction painting, (V) – vertical direction painting. The cross-directional and vertical-directional painting methods exhibited no significant difference in reflectance. However, the vertical direction improved water runoff, which facilitated easier cleaning and reduced surface accumulation. Hence, the WPU/HAp series was prepared via vertical direction painting.

Table S4: Summary of reflectance and emissivity values of WPU/HAp coatings at different concentrations.

HAp Concentration	Reflectance				Emissivity (8-13 µm)
	Solar (300-2500 nm)	UV (300-400 nm)	Visible (400-700 nm)	IR (700-2500 nm)	
40 wt%	48.4	43.6	51.3	46.7	94.4
45 wt%	57.7	54.6	64.0	53.6	94.9
50 wt%	61.4	59.7	68.6	56.6	96.0

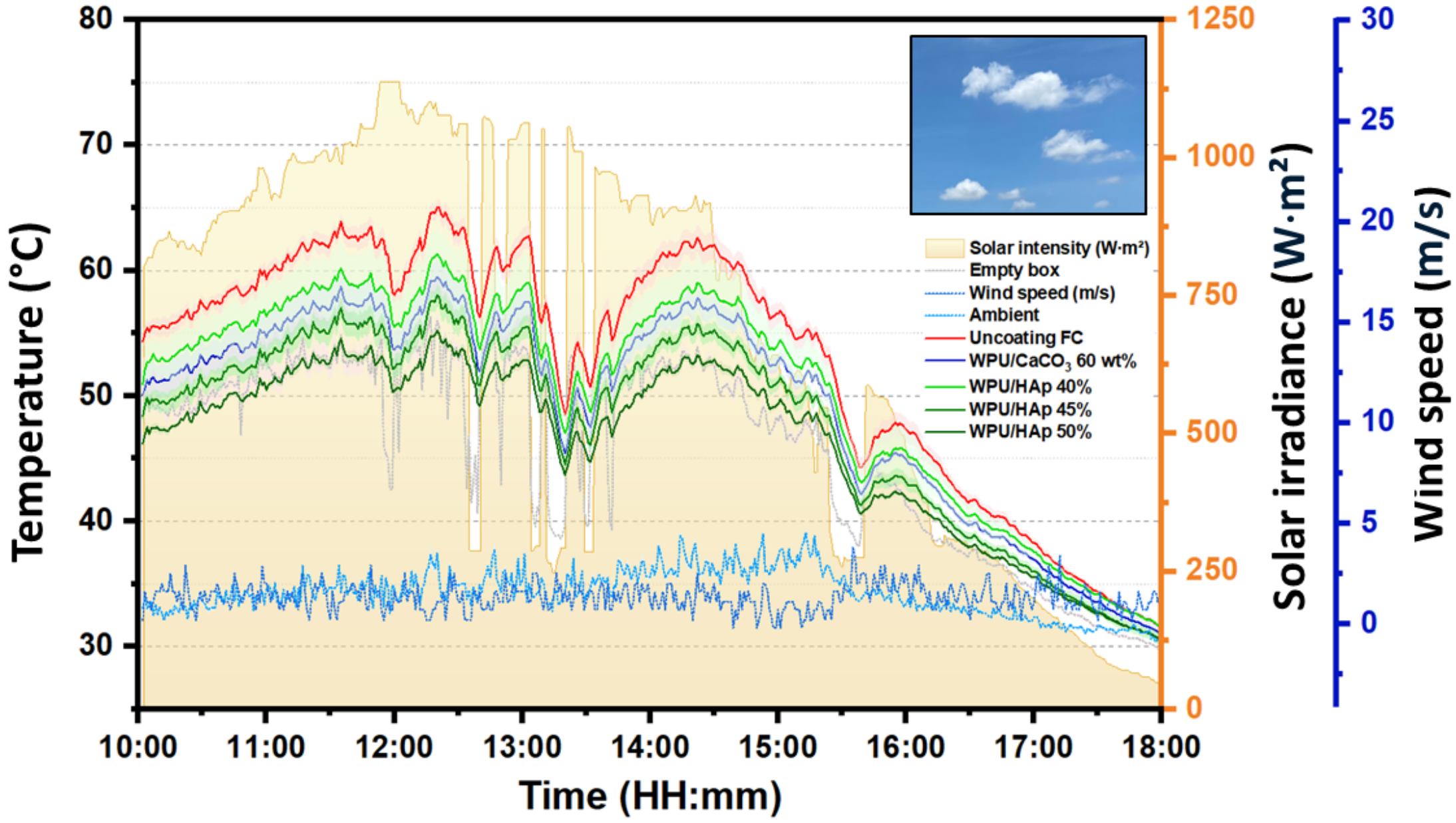

Figure S33: The full day temperature profiles to compare temperature reduction between WPU/$CaCO_3$ – 60 wt% coating and WPU/HAp coatings at different concentrations. Three replicates of the 8×8 cm^2-sized samples were tested to obtain the statistical data. The results are presented as the mean (central line) with standard deviation (S.D.) (shaded contour region).

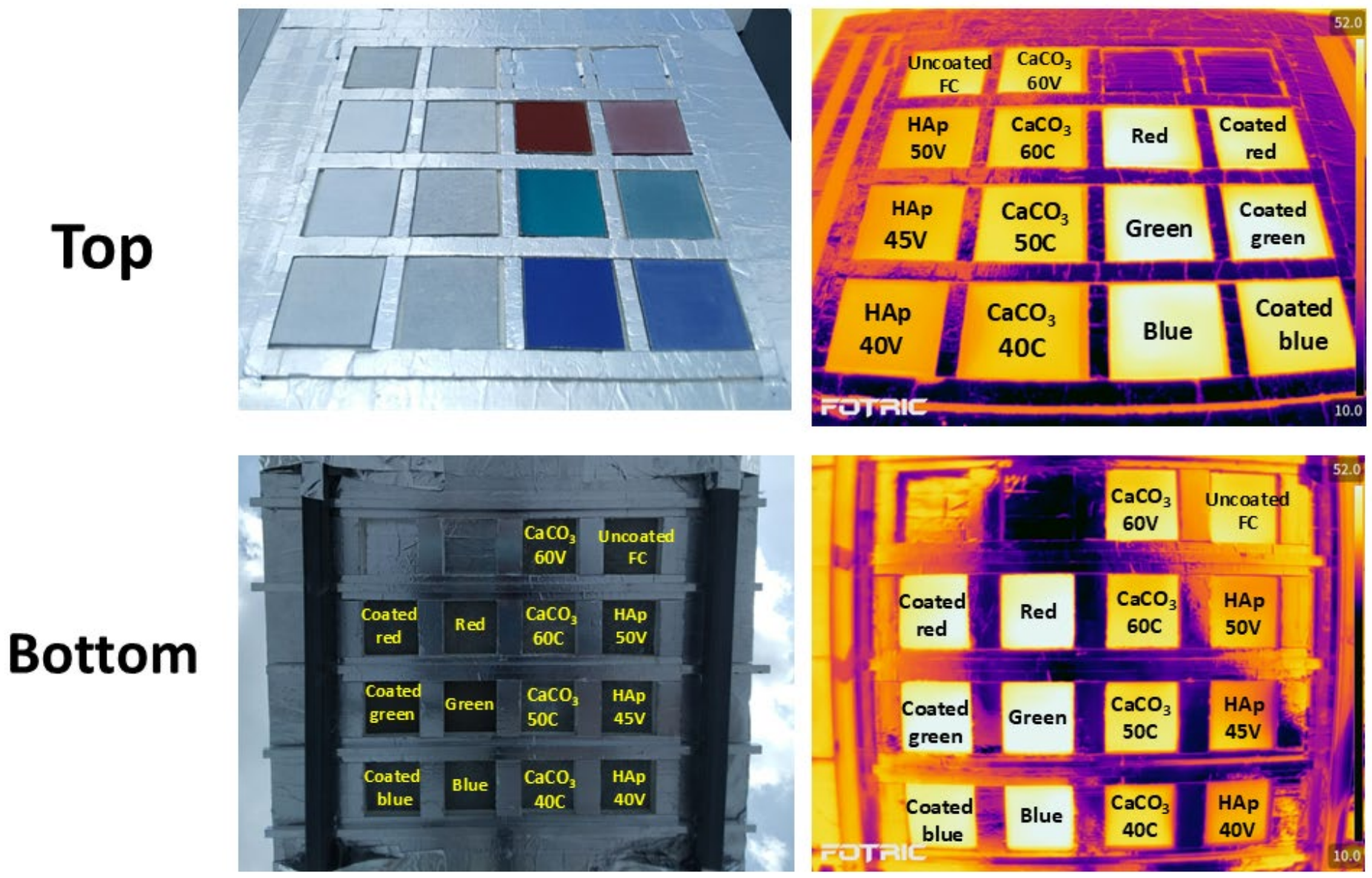

Figure S34: Photographs and infrared thermography images comparing WPU/$CaCO_3$ series and WPU/HAp series from both top and bottom sides of 8×8 cm^2-sized samples.

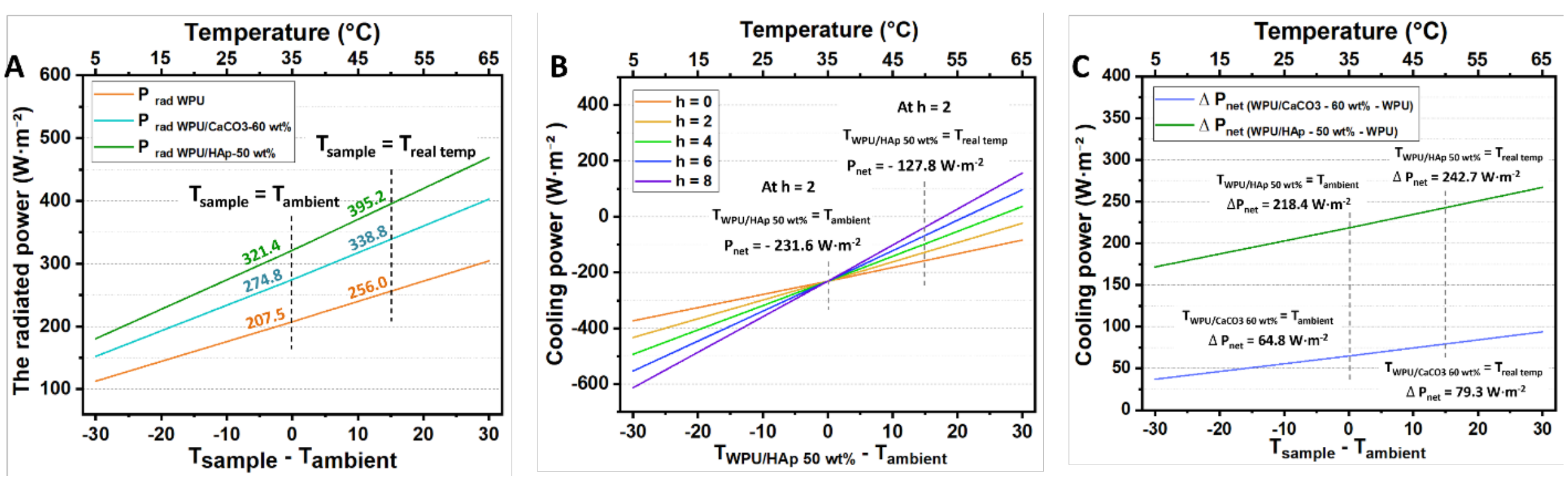

Figure S35: Calculation of heat flux and cooling power. A.) Calculated heat flux of WPU, WPU/$CaCO_3$ – 60 wt%, and WPU/HAp – 50 wt%. B.) Cooling power as a function of temperature difference of $T_{WPU/HAp\ 50\ wt\%}$ - $T_{ambient}$. C.) Cooling power difference between WPU/$CaCO_3$ and WPU/HAp.

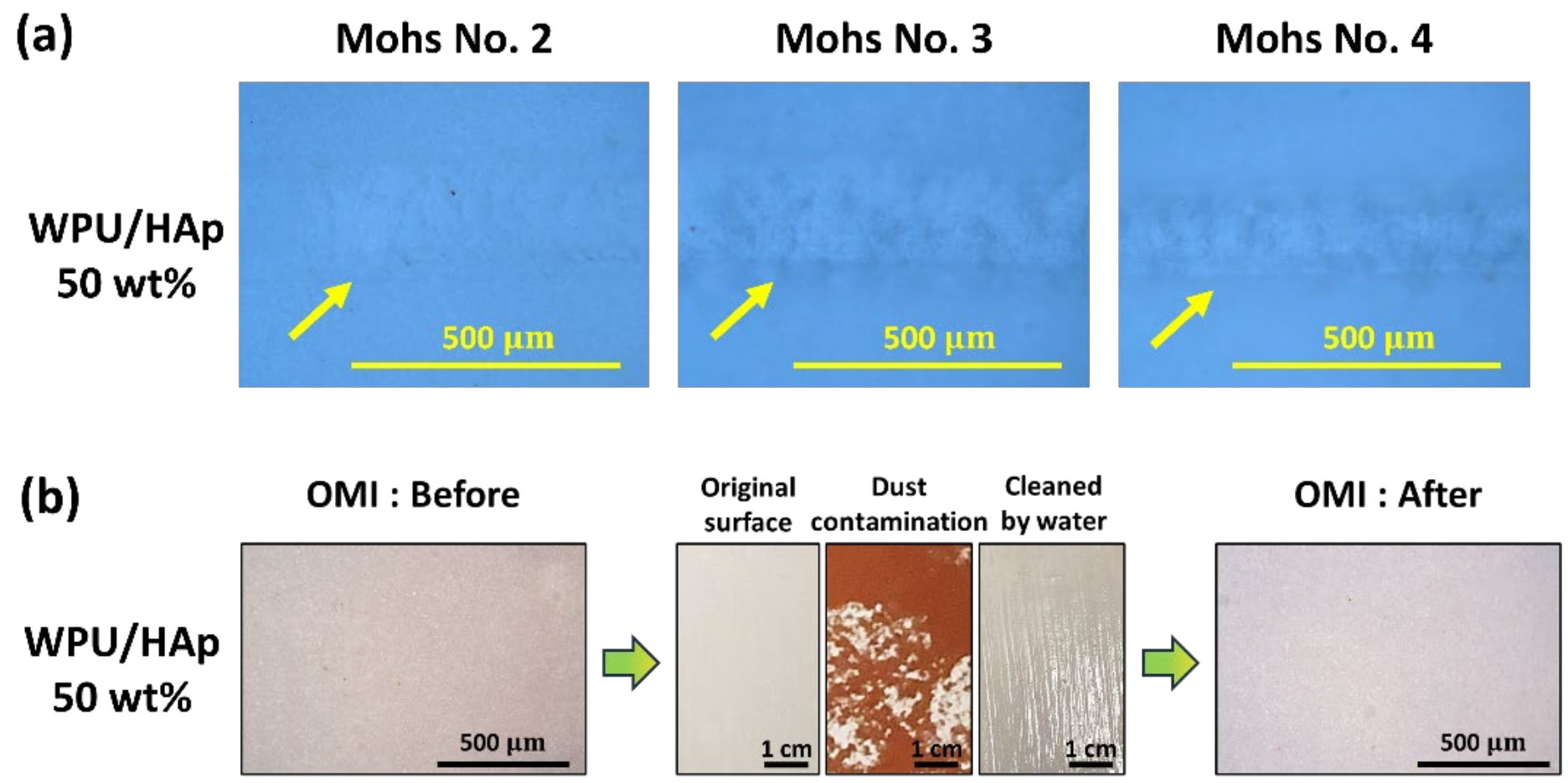

Figure S36: Mohs hardness test and dust-washability of WPU/HAp – 50 wt%. A.) the Mohs hardness test (scale bars represent 500 µm). B.) Anti-fouling test of WPU/HAp - 50 wt% coated fiber cement (scale bars represent 500 µm for optical microscopic images and scale bars represent 1 cm for photographs).

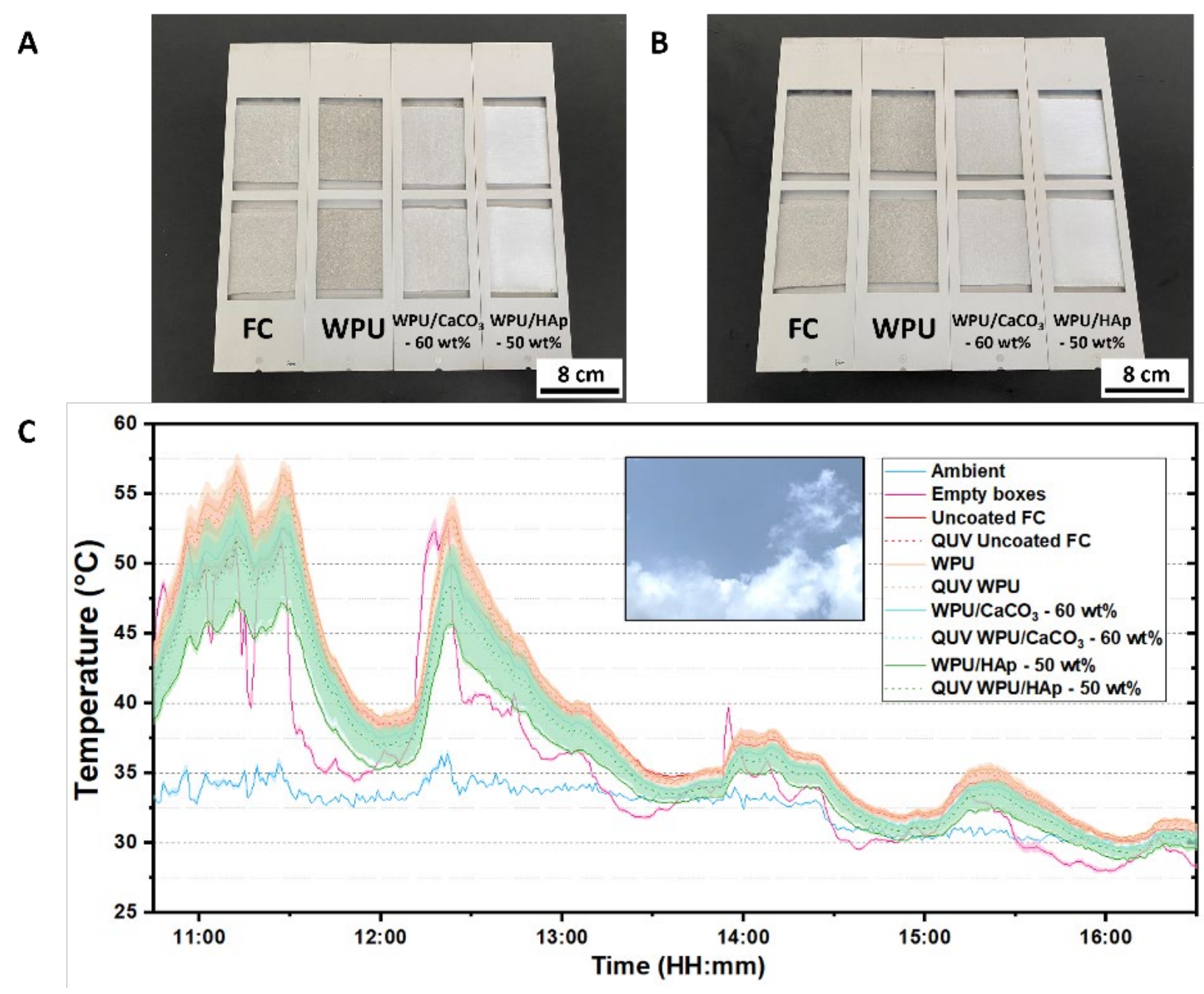

Figure S37: The photograph of coated roof tiles before and after QUV weathering test and temperature profile. A.) Before QUV weathering test. B.) After QUV weathering test. The represent scale bars represent 8 cm. C.) The full day temperature profiles to compare temperature reduction of WPU, WPU/$CaCO_3$ – 60 wt% coating and WPU/HAp - 50 wt% before and after QUV weathering test.

Supplemental references

1. So, S., Yun, J., Ko, B., Lee, D., Kim, M., Noh, J., Park, C., Park, J., and Rho, J. (2024). Radiative Cooling for Energy Sustainability: From Fundamentals to Fabrication Methods Toward Commercialization. Adv. Sci. *11*. https://doi.org/10.1002/advs.202305067.

2. Luo, M., Liao, J., Wei, X., Jia, S., Lin, Y., Liu, W., Zhou, L., Zou, Q., and Wang, S. (2025). High-performance radiative cooling PVDF-HFP film based on controllable porous structure. Prog. Org. Coat. *199*, 108901. https://doi.org/10.1016/j.porgcoat.2024.108901.

3. Zhou, L., Rada, J., Tian, Y., Han, Y., Lai, Z., McCabe, M.F., and Gan, Q. (2022). Radiative cooling for energy sustainability: Materials, systems, and applications. Phys. Rev. Mater. *6*, 090201. https://doi.org/10.1103/PhysRevMaterials.6.090201.

4. Akram, N., Saeed, M., and Usman, M. (2022). Role of Macrodiols in the Synthesis and Thermo-Mechanical Behavior of Anti-Tack Water Borne Polyurethane Dispersions. Polymers (Basel). *14*, 572. https://doi.org/10.3390/polym14030572.

5. Sidra, Tabasum, S., Zia, K.M., Parveen, B., and Hussain, M.T. (2021). A novel water borne green textile polyurethane dispersions finishes from cotton (Gossypium arboreum) seed oil based polyol used in modification of cellulosic fabrics. Carbohydr. Polym. Technol. Appl. *2*, 100170. https://doi.org/10.1016/j.carpta.2021.100170.

6. Kim, H.N., Kim, J.W., Kim, M.S., Lee, B.H., and Kim, J.C. (2019). Effects of Ball Size on the Grinding Behavior of Talc Using a High-Energy Ball Mill. Minerals *9*, 668. https://doi.org/10.3390/min9110668.

7. Song, J., Qin, J., Qu, J., Song, Z., Zhang, W., Xue, X., Shi, Y., Zhang, T., Ji, W., Zhang, R., et al. (2014). The effects of particle size distribution on the optical properties of titanium dioxide rutile pigments and their applications in cool non-white coatings. Sol. Energy Mater. Sol. Cells. *130*, 42–50. https://doi.org/10.1016/j.solmat.2014.06.035.
